# Albumin Replacement Therapy in Septic Shock: A Randomized Clinical Trial

**DOI:** 10.1001/jamanetworkopen.2025.59297

**Published:** 2026-02-19

**Authors:** Yasser Sakr, Axel Nierhaus, Ulrike Schumacher, Stefan Utzolino, Ulrich Jaschinski, Sirak Petros, Falk Fichtner, Christine Eimer, Christian Putensen, Ivan Tanev, Lukas Kreienbühl, Stefan Kluge, Lampros Kousoulas, Sven-Olaf Kuhn, Dominik Jarczak, Michael Quintel, Michael Bauer

**Affiliations:** 1 Department of Anesthesiology and Intensive Care Therapy, Jena University Hospital, Jena, Germany; 2 Department of Critical Care Medicine, Sheikh Shakbout Medical City, Abu Dhabi, United Arab Emirates; 3 Department of Intensive Care Medicine, Hamburg-Eppendorf University Hospital, Hamburg, Germany; 4 Center for Clinical Studies, Jena University Hospital, Jena, Germany; 5 Department of General Surgery, Surgical Intensive Care Unit, Freiburg University Hospital, Freiburg, Germany; 6 Department of Anesthesiology and Surgical Intensive Care Therapy, Augsburg University Hospital, Augsburg, Germany; 7 Interdisciplinary Medical Intensive Care Unit, Leipzig University Hospital, Leipzig, Germany; 8 Department of Anesthesiology and Intensive Care Therapy, Leipzig University Hospital, Leipzig, Germany; 9 Department of Anesthesiology and Intensive Care Medicine, University Medical Center Schleswig-Holstein, Campus Kiel, Kiel, Germany; 10 Department of Anesthesiology and Surgical Intensive Care, Bonn University Hospital, Bonn, Germany; 11 Department of Cardiology and Angiology, Magdeburg University Hospital, Magdeburg, Germany; 12 Department of Anesthesiology, Critical Care, & Perioperative Pain Management, Helios Klinikum, Bad Saarow, Germany; 13 Department of Anesthesiology, Intensive Care, Emergency and Pain Medizin, Klinik für Anästhesie, Intensiv-, Notfall- und Schmerzmedizin, Greifswald University Hospital, Greifswald, Germany; 14 Department of Anesthesiology, University Medical Center Göttingen, Göttingen, Germany

## Abstract

**Importance:**

Albumin supplementation may reduce mortality in patients with septic shock; however, data from randomized clinical trials are limited.

**Objective:**

To assess the impact of albumin administration on outcomes in patients with septic shock.

**Design, Setting, and Participants:**

This multicenter, open-label randomized clinical trial was conducted between October 21, 2019, and May 2, 2022. Patients from 23 intensive care units in Germany enrolled within 24 hours of the onset of septic shock were followed up for outcome data up to 90 days. The statistical trial report was completed and filed with the federal authorities in December 2023; additional analyses were completed in October 2024. The study was terminated prematurely due to low enrollment rates.

**Interventions:**

Protocol group patients received 20% albumin to maintain serum albumin levels of at least 3.0 g/dL for up to 28 days during their intensive care unit admission. The control group received standard fluid administration with crystalloids.

**Main Outcomes and Measures:**

The primary end point was 90-day mortality; secondary end points included 28-day, 60-day, intensive care unit and in-hospital mortality, organ dysfunction or failure, total amount of fluid administration and total fluid balance while in the intensive care unit, duration of intensive care and hospital stays, and frequency of adverse events.

**Results:**

Of 440 randomized patients (median [IQR] age, 69 [59-78] years; 290 [65.9%] male), 222 received albumin and 218 received standard fluids. Baseline characteristics were comparable. Ninety-day mortality was 43.3% (91 of 210) in the albumin group vs 45.9% (96 of 209) in controls (relative risk, 0.94; 95% CI, 0.76-1.17; *P* = .71). No significant differences were observed for secondary end points.

**Conclusions and Relevance:**

In this randomized clinical trial of patients with septic shock, albumin administration was safe but did not improve 90-day survival. As this trial was prematurely terminated, results remain inconclusive and additional studies are recommended.

**Trial Registration:**

ClinicalTrials.gov Identifier: NCT03869385

## Key Points

**Question:**

Can the reported potential mortality reduction by albumin replacement in septic shock be confirmed in a randomized clinical trial?

**Findings:**

In a multicenter randomized clinical trial, 440 adults with septic shock were treated with albumin therapy aiming to maintain serum albumin concentrations greater than 3.0 g/dL or with standard fluid therapy. Ninety-day mortality did not differ significantly between the albumin group (43.3%) and the controls (45.9%).

**Meaning:**

These results suggest that albumin administration was safe but did not improve 90-day survival in patients with septic shock; results remain inconclusive due to premature trial termination.

## Teaser

This randomized clinical trial of patients with septic shock assesses whether albumin reduces 90-day mortality.

## Introduction

Albumin, a natural colloid, has multiple properties in addition to its role in maintaining plasma oncotic pressure. These properties include binding and transport of endogenous molecules and medications,^1^ anti-inflammatory^2^ and antioxidant effects,^3^ and modulation of nitric oxide metabolism.^4^ These attributes have provided a rationale for the use of human albumin in critically ill patients for decades, despite conflicting evidence.^5,6,7,8^ In the landmark SAFE (Saline Albumin Fluid Evaluation) study,^8^ overall mortality rates were similar in the 6997 critically ill patients randomized to receive volume replacement therapy with either 4% human albumin or 0.9% saline. However, in a predefined subgroup of 1218 patients with sepsis, there was a trend toward lower mortality in those receiving albumin. A pilot study^9^ in a mixed population of critically ill patients targeting albumin administration to maintain serum albumin concentrations above 3.0 g/dL (to convert grams per liter, multiply by 10) suggested improved organ function. This prompted the Albumin Italian Outcome Sepsis (ALBIOS) study,^10^ which randomized 1810 patients with sepsis or septic shock to receive either 20% human albumin solution to maintain serum albumin concentrations of 3.00 g/dL or greater or crystalloid solution. Although no differences in outcome were seen, a potential survival benefit was reported in patients who received albumin therapy 6 to 24 hours after diagnosis of sepsis compared with those who received it earlier. Relative risk for 90-day mortality was reduced in the subset of albumin-treated patients with septic shock at the time of enrollment.^10^ Albumin administration may thus provide a patient-centered benefit in some patients with sepsis.

The ARISS (Albumin Replacement in Septic Shock) trial was designed to investigate the effect of albumin replacement on mortality in patients with septic shock. We hypothesized that albumin administration started within 6 to 24 hours after the onset of septic shock and titrated to maintain a serum albumin concentration of 3.0 g/dL or greater for up to 28 days during an intensive care unit (ICU) stay would reduce 90-day all-cause mortality.

## Methods

### Study Design and Oversight

The ARISS study was a prospective, multicenter, controlled, parallel-group, open-label, interventional randomized clinical trial. The trial was conducted in agreement with the German Medicinal Products Act, Good Clinical Practice in Conducting Clinical Trials with Medicinal Products,^11^ and the ethical principles set out in the Declaration of Helsinki.^12^ The trial protocol can be found in Supplement 1. The main ethics committee was based at the sponsor site (University of Jena, Jena, Germany). Ethics committees at each contributing center approved the study protocol before recruitment of patients. A list of contributing centers is provided in eTable 2 in Supplement 2. Trial monitoring was performed by the Center for Clinical Studies in Jena, Germany. A data and safety monitoring board received all safety-related data and conducted independent reviews. The study protocol has been published previously.^13^ This report follows the updated Consolidated Standards of Reporting Trials (CONSORT) reporting guidelines.^14^

### Patients

Adult patients (aged ≥18 years) admitted to a participating ICU were screened daily and considered for inclusion if they met all the following criteria: (1) the presence of clinically possible, probable, or microbiologically confirmed infection according to the criteria developed by the International Sepsis Forum^15^; (2) need for vasopressors to maintain a mean arterial pressure of 65 mm Hg or higher for at least 1 hour despite adequate volume therapy^16^; (3) serum lactate concentration higher than 18 mg/dL (to convert to millimoles per liter, multiply by 0.111); and (4) diagnosis of septic shock less than 24 hours before study inclusion, enabling administration of the first dose of albumin to be given within 6 to 24 hours after the onset of septic shock. The baseline albumin level was not considered an inclusion criterion because the intervention aimed to replace and maintain albumin using adapted treatment schemes. Exclusion criteria were (1) moribund conditions with life expectancy less than 28 days or palliative care with a life expectancy of less than 6 months; (2) presence of an end-of-life decision; (3) previous participation in the study; (4) participation in another interventional clinical trial within the previous 3 months; (5) history of hypersensitivity to albumin or any other component of the trial product; (6) disease in which the use of albumin may be deleterious (eg, decompensated heart failure or traumatic brain injury); (7) disease in which albumin administration may be advantageous (eg, hepatorenal syndrome); and (8) pregnancy or lactation.

Eligible patients were included in the study after obtaining informed consent from the patient, guardian, or legal representative. In addition, according to German regulations, trial enrollment was legally possible without informed consent if this could not be obtained in an emergency situation. However, written consent by the legal representative had to be obtained within 72 hours of study entry.^17^ If this proved impossible, the patient’s participation in the trial was terminated.

Patients from 23 study centers were enrolled between October 21, 2019, and May 2, 2022, with study follow-up period completed on July 27, 2022. The study was stopped prematurely because of low enrollment rates. Patients were randomized to the treatment or control group in a 1:1 ratio. Randomization was performed by an automated internet-based service based on a randomization list created in advance with nQuery Advisor 7.0 (Informer Technologies Inc) and was stratified according to serum lactate levels within 24 hours before trial inclusion (≤81.08 vs >81.08 mg/dL) and by trial center.

### Study Treatments

In the treatment group, patients received a 60-g loading dose of 20% human albumin during 2 to 3 hours within 6 to 24 hours after the diagnosis of septic shock and within 2 hours after randomization. Treating physicians were not blinded regarding the intervention. Serum albumin concentrations were maintained at 3.0 g/dL or higher for a maximum of 28 days while in the ICU using 40- to 80-g infusions of 20% human albumin. To achieve this target, the following scheme was applied with serum albumin concentrations measured once daily: serum albumin of 3.0 g/dL or higher, no albumin administration; 2.5 g/dL or higher and less than 3.0 g/dL, 40 g during 1 to 2 hours; 2.0 g/dL or higher and less than 2.5 g/dL, 60 g during 2 to 3 hours; and less than 2.0 g/dL, 80 g during 3 to 4 hours. The control group was treated according to standard of care; this included administration of albumin in agreement with current guidelines for resuscitation from septic shock if deemed necessary by the treating physician.

All patients received fluid resuscitation with crystalloids. There were no restrictions to concomitant medications or therapies during the trial. Patients were followed up to 90 days after randomization. An overview of the study procedures is given in eFigure 1 in Supplement 2.

Data entry, processing, and evaluation were performed at the Center for Clinical Studies in Jena, Germany, using deidentified patient-related data provided by the trial centers. Data accuracy was verified with range, validity, and consistency checks.

### Outcome Measures

Illness severity on ICU admission was assessed using the Simplified Acute Physiology Score (SAPS) II.^18^ Organ function was assessed daily for up to 28 days in the ICU after randomization using the Sequential Organ Failure Assessment (SOFA) score,^19^ with subscores for each of 5 organ systems (respiratory, coagulation, liver, cardiovascular, and kidney). Due to difficulties with interpretation of the Glasgow Coma Scale in sedated patients, the neurologic subscore was excluded. Organ dysfunction was defined as a change in baseline SOFA score of 2 points or more.

The primary end point was 90-day all-cause mortality. Secondary end points were 28- and 60-day mortality, ICU and hospital mortality, organ dysfunction or failure as assessed by the SOFA score, ICU and hospital length of stay, ventilator- and vasopressor-free days, total amount of fluid administration and total fluid balance in the ICU, and safety-related parameters (ie, occurrence of adverse events [AEs] and serious AEs). Drug-related AEs were recorded in the albumin group until 24 hours after the final dose of the trial drug and in the control group until day 28 after randomization or until discharge from the ICU, whichever occurred earlier. The severity of each AE was classified as serious or not and the intensity stratified as mild, moderate, or severe (eTable 1 in Supplement 2); sepsis-related clinical events were only documented as AEs if the examiner suspected a connection with administration of the trial drug.

### Statistical Analysis

Sample size estimation was performed using SAS software, version 9.4 (SAS Institute Inc). Ninety-day mortality in the control arm was estimated at 50%.^20,21,22^ A 15% reduction in 90-day mortality in the treatment group was assumed (ie, an absolute reduction of 7.5 percentage points to 42.5%). A Mantel-Haenszel χ^2^ test with a 2-tailed significance level of *P* < .05 and a power of 80% required 1412 patients to be analyzed (706 per arm). Assuming a dropout rate of 15%, 1662 patients needed to be randomized.

The modified intention-to-treat population included all patients enrolled in the study and randomized with at least one observation made after randomization; all patients met this criterion. Primary efficacy and safety analyses were performed in the intention-to-treat population. As a sensitivity analysis, the primary efficacy analysis was repeated in the per-protocol population. Because 106 control patients (48.6%) received albumin for early resuscitation (as allowed at the physician’s discretion according to the study protocol), we performed the sensitivity analysis on this considerably diminished population and on a modified per-protocol population, in which only patients with major study plan deviations apart from albumin administration were excluded.

All collected data were analyzed using descriptive methods in the 2 study groups. The primary end point, 90-day mortality, was analyzed using a generalized mixed model with fixed factors of baseline lactate, SAPS II score, type of admission, serum albumin, and treatment group, with a 2-sided α = .05. The model was set up with maximum-pseudolikelihood, subject-specific expansion method and with a variance-component covariance structure method. However, because the random factor site had no effect (*P* > .99) and was excluded from the model, the model reduced to a generalized linear model. All secondary end points, unless included in the primary analysis model, were exploratively compared between groups using descriptive statistics including 95% CIs. The 28- and 60-day mortality were evaluated with models analogous to the primary analysis.

The primary end point and selected secondary end points were described for the following subgroups: baseline lactate (≤81.08 vs >81.08 mg/dL) and different SOFA (2-9, 10-14, or 15-24), SAPS II (≤39, 40-79, or 80-163), and Acute Physiology and Chronic Health Evaluation (APACHE) II (≤19, 20-29, or 30-71) scores. No interim analysis was planned. No imputation of missing data was performed.

## Results

### Study Population

**Figure 1.**
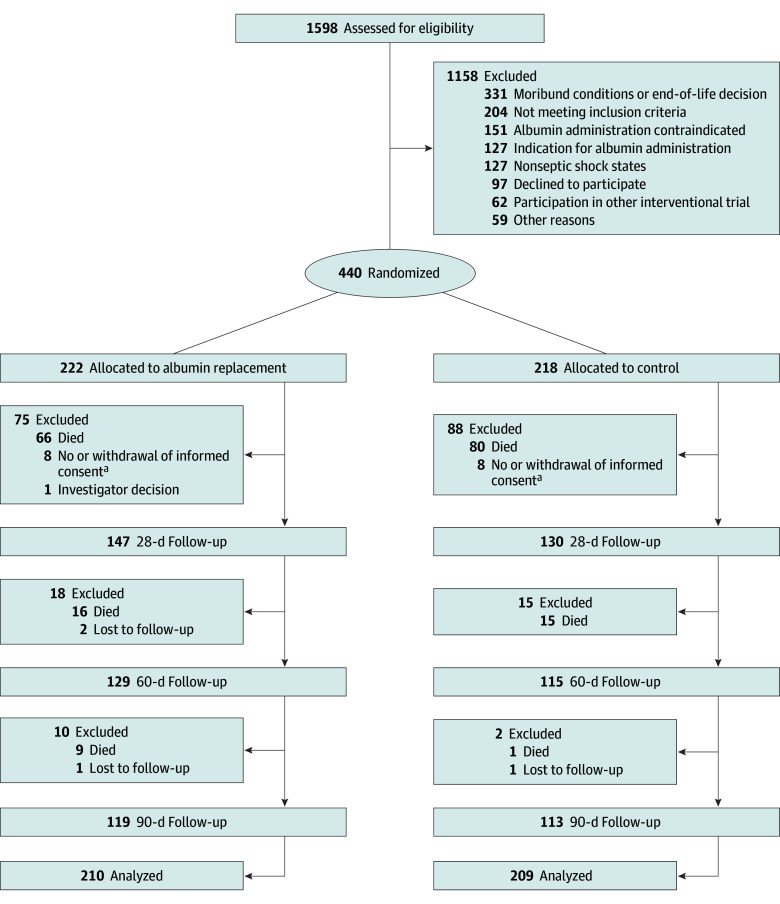
CONSORT Flowchart of Trial Participation CONSORT indicates Consolidated Standards of Reporting Trials. ^a^No informed consent: German regulations allow inclusion based on medical consultant decision provided that informed consent of legal representative or guardian is obtained within 72 hours thereafter, which failed in some cases.

During the study period, 440 patients (median [IQR] age, 69 [59-78] years; 290 [65.9%] male and 150 [34.1%] female) were enrolled (eTable 2 in Supplement 2), with 222 randomized to receive albumin and 218 to the control group. Informed consent could not be obtained within 72 hours after randomization or was withdrawn in 16 patients. One patient was excluded from the study based on investigator decision; 4 patients were lost to follow-up. Therefore, the primary outcome variable was available for analysis in 210 patients in the albumin group and 209 patients in the control group (Figure 1).

Admission to the ICU was for medical reasons in 166 patients (37.7%), elective surgery in 62 (14.1%), and emergency surgery in 212 (48.2%). The most common sources of infection were abdominal (160 [36.4%]) and pulmonary (109 [24.8%]). Baseline characteristics (Table 1) as well as source and mode of acquisition of infection (eTables 3 and 4 in Supplement 2) were similar between the 2 groups.

**Table 1.** Baseline Patient Characteristics

Characteristic	No. (%) of patients^a^	
	Albumin group (n = 222)	Control group (n = 218)
Age, median (IQR), y	70 (60 to 77)	69 (59 to 78)
Sex		
Male	142 (64.0)	148 (67.9)
Female	80 (36.0)	70 (32.1)
BMI, median (IQR)	26.22 (23.23 to 31.14)	26.69 (23.77 to 30.86)
Reason for ICU admission		
Elective surgery	29 (13.1)	33 (15.1)
Emergency surgery	111 (50.0)	101 (46.3)
Medical	82 (36.9)	84 (38.5)
Preexisting condition		
Arrhythmias	42 (19.0)	44 (20.5)
Coronary heart disease	41 (18.6)	46 (21.4)
Valvular heart disease	31 (14.1)	19 (8.8)
Immunosuppressive therapy	21 (9.5)	11 (5.1)
COPD	18 (8.1)	23 (10.6)
Renal failure, requiring dialysis	18 (8.1)	12 (5.6)
Long-term alcohol abuse	16 (7.5)	22 (10.7)
Metastatic cancer	15 (6.9)	20 (9.6)
Liver cirrhosis	7 (3.2)	16 (7.4)
Chronic heart failure (NYHA IV)	6 (2.7)	2 (0.9)
SAPS II score, median (IQR)	54 (45 to 64)	54 (43 to 63)
SOFA score ‖, median (IQR)	11 (9 to 13)	10 (8 to 13)
APACHE II score, median (IQR)	22 (18 to 29)	22 (17 to 27)
Organ failure^b^		
1	9 (4.1)	8 (3.7)
2	96 (43.2)	88 (40.4)
3	83 (37.4)	91 (41.7)
4	29 (13.1)	25 (11.5)
>4	5 (2.3)	6 (2.8)
Physiologic parameters^c^		
Heart rate, median (IQR), /min	123 (106 to 184)	121 (106 to 138)
Mean arterial pressure, median (IQR), mm Hg	57 (51 to 62)	57 (52 to 62)
Central venous pressure, median (IQR), mm Hg	9 (7 to 11)	9 (7 to 10)
Lactate, median (IQR), mg/dL	44.14 (32.43 to 67.57)	45.05 (29.73 to 72.07)
Serum albumin, median (IQR), g/dL	2.2 (1.8 to 2.7)	2.2 (1.8 to 2.6)
Hemoglobin, median (IQR), g/dL	9.5 (8.2 to 11.3)	9.5 (8.2 to 11.1)
Procedures		
Mechanical ventilation		
Noninvasive	21 (9.5)	12 (5.5)
Invasive	143 (64.4)	151 (69.3)
Renal replacement therapy	44 (19.8)	33 (15.1)
Extracorporeal membrane oxygenation	5 (2.3)	2 (0.9)
Fluid administration in first 24 h in ICU, median (IQR), mL	8484 (5390 to 12 300)	8190 (5158 to 11 987)
Fluid balance in first 24 h in ICU, median (IQR), mL	6320 (3712 to 10 155)	6015 (3372 to 9575)
Time to first antibiotic administration at randomization, median (IQR), h	−0.8 (−5.6 to +0.9)	−1.2 (−6.6 to +0.9)

Abbreviations: APACHE, Acute Physiology and Chronic Health Evaluation; BMI, body mass index (calculated as weight in kilograms divided by height in meters squared); COPD, chronic obstructive pulmonary disease; ICU, intensive care unit; NYHA, New York Heart Association classification; SAPS II, Simplified Acute Physiology; SOFA, Sequential Organ Failure Assessment.

SI conversion factors: To convert lactate to millimoles per liter, multiply by 0.111; albumin to grams per liter, multiply by 10; hemoglobin to grams per liter, multiply by 10.

^a^ Unless otherwise indicated.

^b^ Organ failure was defined as a SOFA score of 2 points or more for the respective SOFA subscore.

^c^ Values obtained ±1 hour from randomization.

### Study Intervention

In the albumin group, 2 of the 220 patients died before study medication was commenced but were included in the full analysis on an intention-to-treat basis. The median (IQR) duration of albumin administration was 5 (2-17) days, with 15 patients treated for the maximum of 28 days. The mean (SD) amount of albumin administered was 229 (50) mL/d.

**Figure 2.**
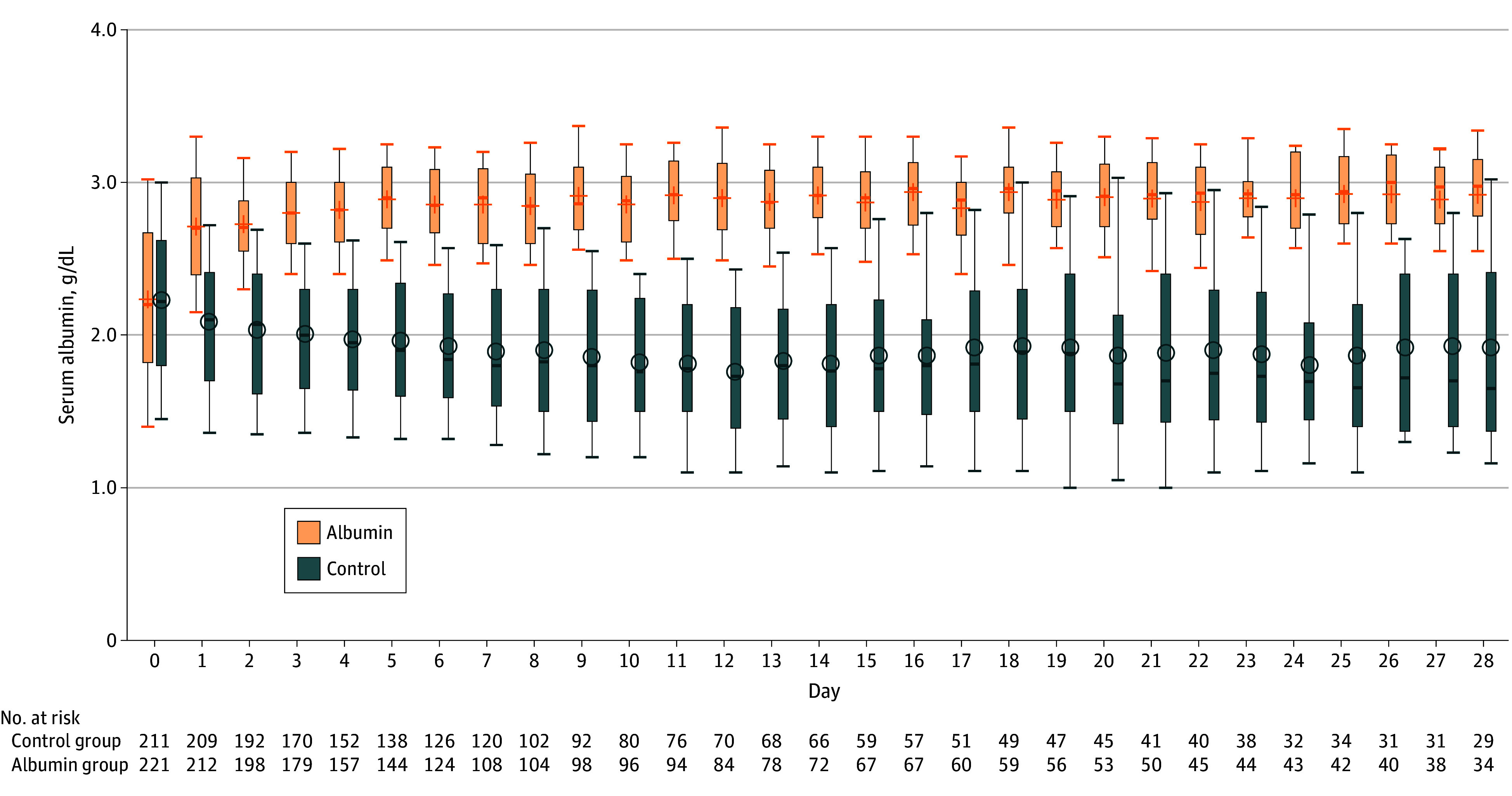
Box Plot Showing Daily Serum Albumin Concentration in the Study Groups SI conversion factor: To convert albumin to grams per liter, multiply by 10.

In the albumin group, 178 patients had a protocol deviation, 2 of which were major. Although separation was achieved between groups (Figure 2), the target plasma albumin level of 3.0 g/dL in the treatment group was not reached in more than half the patients during the entire period (eTable 5 in Supplement 2). In the control group, 151 patients had a protocol deviation, 10 of which were major (eTable 6 in Supplement 2). Albumin was given to 106 patients (48.6%) in the control group (twice in 1 patient) during their ICU stay, as deemed necessary by the attending physician. Given this high number, we performed a per-protocol and a modified per-protocol analysis. The former analysis excluded all patients with major protocol deviations, including albumin administration; the latter included the 428 patients without major protocol deviations except albumin administration (220 in the treatment group and 208 in the control group).

Before randomization, patients in the albumin group received similar amounts of fluid compared with the control group (median [95% CI], 8484 [7677-9774] vs 8190 [7378-8736] mL, respectively). Physiologic and oxygenation parameters, fluid administration volumes, vasopressor therapy, and procedures were similar between the 2 groups at 6 hours after randomization (eTable 7 in Supplement 2).

### Outcomes

**Figure 3.**
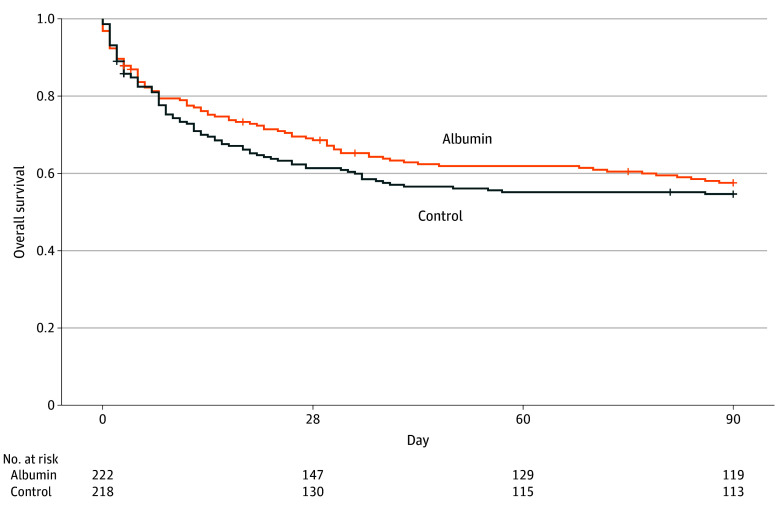
Kaplan-Meier Curves for Survival of Patients Receiving Albumin vs Control Treatment

The primary end point, 90-day mortality, was similar between the treatment and control groups (91 of 210 [43.3%] vs 96 of 209 [45.9%]; relative risk, 0.94; 95% CI, 0.76-1.17; *P* = .71). Kaplan-Meier survival analysis showed no difference between study groups up to 90 days after randomization (Figure 3).

The 28-day (66 of 213 [31.0%] vs 80 of 210 [38.1%]; relative risk, 0.81; 95% CI, 0.62 -1.06) and 60-day (82 of 211 [38.9%] vs 95 of 210 [45.2%]; relative risk 0.86; 95% CI, 0.68-1.08) mortality rates were lower in the albumin groups; however, neither reached statistical significance. The ICU (albumin vs control: 68 of 210 [32.4%] vs 71 of 204 [34.8%]; relative risk, 0.93; 95% CI, 0.70-1.23) and hospital (albumin vs control: 82 of 203 [40.4%] vs 90 of 201 [44.8%]; relative risk, 0.90; 95% CI, 0.71-1.14) mortality rates were similar in the 2 groups. There were no significant differences between groups in median (IQR) length of stay in the ICU (albumin vs control: 13 [7-22] days [95% CI, 8-14 days] vs 12 [7-29] days [95% CI, 10-19 days]) or hospital (albumin vs control: 24 [15-42] days [95% CI, 21-29 days] vs 27 [14-45] days [95% CI, 23-35 days]) or in the median (IQR) number of days in the ICU without vasopressors (albumin vs control: 2 [0-7] days [95% CI, 2-4 days] vs 2 [0-6] days [95% CI, 1-2 days]) or mechanical ventilatory assistance (albumin vs control: 4 [1-7] days [95% CI, 3-4 days] vs 3 [0-7] days [95% CI, 2-4 days]) (Table 2). Fluid intake and output were similar in the 2 groups for the 28 days after randomization (eFigures 3 and 4 in Supplement 2), as were hemodynamic parameters and SOFA scores.

**Table 2.** Patient Outcomes

Outcome	No./total No. (%)^a^	
	Albumin group	Control group
Primary outcome		
Death at 90 d	91/210 (43.3)	96/209 (45.9)
Secondary outcomes		
Death		
At 28 d	66/213 (31.0)	80/210 (38.1)
At 60 d	82/211 (38.9)	95/210 (45.2)
ICU	68/210 (32.4)	71/204 (34.8)
In-hospital	82/203 (40.4)	90/201 (44.8)
Length of stay in ICU, median (IQR) [95% CI], d	13 (7 to 22) [8 to 14]	12 (7 to 29) [10 to 19]
In-hospital length of stay, median (IQR) [95% CI], d	24 (15 to 42) [21 to 29]	27 (14 to 45) [23 to 35]
Ventilator-free days until ICU discharge or death, median (IQR) [95% CI]	4 (1 to 7) [3 to 4]	3 (0 to 7) [2 to 4]
Vasopressor-free days until ICU discharge or death, median (IQR) [95% CI]	2 (0 to 7) [2 to 4]	2 (0 to 6) [1 to 2]
Fluid administration, median (IQR) [95% CI], mL		
0-6 h	1531 (1048 to 2327) [1408 to 1699]	1598 (850 to 2510) [1373 to 1836]
24 h	3796 (2764 to 5317) [3477 to 4069]	4314 (3046 to 6677) [4102 to 4668]
48 h	7095 (5494 to 9516) [6891 to 7644]	7857 (5940 to 11 272) [7464 to 8582]
96 h	14 275 (11 145 to 17 514) [12 993 to 15 038]	15 263 (11 386 to 19 187) [13 912 to 16 711]
7 d	24 754 (21 527 to 30 651) [23 206 to 26 008]	26 188 (19 098 to 32 354) [24 106 to 28 497]
Amount per day in the ICU for a maximum of 28 d	3200 (2502 to 4149) [3093 to 3415]	3693 (2540 to 4443) [3410 to 3881]
Cumulative fluid balance, median (IQR) [95% CI], mL		
0-6 h	1062 (580 to 1884) [929 to 1263]	910 (282 to 1986) [718 to 1218]
24 h	1365 (220 to 2877) [1052 to 1714]	1666 (453 to 4198) [1419 to 2186]
48 h	1620 (to 229 to 3959) [1049 to 2250]	2414 (218 to 5618) [1920 to 2942]
96 h	851 (−1973 to 4923) [154 to 1858]	2136 (−907 to 6418) [1252 to 3544]
7 d	−151 (−3911 to 6774) [−1537 to 1849]	2230 (−2884 to 8348) [242 to 4234]
Amount per day in the ICU for a maximum of 28 d	234 (−491 to 1290) [90 to 445]	355 (−402 to 1576) [161 to 518]
New organ failure in the ICU		
1	68 (34.2)	52 (26.3)
2	19 (9.5)	14 (7.1)
3	2 (1.0)	7 (3.5)
4	0	1 (0.5)
SOFA scores in the ICU		
Maximum, median (IQR) [95% CI]	12 (10-15) [12-13]	12 (10-15) [11-13]
Mean, median (IQR) [95% CI]	9 (8.6-9.1) [8.6-9.1]	8.5 (8.1-8.9) [8.3-8.7]

Abbreviations: ICU, intensive care unit; SOFA, Sequential Organ Failure Assessment.

^a^ Unless otherwise indicated.

Results of the per-protocol analysis were in agreement with the main analysis: 90-day survival was 56.5% (118 of 209, with 11 missing values) for the albumin group and 58.7% (64 of 109, with 3 missing values) for the control group excluding all patients receiving any albumin (*P* = .52) and 53.8% (107 of 199, with 9 missing values) for the control group without omitting patients receiving albumin (*P* = .67). In the subgroup analyses, no significant differences in 90-day mortality were seen between treatment groups according to baseline values of lactate, SOFA, APACHE II, or SAPS II scores (eTable 8 in Supplement 2).

### Adverse Events 

A total of 267 AEs occurred in 121 patients (54.5%) in the albumin group and 202 events in 105 patients (48.1%) in the control group. The frequencies and severities of these events (eTable 9 in Supplement 2) and in sepsis-related events (eTable 10 in Supplement 2) were similar between study groups. Overall, the frequencies of AEs, whether sepsis related or not, were similar between the albumin and control groups.

## Discussion

Administration of albumin, aiming to maintain serum albumin concentrations of 3.0 g/dL or higher did not improve 90-day survival in patients with septic shock compared with treatment without albumin. The frequency of AEs was similar between groups, confirming the absence of any potential harm of albumin therapy, as implemented by the study protocol.

Despite the potentially beneficial properties of albumin, current evidence and guidelines do not support its routine use in patients with sepsis. Although previous post hoc or subgroup analyses from 2 randomized controlled studies^8,10^ suggested a possible benefit in patients with sepsis, we were unable to replicate this in a septic shock population. The routine use of albumin in patients with septic shock may thus not be justified, especially in view of the relatively high cost of this therapy.

The lack of a positive effect of albumin therapy on the primary end point may be attributed to several factors. First, the premature termination made the study underpowered to identify significant differences between groups. The COVID-19 pandemic strongly impacted health care, in particular intensive care. The ICUs’ capacities reached a critical level with the treatment of critically ill patients with COVID-19, including those contributing to our study. In addition, research activities were prioritized to COVID-19–related trials, and contact restrictions limited our ability to promote the study and perform timely monitoring activities. Second, most patients included in the study were postoperative, in whom outcomes may be significantly influenced by surgical factors beyond sepsis management. Third, although serum albumin levels in the treatment group were higher than in the control group, more than half the patients did not reach the target of 3.0 g/dL during their ICU stay. However, this proportion was similar to the ALBIOS study in which an outcome benefit was reported in the cohort with septic shock.^10^ Fourth, the antioxidant capacity of albumin may vary by the type of commercial albumin preparation^23^ and by duration of exposure to oxidative stress.^24^ Whether achieving a higher target level of albumin or only using nonoxidized albumin would prove beneficial requires future study. Fifth, we have used albumin 20% to achieve target serum albumin levels, limiting the comparability with the results of the SAFE study, in which albumin 4% with different pharmacokinetic and pharmacodynamic properties was used.^8^

### Limitations

This study has some limitations. Due to premature termination, the study was underpowered to exclude a potential beneficial of albumin replacement. Although we have followed a more liberal albumin administration protocol compared with the ALBIOS study (0-80 vs 0-60 g/d, respectively), we were not able to achieve a higher proportion of patients with serum albumin concentrations greater than 3.0 g/dL. Apparently, higher doses of albumin would have been needed to achieve our target serum level due to high severity of illness and pronounced dilutional effect of high fluid resuscitative volume in the context of septic shock. Another limitation of our study concerns the allowed use of albumin in the control group. As current guidelines recommend albumin administration when excessive crystalloid fluid is required to achieve hemodynamic stabilization, it would have been unethical to ban its use completely. A similar observation was also reported for the ALBIOS control arm.^10^ This use is, however, required only in the early phase on admission, whereas the current trial aimed at albumin substitution beyond hemodynamic stabilization. Although our results do not support the routine use of albumin therapy in patients with septic shock, it confirms its safety profile because the frequency of AEs, whether sepsis related or not, was similar between the albumin and control groups.

## Conclusions

In this randomized clinical trial study of patients with septic shock, albumin administration aiming to maintain serum albumin concentrations greater than 3.0 g/dL was safe but did not improve 90-day survival in these patients. Uncertainty remains due to premature termination of the study. Uncertainty remains due to premature termination of the study, and additional studies are recommended.

## Notes

Supplement 1.
Trial Protocol

Supplement 2. **eTable 1.** Sepsis-related clinical events, possible adverse effects of the trial drug, and classification of severity of adverse events (AEs) **eTable 2.** Patient inclusion according to site **eTable 3.** Types of infection in the two study groups at randomization **eTable 4.** Mode of acquisition of infection in the two study groups **eTable 5**. Albumin levels of patients by study time frame **eTable 6.** Protocol deviations in the two study groups **eTable 7.** Physiologic and oxygenation parameters, fluid administration, vasopressor therapy, and procedures in the two groups 6 hours after randomization **eTable 8.** Mortality rates in the two study groups according to baseline lactate, baseline SOFA, SAPS II, and APACHE II **eTable 9.** Frequency of AEs according in the two study groups **eTable 10.** Frequency of sepsis-related events in the two study groups **eFigure 1.** Study flow chart of interventions **eFigure 2.** Box plot showing mean arterial pressure in the two groups measured at screening, 6 hours after randomization, and then daily for 28 days in the ICU **eFigure 3.** Box plot showing the mean daily fluid intake in the two groups at screening, 6 hours after randomization, and then daily for 28 days in the ICU **eFigure 4.** Box plot showing the mean daily fluid output (excluding perspiration) in the two groups at screening, 6 hours after randomization, and then daily for 28 days in the ICU

Supplement 3.
The SepNet Critical Care Trials Group and ARISS investigators

Supplement 4.
Data Sharing Statement
