# Error in Methods

**DOI:** 10.1001/jamanetworkopen.2026.18973

**Published:** 2026-05-26

In the Original Investigation titled “Albumin Replacement Therapy in Septic Shock: A Randomized Clinical Trial,”^1^ published February 19, 2026, there was an error in the Methods. In the Patients subsection, the third inclusion criterion should have read as “(3) serum lactate concentration higher than 18 mg/dL” rather than “lower than.” This article has been corrected.^1^
