# Supplementary material for: Albumin Replacement Therapy in Septic Shock: A Randomized Clinical Trial
Source: JAMA Netw Open. 2026 Feb 19;9(2):e2559297. doi: 10.1001/jamanetworkopen.2025.59297 (PMC12921518; doi:10.1001/jamanetworkopen.2025.59297)
Supplement: Supplement 1. — Trial Protocol [file jamanetwopen-e2559297-s001.pdf]

Clinical trial Protocol according to the German Medicinal Products Act (AMG)

**Title of the trial**	Randomised controlled multicentre study of albumin replacement therapy in septic shock
**Trial Acronym**	ARISS-Study
**Trial code, ZKS Jena**	ZKSJ0112
**Sponsor's protocol code number**	ZKSJ0112_ARISS
**EudraCT number**	2018-001874-89
**Version/Version number**	Final 3.0
**Date**	18.07.2019
**Sponsor according to AMG**	Friedrich Schiller University Jena
**Sponsor's legal representative (SpB) and principal investigator (LKP)**	Prof. Dr. Dr. Yasser Sakr<br>Universitätsklinikum Jena<br>Klinik für Anästhesiologie und Intensivmedizin<br>Am Klinikum 1<br>07747 Jena<br>Phone: +49 (0) 3641 9323248<br>Fax: +49 (0) 3641 9323102<br>E-Mail: Yasser.Sakr@med.uni-jena.de
**Study statistician**	Dr. Ulrike Schumacher<br>Universitätsklinikum Jena<br>Zentrum für Klinische Studien<br>Postfach, 07740 Jena<br>Tel.: +49 (0) 3641 9396656<br>Fax: +49 (0) 3641 9399969<br>E-Mail: ulrike.schumacher@med.uni-jena.de

Confidentiality
The information in this study protocol is to be kept strictly confidential. It serves only to inform the investigators, their deputies, all medical and possibly further members of the trial group, other persons involved in the study, as well as the ethics committee and the authorities involved. The content of this protocol may not be disseminated orally or in writing to bystanders without the consent of the sponsor.

Table of Contents

1. General information 5

1.1 Overview of the clinical trial 5
1.2 Organisational structure – involved persons and institutions 9
1.3 Signatures 12
1.4 List of abbreviations 13
1.5 Overview (flowchart) of the clinical trial process 14
1.6 Schedule of the trial visits 15

2. Background to the clinical trial 17

2.1 Background and research questions 17
2.2 Benefit-risk assessment and justification of the project 18
2.3 Assessment of the generalisability of the expected study results 19

3. Objectives and endpoints of the clinical trial 20

3.1 Primary objective and primary endpoint 20
3.2 Secondary objectives and secondary endpoints 20

4. Trial design 21

4.1 Description of the clinical trial 21
4.2 Feasibility of recruitment 21
4.3 Trial duration 21

5. Participation in the clinical trial 22

5.1 Selection of participants 22
5.1.1 Explanation of sex distribution of the study population 22
5.1.2 Inclusion criteria 22
5.1.3 Exclusion criteria 22
5.1.4 Measures to prevent participation in further studies 23
5.2 Informed consent 23
5.2.1 Patients able to consent 23
5.2.2 Patients who are unable to consent 24
5.2.3 Patients who regain their ability to consent 25
5.2.4 Information in the case of changes in course of the trial 26
5.3 End of participation in the clinical trial 26
5.4 Prerequisites for participation in the trial 26

6. Trial drug 27

6.1 Labelling of the trial drug 27
6.2 Drug Accountability 27
6.2.1 Transport by the manufacturer 27
6.2.2 Storage and delivery to the trial centres or their pharmacies 27
6.2.3 Storage and provision by the pharmacy of the trial centres 27
6.2.4 Storage, use and tracing by the trial centre 27
6.3 Administration of the trial drug (only in the albumin group) 28
6.3.1 Start dose 28
6.3.2 Subsequent administration of the trial drug 28
6.3.3 Scheduled discontinuation of the trial drug 28
6.4 Premature discontinuation of the trial drug 29
6.5 Discontinuation of the trial interventions- discontinuation due to a medical reason 29
6.5.1 Compliance 30
6.6 Instructions for the control group without albumin 30
6.7 Permitted concomitant medication, concomitant therapies 30
6.8 Further treatment of participants after the end of participation 30

7. Start, course and end of the clinical trial/study visits 32

7.1 Screening and 24-h period before randomisation 32
7.2 Time of randomisation 33

7.3 Time period until 2 h after randomisation 33
7.4 Trial visit at 6 h after randomisation 34
7.5 Trial visits on trial days 1 to 28 after randomisation 34
7.5.1 Data collection at hospital discharge or at the end of the study 34
7.5.2 Data collection at trial days 28, 60, and 90 after randomisation 35
7.6 Premature end of the participation of a patient in the clinical trial 35
7.7 End of the clinical trial 36
7.7.1 Scheduled end 36
7.7.2 Premature termination of the trial in a trial centre 36
7.7.3 Premature termination of the entire clinical trial 37

8. Safety and adverse events 38

8.1 Assessment of the safety of the trial drug 38
8.2 Adverse events 38
8.3 Duration of recording adverse events 39
8.4 Documentation of adverse events 39
8.4.1 Intensity of an adverse event 40
8.4.2 Causality – relationship between the adverse event and the trial drug 40
8.4.3 Procedures in case of adverse events 41
8.4.4 Outcome of an adverse event 41
8.5 Serious adverse event 41
8.6 Reporting serious adverse events 41
8.6.1 Obligations of the investigator/deputy 42
8.6.2 Obligations of the SpB 42
8.7 Preexisting diseases 42
8.8 Pregnancy 42
8.9 Data and safety monitoring board - SMC 43

9. Documentation and data management 44

9.1 List of responsibilities 44
9.2 Documentation of screening 44
9.3 Patient identification list 44
9.4 Investigator site file (ISF) 44
9.5 Case report form (CRF) 44
9.6 Data handling and data management 45
9.7 Data retention, archiving of documents 45
9.7.1 Data retention obligation of the SpB 45
9.7.2 Data retention obligation of the trial centre 45
9.8 Privacy and privacy statement 46
9.8.1 Privacy statement 46

10. Quality assurance 47

10.1 Standardisation and validation 47
10.2 Monitoring 47
10.3 Audits and inspections 47

11. Statistical methods and determination of sample size 48

11.1 Sample size 48
11.2 Randomisation 48
11.3 Statistical methods 49
11.3.1 Definition of the analysis populations 49
11.3.2 Data analysis 49
11.3.3 Subgroup analyses 50
11.3.4 Interim analysis 50
11.4 Presentation of results 51

12. Regulatory and administrative aspects 52

12.1 Regulations 52
12.2 Ethic committees 52
12.3 Competent authorities 52

12.3.1 Federal authority 52
12.3.2 State authorities 52
12.4 Subsequent changes in the trial protocol - amendments 52
12.5 Registration 53
12.6 Insurance 53
12.7 Funding 53
12.8 Reports of the study results 53
12.9 Publications 53

13. Literature 55

14. Appendix 57

14.1 SOFA Score 57
14.2 Apache II Score 59
14.3 New Simplified Acute Physiology Score (SAPS II) 60
14.4 Conversion tables 61

1. General information

1.1 Overview of the clinical trial

**Title of the trial**	Randomised controlled multicentre study of albumin replacement therapy in septic shock
**Trial Acronym**	ARISS-Study
**Sponsor according to AMG**	Friedrich Schiller University Jena
**Sponsor's legal representative (SpB) and principal investigator (LKP)**	Prof. Dr. Yasser Sakr<br>Universitätsklinikum Jena<br>Klinik für Anästhesiologie und Intensivmedizin<br>Am Klinikum1<br>07747 Jena<br>Phone: +49 (0) 3641 9323248<br>Fax: +49 (0) 3641 9323102<br>E-Mail: Yasser.Sakr@med.uni-jena.de
**EudraCT Number**	2018-001874-89
**Study population**	Female and male patients ≥ 18 years treated in the intensive care unit (ICU) within 24 hours of septic shock diagnosis
**Study phase**	III b
**Trial design**	Prospective, multicentre, randomised, controlled, parallel-grouped, open-label, interventional clinical trial according to the German Medicinal Products Act
**Aim of the trial**	Primary target:<br>To investigate whether albumin administration and maintenance of serum albumin concentrations of at least 30 g/l in the ICU for up to 28 days after the onset of septic shock, will reduce total 90-day mortality compared to volume replacement therapy without albumin<br>Secondary targets:<br>- To investigate whether albumin administration and maintenance of serum albumin concentrations of at least 30 g/l in the ICU for up to 28 days after the onset of septic shock would influence ICU, hospital, 28- and 60-day mortality rates, rates of organ dysfunction/failure as assessed by the SOFA score, ICU/hospital lengths of stay, ventilator and vasopressor-free days.<br>- Cost-benefit analysis of volume replacement therapy from ICU admission until maximum day 28
**Primary outcome (primary end-point)<br>Secondary outcome (secondary end-point)**	Primary outcome: 90-day all-cause mortality<br><br>Secondary outcomes:<br>- 28- and 60-day mortality, mortality rates in the ICU and during hospitalization, organ dysfunction/failure as assessed by the SOFA score, ICU and hospital length of stay, ventilator and vasopressor free days.

	- Cost-benefit parameters of volume replacement therapy (albumin, other colloids and crystalloids)<br>- Total amount of fluid of fluid administration and total fluid balance in the ICU.<br>- Safety related endpoints: Onset of AEs and SAEs, in particular anaphylactic shock, hypervolaemia, pulmonary oedema and sepsis-related clinical events
**Sample size**	Assuming a 90-day mortality of 50% in the control group without albumin and 42% in the albumin group, ***1412*** patients need to be evaluated (power: 80%, Chi²Test alpha = 0.05). Taking into account a dropout rate of 15%, ***1662*** patients should be randomised in the trial (831 in each arm).
**Inclusion criteria**	- The presence of septic shock meeting all of the following criteria (1):<br>o Clinically possible or probable or microbiologically confirmed infection according to the definitions of the International-Sepsis-Forums (ISF) (2)<br>o Despite adequate volume therapy, vasopressors are required to maintain mean arterial pressure (MAP) ≥ 65 mm Hg for at least 1 hour<br>o Serum lactate level > 2 mmol/l (18 mg/dl) despite adequate volume therapy<br>- Start of septic shock less than 24 hours prior to inclusion, so that the start dose of the trial drug in the albumin group will be possible within 6-24 hours after the start of the septic shock<br>- Age: ≥ 18 years<br>- Written informed consent of the patient or his/her legal representative or confirmation of the urgency of participation in the clinical trial and possible benefit to the patient by an independent consultant or the implementation of other established procedures according to the local regulations of the contributing centre to include patients who are unable to provide informed consent in whom subsequent consent may be obtained retrospectively.<br>- Patients of childbearing age: negative pregnancy test
**Exclusion criteria**	- Moribund conditions with life expectancy less than 28 days because of comorbid conditions or advanced malignant disease and palliative situations with life expectancy less than 6 months<br>- Presence of an "end of life" decision prior to obtaining informed consent: "Do Not Resuscitate (DNR)" and "Withhold/Withdraw Life-Sustaining measures"<br>- Previous participation in this study<br>- Participation in another interventional clinical trial within the past 3 months<br>- Shock states that can be explained by other reasons, e.g. cardiogenic, anaphylactic, and neurogenic shock

	- History of hypersensitivity to albumin or any other component of the trial drug, e.g., B., sodium caprylate, sodium N-acetyltryptophanate<br>- Diseases in which albumin administration may be deleterious, e.g., decompensated heart failure or traumatic brain injury<br>- Clinical conditions where albumin administration is indicated, e.g., hepatorenal syndrome, nephrosis, burns, intestinal malabsorption syndrome<br>- Lactation
**Trial drug administered in the intervention group (albumin group)**	- Active substance: human albumin 200g/l<br>- Product: Albutein® 200 g/l solution for infusion<br>or<br>Plasbumin® 20, solution for infusion<br>Both drugs are manifactured according to the same procedure and considered to be interchangeable.<br>- Manufacturer: Instituto Grifols, S.A., Barcelona, Spain<br>- License holder: Grifols Deutschland GmbH, Frankfurt,<br>- Approval number: 10577a / 97<br>- Route of application: intravenous administration<br>- Dosage:<br>Starting dose: 60 g human albumin 20% (200 g/l, infusion solution) over 2-3 h<br>Dose adjustment from study day 1 after randomisation depending on serum albumin concentration:<br>≥ 30 g/l: no administration<br>≥ 25 g/l and <30 g/l: 40 g over 1-2 h<br>≥ 20 g/l and <25 g/l: 60 g over 2-3 h<br><20 g/l: 80 g over 3-4 h<br>- Duration of use: maximum 28 days after randomisation
**Study-specific procedures**	- Woman of childbearing age: pregnancy test<br>- If necessary, blood collection until 2 h after randomisation (determination of serum albumin concentration)<br>- Albumin group only: administration of the starting dose and dose adjustment depending on the serum albumin concentration<br>- Daily determination of serum albumin concentration between 4:00 - 9:00 in the morning, if this cannot be determined from blood taken during routine blood sampling at the respective trial centre.<br>- Accompanying scientific project: Blood sampling during routine withdrawals at 4 different time points.
**Trial duration**	**Patient-related duration of the trial**<br>- maximum duration of treatment (administration of the trial drug) in the albumin group: 28 days<br>- Data collection: ends on study day 90 after randomisation

	**Trial-related duration of the trial**<br>- Planned recruitment period: approx. 36 months<br>- End of clinical trial: Date on which all data in the eCRF have been recorded and monitored
**Number of contributing centres**	n = 40 - 50 centres
**Statistical methods**	- Descriptive analysis of all collected data<br>- Primary end-point, 90-day mortality: generalised mixed model with random effects "centre" and "patient in centre" and fixed effects "SAPS II", "SOFA", "baseline serum albumin concentration" and "treatment group"<br>- Secondary outcomes: descriptive analysis<br>- Subgroup analyses according to baseline lactate, baseline quartiles of SOFA, SAPS II and APACHE II<br>- AEs, SAEs and sepsis-related clinical events: frequency
**Financial support**	- Deutsche Forschungsgemeinschaft (DFG), Förderkennzeichen: DFG SA 2167/3-1, Projektnummer: 328809707<br>- Instituto GRIFOLS S.A., Barcelona, Spanien<br>- Klinik für Anästhesiologie und Intensivtherapie, Universitätsklinikum Jena

1.2 Organisational structure – *involved persons and institutions*

Function/Qualification	Name/contact person, facility	Contact details
**Sponsor according to AMG**	Friedrich Schiller University Jena	
**Sponsor's legal representative (SpB) and principal investigator (LKP)**	Prof. Dr. Yasser Sakr<br>Universitätsklinikum Jena,<br>Klinik für Anästhesiologie und Intensivmedizin	Am Klinikum 1, 07747 Jena<br>Phone: +49 (0) 3641 9323248<br>Fax: +49 (0) 3641 9323102<br>E-Mail: Yasser.Sakr@med.uni-jena.de
**Repesentative of the LKP**	Prof. Dr. Michael Bauer<br>Universitätsklinikum Jena,<br>Klinik für Anästhesiologie und Intensivmedizin	Am Klinikum 1, 07747 Jena<br>Phone: +49 (0) 3641 9323101<br>Fax: +49 (0) 3641 9323102<br>E-Mail: Michael.Bauer@med.uni-jena.de
**Study statistician**	Dr. Ulrike Schumacher<br>Universitätsklinikum Jena,<br>Zentrum für Klinische Studien	Postfach, 07740 Jena<br>Phone: +49 (0) 3641 9396656<br>Fax: +49 (0) 3641 9399969<br>E-Mail:<br>ulrike.schumacher@med.uni-jena.de
**Protocol committee**	Prof. Dr. Yasser Sakr<br>Prof. Dr. Michael Bauer	Universitätsklinikum Jena
	Dr. Ulrike Schumacher<br>Dr. Maria Breternitz<br>Dr. Sabine Barta	Universitätsklinikum Jena<br>Zentrum für Klinische Studien
	Prof. Dr. Michael Hartmann<br>PD Dr. Michael Kiehntopf	Universitätsklinikum Jena
	Prof. Dr. Luciano Gattinoni<br>Prof. Dr. Michael Quintel	Universitätsklinikum Göttingen
**Contact person for the trial drug**	Cynthia Henning<br>Investigator Sponsored Research Program<br>Grifols Shared Services NA, Inc.<br>Global Bioscience Scientific & Medical Affairs	Avinguda de la Generalitat 152 - Edificio 2, Plta 1<br>08174- Sant Cugat del Vallès - Barcelona - SPAIN<br>Phone: +323 227-7602<br>E-Mail:<br>cynthia.henning@grifols.com
	Instituto Grifols S.A.<br>Francisco M. Mota Villaplana, Pharm D, MSc<br>Senior Manager Medical Affairs - EU/ROW<br>Intensive Care	Avinguda de la Generalitat 152 - Edificio 2, Plta 1<br>08174- Sant Cugat del Vallès - Barcelona - SPAIN<br>Phone: 93-571-07-19<br>Mobile: 670-92-44-20<br>E-Mail: francisco.mota@grifols.com
**Project management**	Universitätsklinikum Jena,<br>Zentrum für Klinische	Postfach, 07740 Jena<br>Fax: +49 (0) 3641 9399969<br>E-Mail: ZKS-

Function/Qualification	Name/contact person, facility	Contact details
	Studien	Projektmanagement@med.uni-jena.de
**Data management**	Universitätsklinikum Jena, Zentrum für Klinische Studien	Postfach, 07740 Jena<br>Fax: +49 (0) 3641 9399984<br>E-Mail: zks-support@med.uni-jena.de
**Monitoring**	Universitätsklinikum Jena, Zentrum für Klinische Studien	Postfach, 07740 Jena<br>Fax: +49 (0) 3641 9399969
**Pharmacovigilance (safety management)**	Universitätsklinikum Jena, Zentrum für Klinische Studien	Postfach, 07740 Jena<br>Fax: +49 (0) 3641 9399946<br>E-Mail: ZKS_SAE_Management@med.uni-jena.de
**Data and safety monitoring board -SMC**	See corresponding manual	
**Laboratory (s)** for the determination of serum albumin and arterial blood lactate concentrations	local laboratories of the respective trial centres	see respective trial centre documents
**Central pharmacy** for storage and dispatch of the trial drug to trial centre pharmacies	Prof. Dr. Michael Hartmann, Apotheke, Universitätsklinikum Jena	Am Klinikum 1, 07747 Jena<br>Phone: +49 (0) 9325401<br>Fax: +49 (0) 9325402<br>E-Mail: Michael.Hartmann@ med.uni-jena.de
**Pharmacies** for the provision of the trial drug to the trial centres	local pharmacy of the respective trial centre	see respective trial centre documents
**Pharmacoeconomic analysis**	Prof. Dr. Michael Hartmann, Apotheke, Universitätsklinikum Jena	Am Klinikum 1, 07747 Jena<br>Phone: +49 (0) 3641 9325401<br>Fax: +49 (0) 3641 9325402<br>E-Mail: Michael.Hartmann@ med.uni-jena.de
**Responsible higher federal authority (BOB)**	Paul-Ehrlich-Institut (PEI), Bundesinstitut für Impfstoffe und biomedizinische Arzneimittel	Paul-Ehrlich-Str. 51-59<br>63225 Langen<br>Phone: +49 (0)6103 77 0
**Leading ethics committee**	Ethik-Kommission der Friedrich-Schiller-Universität Jena	Universitätsklinikum Jena<br>Postfach, 07740 Jena<br>Phone: +49 (0) 3641 933770<br>Fax: +49 (0) 3641 933771<br>E-Mail: ethikkommission@med.uni-jena.de
**Funding of the study, material and other support**	Deutsche Forschungsgemeinschaft e.V.	Kennedyallee 40<br>53175 Bonn<br>Phone: +49 (0) 228 8851<br>Fax: +49 (0) 228 8852777<br>E-Mail: postmaster@dfg.de

Function/Qualification	Name/contact person, facility	Contact details
	Francisco M. Mota Villaplana, Pharm D, MSc<br>Senior Manager Medical Affairs – EU/ROW Intensive Care<br>Instituto Grifols, S.A.	Avinguda de la Generalitat 152 – Edificio 2, Plta 1<br>08174- Sant Cugat del Vallès – Barcelona – SPAIN<br>Phone: 93-571-07-19<br>Mobile: 670-92-44-20<br>E-Mail: francisco.mota@grifols.com
	Klinik für Anästhesiologie und Intensivmedizin, Universitätsklinikum Jena	Am Klinikum 1, 07747 Jena<br>Phone: +49 (0) 3641 9323101<br>Fax: +49 (0) 3641 9323102<br>E-Mail: Michael.Bauer@med.uni-jena.de

1.3 Signatures

The following persons confirm the accuracy of the contents of this trial protocol and confirm this with their signature.

Prof. Dr. Yasser Sakr, **Sponsor's legal representative and principal investigator** Date

Dr. Ulrike Schumacher, **Statistician** Date

1.4 List of abbreviations

Abbreviation	Explanation
**AC**	Arterial catheter
**AE**	Adverse event
**AMG**	Arzneimittelgesetz (medicinal products act)
**APACHE**	Acute Physiology and Chronic Health Evaluation
**ARDS**	Acute respiratory distress syndrome
**BOB**	Bundesoberbehörde (Federal authority)
**CO**	Cardiac output
**CRF**	Case report form
**CVC**	Central venous catheter
**CVP**	Central venous pressure
**Da**	Dalton
**DAP**	Diastolic blood pressure
**DFG**	Deutsche Forschungsgemeinschaft
**DIC**	Disseminated intravascular coagulopathy
**dl**	Deciilitre
**DRKS**	Deutsches Register klinischer Studien (German Registry of clinical trials)
**ECMO**	Extracorporeal membrane oxygenation
**EK**	Ethics Committee
**FiO2**	Fraction of inspired oxygen
**g**	Gram
**GCLP**	Good Clinical Laboratory Practice
**GCP**	Good Clinical Practice
**GCP-V**	GCP-Regulation
**h**	Hour
**IBBJ**	Integrated Biobank Jena
**ICU**	Intensive care unit
**ISF**	Investigator Site File
**ITN**	Intubation
**l**	Litre
**LKP**	Principal investigator
**MAP**	Mean arterial blood pressure
**mg**	Milligram
**mm Hg**	Millimetre mercury
**mmol**	Millimole
**NYHA IV**	New York Heart Association Class IV
**paCO2**	Partial pressure of carbon dioxide
**paO2**	Partial pressure of oxygen
**PEI**	Paul-Ehrlich-Institut, Bundesinstitut für Impfstoffe und biomedizinische Arzneimittel
**pH**	Negative logarithm of the hydrogen ion concentration
**RRT**	Renal replacement therapy
**SAP**	Systolic arterial blood pressure
**SAPS**	Simplified Acute Physiology Score
**SAE**	Serious adverse event
**ScO2**	Central venous oxygen saturation
**SMC**	Safety Monitoring Committee
**SO2**	Arterial oxygen saturation
**SOFA**	Sequential Organ Failure Assessment
**SpB**	Sponsor’s legal representative
**SUSAR**	Suspected unexpected serious adverse reaction
**SvO2**	Central venous oxygen saturation
**TMF**	Trial Master File
**ZKS**	Zentrum für klinische Studien (Centre for clinical studies)

1.5 Overview (flowchart) of the clinical trial process

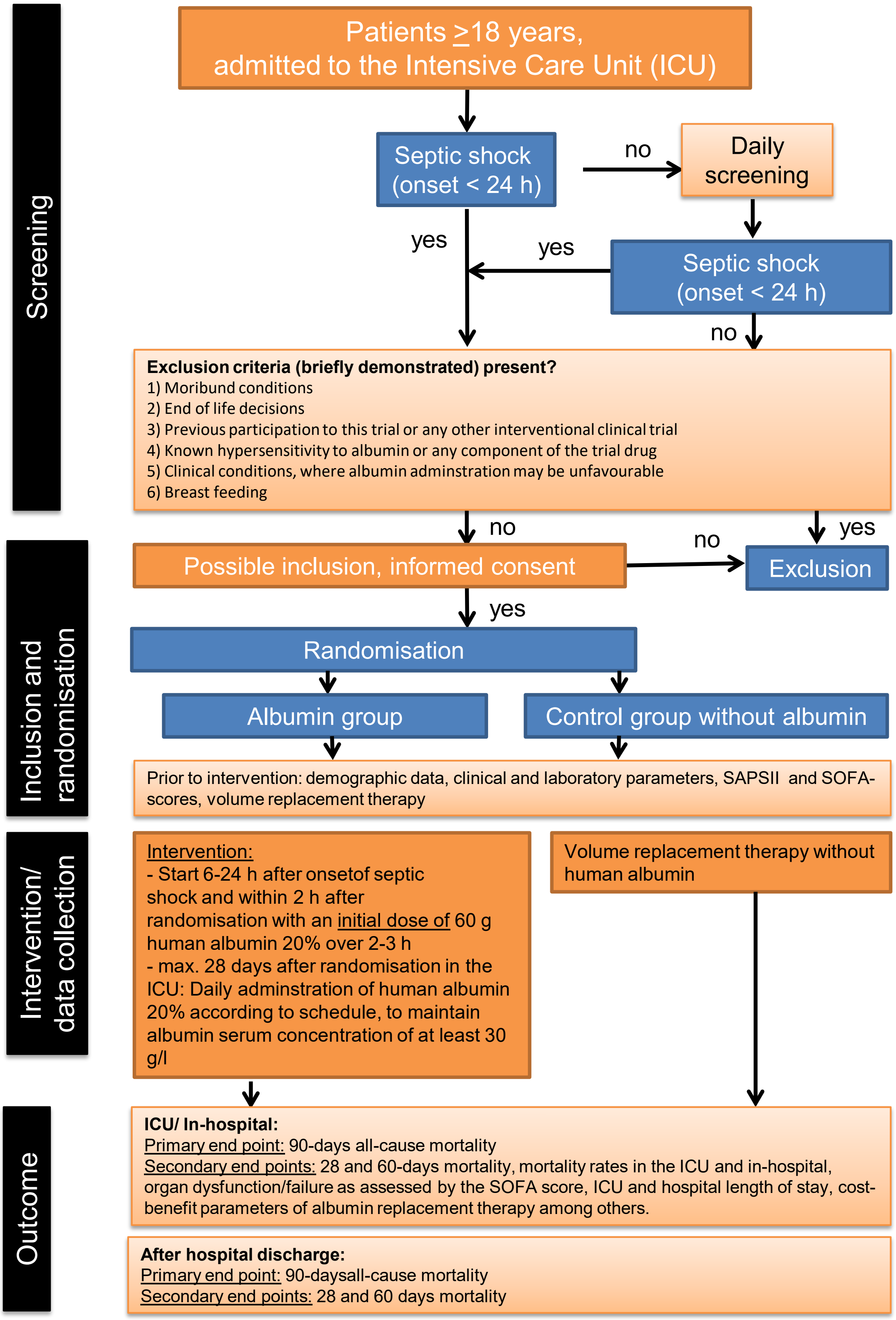

1.6 Schedule of the trial visits

	**Screening/ Baseline**		**maximal treatment duration with human albumin 20%** (200 g/l, Infusionslösung) **in the ICU**			**Data collection at discharge from the hospital or at the end of the tria**	**Data collection (if necessary by phone)**
Procedure, Event	24 h period before Randomisation	Randomisation (max. 2h after informed consent)	**until 2 h after** Randomisation and 6-24h after the onset of septic shock	6 h after Randomisation	Study day 1 until max. 28 days after randomisation		**Day 28, 60 and 90**
Checking inclusion and exclusion criteria	X						
Written informed consent	X						
Pregnancy test (female patients in childbearing age)[1]	X						
Collection of basic data[2]	X						
Documentation of primary and secondary admission diagnoses	X						
Documentation of comorbid conditions	X						
Documentation of data on infection, microbiology and anti-infective therapy (continuous collection of data)	←	—	—	—	→		
Collection of data on SAPS II-/APACHE II-Scores	X						
Collection of data for calculation of SOFA-Scores	X				X		
Determination of serum albumin levels			X[3]		X		
**Administration of the trial drug in the albumin group**			X		X, Dose adjustment according to serum albumin levels		
Documentation of albumin adminstration (continuous)			←	—	→		
Detection of clinical parameters [4]	X	X		X	X		
Documentation of concomitant medications	X[5]	X[5]		X[6]	X		
Collection of the results of routine laboratory parameters [7]		X[8]		X	X		
Documentation of procedures in the ICU [9]	X	X		X	X		
Collection of AEs, SAE, sepsis-related clinical events [10]		←	—	—	→	(X)	(X)
Collection of data on the vital status [11]				X	X	X	X
Collection of data on ICU length of stay						X	
Collection of data on hospital length of stay						X	

1 After obtaining informed consent

[2] Basic data: sex, age, weight, height, time of hospital admission, time of ICU admission, type of admission, referring facility prior to ICU admission, etc. from the 24-hour period before randomisation, (data from ICU or normal ward)
[3] Albumin group: Determination of serum albumin concentration before administration of the starting dose of the trial drug
[4] Body temperature, respiratory rate and haemodynamic parameters (SAP, MAP, DAP, CVP, CO)
[5] Catecholamines, inotropic agents, diuretics, volume replacement therapy, including blood transfusion, adjunctive sepsis therapy
[6] Recording of concomitant medication with catecholamines and inotropes at the corresponding time point (+/- 1 hour); recording of concomitant medication with diuretics, volume therapeutics including transfusion therapy, adjunctive sepsis therapy) of the previous 6 h
[7] Haemoglobin, creatinine, bilirubin, C-reactive protein, procalcitonin, leucocytes, platelets, lactate, arterial blood gas analysis (PaCO2, PaO2, FiO2, SO2, pH, ScO2)
[8] 24-h time period prior to randomisation
[9] ITN, CVC, AC, RRT, mechanical ventilation, ECMO, haemodynamic monitoring
[10] If AEs or SAEs are still "ongoing" after the end of treatment with the trial drug, observation will continue until the end of data collection (day 90).
[11] Data on the vital status (alive/dead), if applicable date of death and cause of death, residence after discharge, if applicable, recording of "End of Life" decisions

2. Background to the clinical trial

2.1 Background and research questions

Sepsis is the 10th leading cause of death in the developed world and is the leading cause of death in the intensive care unit (ICU) (3). It represents a significant burden on the healthcare system. The cost of treating critically ill patients with sepsis in German ICUs is approximately € 4.97 billion a year (4). An analysis of administrative data from German hospitals showed a continuous increase in the prevalence of sepsis of 5.7% per year between 2007 and 2013 (5). Although the mortality rate in this analysis of 33815 patients with septic shock showed a slight decrease in the last 3 years of the observation period, the hospital mortality rate in 2013 was still 58.8% (5).

Sepsis-related morbidity has also been demonstrated in several epidemiological studies, in particular multi-organ failure and prolonged length of stay in the ICU with a corresponding increase in healthcare expenditure (6-8). Several sepsis-specific therapeutic approaches have been evaluated for efficacy and effectiveness in recent decades, but failed to produce the expected results (9-12).

Albumin is a natural protein with a molecular weight of about 65,000 Da, whose production requires 50% of the total energy expenditure of the liver (13). It is responsible for about 80% of the oncotic pressure in the plasma and thus has a key role in the distribution of extracellular fluid. In addition to its oncotic functions, albumin has a variety of other properties, including binding and transport of various endogenous molecules (13), anti-inflammatory (14) and anti-oxidative effects (15), and modulation of nitric oxide metabolism (16). In addition, albumin has a buffer function (17). These properties are particularly relevant in critically ill patients, especially in patients with sepsis. An analysis of the oxidation state of the albumin molecule in patients with septic shock showed that up to 30% of circulating serum albumin loses its antioxidant capacity due to irreversible oxidation (unpublished data). Thus, albumin administration in both the early and maintenance phases of severe sepsis (septic shock) could offer a survival benefit in these patients, primarily by increasing the amount of functional albumin available to exert its anti-inflammatory, anti-oxidative and NO-modulating properties.

In 1998, a Cochrane meta-analysis reported increased mortality associated with albumin administration in critically ill patients (18). However, when further clinical studies were included in another meta-analysis by Wilkes et al., the safety of albumin therapy was confirmed, but with no corresponding survival benefit (19). Interestingly, a later meta-analysis by Vincent et al. showed that the use of human albumin in critically ill patients could reduce morbidity (20). Significant improvement in organ function of critically ill patients was confirmed in a pilot study in which albumin therapy was administered with the aim of maintaining serum albumin concentrations greater than 30 g/l (21). Based on the contradictory literature cited earlier, a large randomised, prospective, double-blind study was performed in 7,000 critically ill patients (SAFE study) (22). In this study, the possible effect of volume replacement therapy with human albumin 4% on the outcome of these patients was compared to volume replacement therapy with only crystalloids. Although the survival rates in the two groups were similar, a post-hoc analysis of 1218 patients with severe sepsis showed decreased mortality in the albumin group compared to patients treated with 0.9% saline solution alone (23). Consequently, the AIFA, the Italian Medicines Agency, funded the randomised controlled ALBIOS study (ALBumin Italian Outcome Sepsis Study), which investigated the possible impact on outcome of albumin administration and maintenance of serum albumin concentrations to at least 30 g/l in 1810 patients with severe sepsis and septic shock (24). To investigate the possible influence of the timing of albumin administration on outcome, randomisation in the ALBIOS study was stratified after the start

of the albumin therapy (within 6 h or 6-24 h after the onset of severe sepsis). The study showed no outcome difference between the study groups. Nevertheless, the results of the study gave two interesting signals: firstly, there was a tendency for a potential survival benefit of albumin therapy in patients who started therapy 6-24 h after onset of sepsis compared to those who started it earlier (relative risk of 90-day mortality = 0.9; 95% confidence interval: 0.81-1.05, p = 0.2). Secondly, in the 1121 patients with septic shock, 90-day mortality was lower in the albumin group (564 patients) than in the non-albumin group (43.6 vs. 49%, p = 0.03). Additional post-hoc analyses in various study subgroups confirmed a potential survival benefit in patients with increased disease severity (according to arterial blood pressure, lactate levels or SvO2 on randomization) (unpublished data). Taken together, the current evidence suggests that albumin administration in patients with severe and advanced sepsis who have potential impairment of the protective effects of serum albumin may provide a survival benefit. However, no prospective, randomised trial has adequately studied this hypothesis in patients with septic shock.

The aim of the ARISS study is to investigate the effect of albumin administration and maintenance of a serum albumin concentration of at least 30 g/l for 28 days after onset of septic shock compared to volume replacement therapy without albumin on patient survival. The hypothesis is that albumin administration started within 6-24 hours after the onset of septic shock aimed at maintaining a serum albumin concentration of at least 30 g/l for 28 days after the onset of septic shock will reduce 90-day all-cause mortality in these patients compared to volume replacement therapy without albumin.

2.2 Benefit-risk assessment and justification of the project

Therapeutic approaches in patients with septic shock are controversial. The arguments cited in 2.1 suggest that albumin administration may improve the outcome of such patients. However, there are no prospective randomised studies that have adequately investigated the possible impact of albumin therapy on the outcome of patients with septic shock. Although the current international guidelines for the treatment of sepsis and septic shock support albumin administration in the acute as well as the maintenance phase of sepsis and septic shock, the evidence for such treatment is grade "2C" (25), which may not justify the higher cost of routine albumin administration in these patients. Moreover, there are currently no specific recommendations regarding the maintenance of higher serum albumin levels in patients with septic shock. Therefore, based on the current evidence, both restrictive and liberal albumin administration may be justified. The treatment of patients in the two groups of the ARISS study does not therefore represent a deviation from standard practice. Inclusion in the clinical trial, therefore, poses no additional risks in this respect compared to standard practice. In addition, various methodological aspects have been taken into account to further shift the risk-benefit profile in favour of benefit.

General treatment of patients with septic shock participating in the ARISS study will follow usual practice for patients with sepsis and septic shock and will be compliant with current guidelines for the treatment of such patients. Patients with a history of hypersensitivity to albumin or any other component of the trial product are excluded from participating in the study. Exclusion criteria also include disease situations in which albumin administration may be deleterious, as well as medical conditions in which the use of human albumin is indicated. This minimises the risk profile in the two groups.

Patients will be randomised into two groups. The trial product will be administered only in the albumin group for a maximum of 28 days and only while in the ICU. Patients in the albumin group will receive the starting dose of the trial product for the first time within 6-24 h after the onset of septic shock, based on a subgroup analysis of the ALBIOS study (24), which demonstrated a survival advantage in the intervention group compared to the control group.

Patients will receive a starting dose of 60 g of 20% human albumin over 2-3 hours within 6-24 h after the onset of septic shock. Crystalloids will be administered according to usual practice in this condition. The prescribed dose of albumin has been used in previous clinical trials and is considered to be safe (21-24). In addition, these studies showed a potential advantage of albumin administration in terms of morbidity (21) and mortality (24).

The control group without albumin will be treated according to common practice with crystalloids as the volume replacement therapy of first choice. Therapy with human albumin in this group may be clinically indicated (dosing as low as possible) if, for example, the serum albumin concentration decreases below 15 g/l or after assessment of the clinical need for albumin administration by the treating physicians (member of the trial group). This minimises the risk of hypoalbuminaemia. The treatment concept in this group does not deviate from standard practice and therefore does not represent an additional risk factor.

Serum albumin concentration will be determined once daily. No additional blood sampling is required for this determination, as the value can normally be determined from blood taken between 4:00 and 9:00 as part of routine blood sampling at the respective trial centre. These blood samples will be taken, if possible, from existing vascular catheters. Thus, the likelihood of study-related blood vessel puncture and associated risks is minimal.

Dose adjustment will follow a predetermined schedule with the aim of maintaining serum albumin concentration at least at 30 g/l. The serum albumin concentration represents a valid treatment target for the trial drug. It is routinely determined in critically ill patients receiving albumin. The target value of at least 30 g/l corresponds to the target value from previous studies that showed a potential advantage of albumin administration in terms of morbidity (21) and mortality (24) without any safety concerns. Taken together, the study intervention is justified, low-risk and clinically relevant.

The results of the clinical trial may influence the treatment of patients with septic shock. The expected improvement in patient survival may result in a reduction in the resources currently used in the treatment of these patients and in the socioeconomic burden of this disease. If the hypothesis cannot be confirmed, restrictive albumin administration will be justified and the costs of therapy can be significantly reduced. In both cases, the results of the clinical trial could reduce the socio-economic burden of the disease and will be of high clinical relevance.

2.3 Assessment of the generalisability of the expected study results

It is expected that the exclusion criteria will not exclude more than 10-20% of patients with septic shock from participating in the clinical trial. Therefore, high generalisability of the results is expected. The results of the clinical trial are expected to impact on everyday clinical practice and will have a direct impact on guidelines for the treatment of patients with septic shock. If the established hypothesis can be confirmed, then albumin administration will improve the outcome of patients with septic shock and justify the higher costs of such therapy.

3. Objectives and endpoints of the clinical trial

3.1 Primary objective and primary endpoint

The primary objective of the clinical trial is to investigate whether albumin administration and maintenance of serum albumin concentrations of at least 30 g/l in the ICU for up to 28 days after the onset of septic shock will reduce 90-day all-cause mortality, compared to volume replacement therapy without albumin.

The primary endpoint is 90-day all-cause mortality (EMA Guideline CHMP / EWP / 4713/03). Based on the subgroup analysis of the ALBIOS study (24), the potential benefit of albumin administration is most likely to be apparent 90 days after randomization. Based on experience with the previous SepNet study (26, 27), the risk of patients being discharged from hospital 90 days after randomisation, and thus unavailable for follow-up, will not significantly affect data collection at this time point.

3.2 Secondary objectives and secondary endpoints

The purpose of the clinical trial is to secondarily investigate whether albumin administration and the maintenance of serum albumin concentrations of at least 30 g/l for 28 days after the onset of septic shock will influence 28- and 60-day mortality, ICU and hospital mortality rates, organ dysfunction/failure as assessed by the SOFA score, ICU and hospital lengths of stay, as well as ventilator- and vasopressor-free days. A cost-benefit analysis of the volume replacement therapy for the ICU stay up to a maximum of day 28 is another objective of this clinical trial.

Secondary end-points therefore are

- 28- and 60-day mortality,
- ICU and hospital mortality,
- organ dysfunction/failure as assessed by the SOFA-Score: recorded daily up to 28 days in the ICU after randomisation in the study,
- ICU and hospital lengths of stay,
- ventilator- and vasopressor-free days,
- cost-benefit of volume replacement therapy: collection of data on the use of human albumin and other colloids and crystalloids,
- Total amount of fluid administration and total fluid balance, and
- safety-related parameters: occurrence of AEs and SAEs, especially anaphylactic shock, hypervolaemia, and pulmonary oedema.

4. Trial design

4.1 Description of the clinical trial

The ARISS study is a prospective, multicentre, randomised, controlled, parallel-grouped, open-label, interventional clinical trial (Phase IIIb) according to the German Medicines Act (AMG). Participants are male and female persons ≥ 18 years with septic shock.

4.2 Feasibility of recruitment

The SepNet Study Group is a network of physicians and scientists from around 50 clinics and universities throughout Germany. This interdisciplinary network brings together experts from various disciplines working in the field of clinical and experimental sepsis research. They have considerable experience and interest in clinical trials in sepsis patients. These clinical studies can be performed at the well-established SepNet regional centres and numerous associated centres with interdisciplinary, medical and surgical ICUs. Successful studies carried out by the SepNet study group in the past have proved the ability of this network to perform the ARISS study. Based on estimates of expected recruitment numbers provided by the requested trial sites, it is anticipated that the clinical trial will achieve the required number of cases within the planned recruitment period.

Evaluation of data from the Jena Sepsis Registry (BMBF-ID: 01 E0 1002 and DRKS number: DRKS00008342) was carried out. In this registry, prospective data were collected from all patients with severe sepsis who were admitted to the 4 ICUs of the Jena University Hospital (2 surgical, 1 medical, 1 neurological) between January 2011 and December 2015. A total of 1988 patients with severe sepsis (within 24 hours of the onset of sepsis) were identified, 1478 of whom had septic shock. Only 176 patients had at least one of the exclusion criteria for the ARISS study. Sixty-two patients died or relocated to another facility within 24 hours of the onset of the septic shock. A total of 1,236 patients, i.e., approximately 247 patients per year, would have been suitable for the ARISS clinical trial.

It is estimated that the clinical trial will require 40-50 trial sites to randomise 1,662 patients over 3 years; that is, about 13-14 patients per year per site. The ICUs that have confirmed participation in the ARISS study within the framework of the DFG application stated the possibility of including an average of 141 patients with septic shock. A recruitment rate of up to 21 patients per year per trial centre is, thus, expected. These figures show that the required recruitment figures can be achieved with an adequate safety margin. A drop-out rate of 15% of randomised patients is assumed, resulting in 11-12 patients per year per site and a total of 1412 patients from all sites to be be included in the final evaluation.

4.3 Trial duration

Approximately 36 months are planned for the recruitment of patients.

Depending on the patients, study durations will vary in length. The trial product will be administered for a maximum of 28 study days after randomisation in the ICU.

On day 90 after randomisation, data collection and thus participation in the study will end for the respective patient.

5. Participation in the clinical trial

5.1 Selection of participants

The target population for the clinical trial are male and female patients ≥ 18 years of age with septic shock treated in an ICU.
Screening will be performed daily in the respective trial centres to see whether eligible patients are being treated in the ICUs.
Patients ≥ 18 years of age with septic shock will be approached to obtain informed consent to participate in the study. No patient will be excluded from participation because of sex, race, ethnicity, or sexual preference.

5.1.1 Explanation of sex distribution of the study population

The clinical trial does not involve any sex-specific issues. Patients will be consecutively screened during routine clinical practice for eligibility to participate in the clinical trial and included accordingly. The trial intervention is not sex-dependent and sex-related differences will not be addressed in the trial.

5.1.2 Inclusion criteria

Patients who meet all the following inclusion criteria may be included in this clinical trial:

• Presence of septic shock that meets all the following criteria (1):
  - o Clinically possible, probable or microbiologically confirmed infection according to the definitions of the International-Sepsis-Forums (ISF) (2)
  - o Despite adequate volume therapy, vasopressors are required to maintain mean arterial pressure (MAP) ≥ 65 mm Hg for at least 1 hour
  - o Serum lactate concentration > 2 mmol/l (18 mg/dl) despite adequate volume therapy

• Onset of septic shock less than 24 hours prior to study inclusion, so that administration of the initial dose of the trial product is possible within 6-24 hours after the onset of septic shock in the albumin group
• Age: ≥ 18 years
• Written informed consent of the patient or his/her legal representative (guardian) or confirmation of the urgency of participation in the clinical trial and the possible benefit to the patient by an independent consultant or the implementation of other established procedures according to the local regulations of the contributing centre to include patients who are unable to provide informed consent in whom subsequent consent may be obtained retrospectively (for more details see chapter 5.2)
• Patient of childbearing age: negative pregnancy test

5.1.3 Exclusion criteria

Patients who meet any of the following exclusion criteria cannot be included in the trial:

• Moribund conditions with life expectancy less than 28 days due to secondary diseases or advanced malignant disease and palliative situations with life expectancy less than 6 months
• "End of life" decisions made before obtaining informed consent: "Do Not Resuscitate (DNR)" and "Withhold/Withdraw Life-Sustaining measures"
• Previous participation in the study
• Participation in another interventional clinical trial within the past 3 months

• Shock states that can be explained by other reasons, e.g. cardiogenic, anaphylactic, and neurogenic shock
• History of hypersensitivity to albumin or any other component of the trial product, e.g., sodium caprylate, sodium N-acetyltryptophanate
• Disease in which the use of albumin may be deleterious, e.g., decompensated heart failure or traumatic brain injury
• Disease situations in which albumin administration may be advantageous, e.g., hepatorenal syndrome, nephrosis, burns, and intestinal malabsorption syndrome
• Lactation

5.1.4 Measures to prevent participation in further studies

The trial centres shall ensure that patients are not included in any other interventional clinical trial during the course of the clinical trial, up to (and including) the 90th day after randomization. The trial centres will ensure that ARISS participants do not participate in ARISS again during the clinical trial recruitment period.

5.2 Informed consent

Informed consent will be obtained verbally and in writing using specific forms. The declaration of consent to participate in the clinical trial must be personally dated and signed by the consenting person.
The declaration of consent must be signed in duplicate. One copy (original) will remain in the trial centre and must be kept in the investigator site file (ISF). The second copy (copy or 2nd original) will be handed to the consenting party together with the information document (explanatory document) and the general conditions of insurance. Participation of a patient in the clinical trial and delivery of the documentation and further information on the clinical trial should be noted in his/her medical record.

5.2.1 Patients able to consent

Eligible patients must be informed of the study by the investigator or a physician (member of the trial group) prior to initiating the study-related intervention. Informed consent must be obtained verbally and in writing including the nature of the study, objectives, duration, course, benefits, all risks and adverse effects, as well as other aspects of the clinical trial or use of the trial product. The informing physician must be sure that the information about the trial is understood by the patient. After informing the patient about the trial, each patient should be given enough time and opportunity to ask questions and decide on their participation.
The patient must sign and date his/her consent to participation in the clinical trial on the consent form. The patient's consent must also expressly refer to the collection and processing of information about his/her health condition. Therefore, the patient should be explicitly informed about the purpose and scope of the trial and the use of personal data, in particular health-related data.

If a patient is unable to sign the informed consent personally, but can give verbal consent, at least one independent witness must be present during this process. The witness may not be a person employed by the trial centre or a member of the trial group. The verbally given consent must be documented in writing by the witness who must sign with date and time the consent form to confirm the patient's oral consent.

5.2.2 Patients who are unable to consent

Because of the expected high severity of illness in the trial population, most of the eligible patients will be unable to consent. In this case, it will not be possible to obtain written consent from the patient prior to initiating the study-related measures.
In patients with septic shock, early therapy is a crucial determinant of prognosis of these patients. A therapeutic benefit from the intervention -albumin administration in the albumin group- can likely only be demonstrated if the clinical trial is started within the first few hours of the diagnosis of septic shock.
Since, according to international guidelines, early initiation of therapy is one of the essential standard therapies for severe sepsis and septic shock, it may be justified to begin the clinical trial in the existing emergency situation (septic shock) without informed consent of a legal representative or a guardian. According to AMG, participation is legally possible even without informed consent if this cannot be obtained due to an emergency situation. These patients can be included in the study according to § 41 Abs. 1 AMG (29) which states that: if informed consent cannot be obtained because of an emergency situation, treatment indicated to be given without delay to rescue the life of the concerned persons, to restore their health or to minimize their suffering, can be promptly initiated. Consent for further participation must be obtained as soon as possible and reasonable.

In this case, the trial centers should adopt the locally established procedure for including patients who are unable to consent and follow the recommendations of the local ethics committee. The presumed will of the patient should be considered. If she/he would refuse to participate, the patient should not be included in the clinical trial.

Patients unable to consent – with a guardian or legal representative
consent to participate in the clinical trial may be given in writing by the guardian or legal representative. A copy of the act of attorney or the identity card must be kept in the trial centre (ISF or separately).
If the guardian or the legal representative considers that the patient would not have wanted to participate in the trial, or if the guardian or the legal representative refuses participation, the patient will not be included in the clinical trial.

In the case of patients who are unable to consent and who have appointed a guardian or have a legal representative (supervisor) with a corresponding task designation, consent to participate in the clinical trial should be in accordance with § 41 (3) no. 2 AMG (29). The informed consent to participate in the clinical trial by the designated guardian or supervisor must be given in writing as soon as possible. A copy of the act of attorney or supervisor identification card must be kept at the trial center. If the guardian or supervisor refuses participation in the study, the patient will not be included in the clinical trial.
If the guardian or the supervisor cannot be reached in a timely manner (max 72 h) despite repeated attempts or if she/he cannot attend in person to the trial center, the trial center must decide how to proceed in this specific patient (for example, to consider the independent consultant procedure).
.
Patients unable to consent – without a guardian or legal representative
For patients who are unable to consent and have no guardian or legal representative, inclusion in the trial should be in accordance with the recommendations of the local ethics committee. The trial centres should adopt the locally established procedure for including these patients.
If a legal representative is required, this must be done at the latest within 72 hours. The investigator should arrange for identification of a suitable person and apply to the respective court.
It is crucial, if necessary by telephone, to inquire from the patient's relatives about whether the patient would likely have wanted to participate in clinical trials and to confirm this in

writing. If the conversation with relatives reveals that it is likely the patient would not have wanted to participate, the patient will not be included in the clinical trial. If the patient is known to want to participate in clinical trials or if the patient's relative agrees to participation in the study (provisionally), this information will be forwarded immediately to the competent court, which will promptly appoint a legal representative. In this case, the patient will be included in the clinical trial. Subsequently, with this provisionally granted consent, the written consent of the appointed legal representative must be obtained as soon as possible. If the patient dies before the legal representative has been appointed or consented, the patient should not be excluded from the analysis. If SAEs have occurred, they must be pursued further in accordance with the relevant requirements.

In this case, the trial centers should adopt the locally established procedure for including patients unable to consent and follow the recommendations of the local ethics committee. The appropriate procedure must be documented.

If, for example, the appointment of a legal representative (supervisor, health care task designation) is required, this must be done as soon as possible (at the latest, however, within 72 hours).
Written consent to the clinical trial or its continuation by the legal representative must be obtained. If this does not occur within a maximum of 72 hours of setting up the legal representation, or if the legal representative refuses (further) participation in the study, the patient will not be included in the clinical trial or his / her participation in the clinical trial will be terminated.
If SAEs have occurred before the decision of the legal representative, theses will be prosecuted in accordance with the applicable requirements.
If the patient dies before the legal representative has been appointed or has decided to participate, this must be documented accordingly in the eCRF.

The "independent medical consultant procedure"
In this procedure, confirmation of the urgency of participation in the clinical trial and possible benefit to the patient may be obtained by an independent consultant and the patient subsequently becomes a participant in the clinical trial.
The consultant must be a specialist and have at least 6 months experience in intensive care medicine. The consultant should not be involved in the clinical trial or belong to the study department and should not be part of the patient's care team. The consultant must justify his/her decision in writing. Thereafter, inclusion of the patient is possible. The investigator must initiate appointment of a legal representative no later than 72 hours after inclusion in the clinical trial. Written consent for continuation of the clinical trial must be obtained from this legal representative. If this does not occur within a maximum of 72 hours of the establishment of a legal representative or she/he refuses participation in the study, participation in the clinical trial will be terminated for this patient. If SAEs have occurred, they must be pursued further in accordance with the relevant requirements.
If the patient dies before a legal representative has been appointed or decided to participate in the study or not, this should be documented in the eCRF accordingly.
.

5.2.3 Patients who regain their ability to consent

All patients who regain their ability to consent for participation in the trial should provide their informed consent to continue in the trial, as long as their health condition during hospitalization permits. If a formerly incapacitated patient decides at this time or later not to participate in the clinical trial, he/she may withdraw his/her from participation at any time and without stating a reason, without incurring any disadvantages for his/her further medical treatment. Patients in the albumin group may decide at this time or later not to continue administration of the trial product (discontinuation of treatment). The data collected until withdrawal from participation in the trial will be considered in the statistical analysis.

5.2.4 Information in the case of changes in course of the trial

Participants, guardians or legal representatives will be promptly notified if significant new findings or information about the trial product emerge during the clinical trial that may influence their willingness to continue to participate in the clinical trial. Disclosure of this information will be documented. The patient, guardian or legal representative will receive a copy of any changes or amendments to the patient information and a copy of the consent form, which may need to be re-signed and dated.

5.3 End of participation in the clinical trial

On study day 90 after randomisation, data collection and thus participation in the trial will end for the respective patient.

5.4 Prerequisites for participation in the trial

The ability to determine serum albumin concentrations and provide the results on the same day is a prerequisite for participation of a trial centre in the clinical trial. Albumin concentration can be measured using analytic methods available in the respective trial centre, its local or commissioned laboratory. The trial centre is responsible for quality control measures relating to the laboratory method used as part of Good Laboratory Practice.

The qualifications of the trial centre, investigators, deputy and members of the trial group will be recorded before the start of the clinical trial. The investigator and his/her deputy must be qualified in accordance with the requirements for clinical trials according to the AMG and local ethics committees. The investigator must be an intensive care physician, expert in the treatment of septic shock who treats patients with this condition regularly. He/she must appoint at least one deputy with comparable qualifications.

The investigator must know and follow the legal and judiciary obligations as well as the principles of the ICH-GCP (30). According to § 40 AMG (1a) (29), the investigator should determine suitably qualified members of the trial group. He/she will guide and supervise them and provide them with the information necessary for their work in the conduct of the clinical trial, in particular the protocol and investigator information.

The pharmacy of the respective trial centre or ICU must be able to store the trial product correctly in accordance with the trial centre qualification and the storage precautions required by the relevant technical information.

All eligible centres will be screened for their suitability as a trial centre by means of a selection visit. The recruiting potential of the trial centres has already been requested in the context of the application for the DFG grant.

Patient data will be recorded electronically (electronic CRF, eCRF). To do this, the test centres need a computer with an Internet connection.

.

6. Trial drug

The trial drug is the approved commercial product "Albutein® 200 g/l or Plasbumin® 20, solution for infusion". Both drugs are manifactured according to the same procedure and considered to be interchangeable. These product will be provided free of charge by Instituto Grifols SA (Barcelona, Spain), exclusively for the treatment of patients in the albumin group. The control group will be treated according to usual practice for patients with septic shock.

6.1 Labelling of the trial drug

Special labelling of the trial drug will be waived in accordance with § 5 (9) of the GCP Regulation (31). The trial drug is an approved product intended for use in the clinical trial without additional manufacturing measures. The design of the clinical trial makes it possible to dispense the trial drug without special labelling on the containers and the outer wrapping according to § 5 paragraphs 2 to 7.

According to the Transfusion Act, the product name and the charge number must be documented for each treatment of a patient with Albutein® 200 g/l or Plasbumin® 20 to ensure the patient can be traced the patient and the charge used. This will be done, for example, by labelling the albumin vials (using the study acronym, EudraCT number, and Patient ID), via the consignment note in the patient record, and via the drug accountability log in the ISF.

6.2 Drug Accountability

6.2.1 Transport by the manufacturer

The trial drug will be supplied by the manufacturer to the pharmacy of the Jena University Hospital.

6.2.2 Storage and delivery to the trial centres or their pharmacies

The pharmacy of Jena University Hospital will be responsible for the storage as well as the transport of the trial drug to the trial centres or their local pharmacies.

The trial drug should be stored below 25°C and may not be frozen.

The first delivery of the trial drug to a trial centre or its pharmacy by the pharmacy of Jena University Hospital Jena will be commissioned by the SpB. Each subsequent delivery of the trial drug will take place without approval of the SpB. Subsequent deliveries will be requested independently by Jena University Hospital pharmacy. All deliveries will be documented in the trial master file (TMF) and the ISF. Confirmation of the receipt of each delivery should be done in writing.

6.2.3 Storage and provision by the pharmacy of the trial centres

The pharmacy of the respective trial centre confirms the receipt of the delivered trial drug in writing. It is responsible for the correct storage of the trial drug in a room, secured against non-authorized access. It is also responsible for and short-term provision of the trial drug. The use of the trial drug outside the clinical trial should be prohibited.

6.2.4 Storage, use and tracing by the trial centre

Each trial centre should confirm the receipt of delivered trial drug in writing.

The respective ICU of the trial centre should be able to correctly store the trial drug. The respective investigator is responsible for the storage of the trial drug in the trial centre, which should be secured against unauthorised access.
Administration of the trial drug per participant may only be carried out by the persons specified in the list of responsibilities and must be documented in writing in the ISF. The trial drug may only be used in the context of this clinical trial. It is usable until the given expiry date.
Empty and opened bottles of the trial drug per participant will be collected and destroyed in the trial centre according to the specifications in the study manual. These processes will be documented accordingly.

6.3 Administration of the trial drug (only in the albumin group)

6.3.1 Start dose

Administration of the initial dose of the trial drug must be started within 6 to 24 hours after the beginning of the septic shock. A maximum of 2 hours may elapse between randomisation and the start of administration of the initial dose.
Before the initial dose is given, a blood sample must be drawn to determine the serum albumin concentration.

Starting dose: 60 g human albumin 20% (200 g/l, infusion solution) over 2-3 h
Application: The investigational product will be administered intravenously using an infusion device. The human albumin should not be mixed with other medications, whole blood or packed red blood cells.

The study drug is ready to use and can be administered intravenously directly or after dilution with an isotonic solution (e.g., 5% glucose or 0.9% sodium chloride). Albumin solutions should not be diluted with water for injections as this may cause haemolysis in the treated patients.
The solution should be clear or slightly opalescent. Infusion solutions that are cloudy or have sediment should not be used. This may suggest that the product is unstable or the solution is contaminated. After opening the packaging of each vial, the solution should be used immediately.

6.3.2 Subsequent administration of the trial drug

Subsequent doses of the trial drug, starting from study day 1 will depend on the routine treatment day at each trial centre. A day begins when a new daily file is created at the trial centre. Study day 1 is thus defined as the 1st full day of treatment (24 h) after the day of randomisation.

Administration of the trial drug will be based on the serum albumin concentration measured each day. Dose adjustment will follow a predetermined schedule with the aim of maintaining a serum albumin concentration of at least 30 g/l.

Dose adjustment according to serum albumin concentration: ≥ 30 g/l: no administration
≥ 25 g/l and <30 g/l: 40 g over 1-2 h
≥ 20 g/l and <25 g/l: 60 g over 2-3 h
<20 g/l: 80 g over 3-4 h

6.3.3 Scheduled discontinuation of the trial drug

Administration of the trial drug will continue for a maximum of 28 study days after randomisation and only as long as the participant is being treated in the ICU.

6.4 Premature discontinuation of the trial drug

Administration of the trial drug may end prematurely in the following conditions:
• Discharge of the participant from the ICU before day 28 after randomisation
• Death of the participant
• Failure to obtain consent to continuation of the clinical trial from the guardian or the legal representative according within the respective time window in patients who participated after confirmation of the urgency of participation in the clinical trial by an independent consultant or other procedure established in the trial centre to include patients who are unable to provide informed consent
• Withdrawal of consent to participate in the clinical trial
• Termination of treatment at own request, withdrawal of consent for participation
• Occurrence of a medical reason, as described under 6.5

Termination of treatment at own request
Each participant (or, in the case of a non-consenting patient, his/her guardian or legal representative) has the right to opt-out at any time (no further administration of the trial drug) and without stating reasons, without incurring any disadvantage for the further medical treatment of the patient.
The patient (or guardian or legal representative) will be asked to give the reasons for this decision, but without any obligation to do so.
Observation and documentation for this patient will be continued.
The SpB must be informed about the cancellation of the treatment at own request.

6.5 Discontinuation of the trial interventions- discontinuation due to a medical reason

The decision to discontinue or to continue treatment with the trial drug from a medical point of view will be at the discretion of the treating physicians (members of the trial group).
Premature discontinuation of treatment with the trial drug does not lead to termination of the study. Further observation and documentation will be continued.

Human albumin is a normal component of human plasma and does not differ in its effects from physiological albumin. According to the current drug information brochure, toxicity tests for single-use human albumin in animals are of little relevance and do not enable determination of the toxic or lethal dose or a dose-response relation. The target serum albumin concentration in the clinical trial is within the normal range of human plasma albumin (mindestens 30 g/l), so that overdose is unlikely.

According to the current drug information brochure, the following adverse effects may occur with Albutein® 200 g/l or Plasbumin® 20:
• Mild reactions, such as flush, urticaria, fever, and nausea, are rare.
• In very rare cases, anaphylactic shock can occur.
• The possibility of transmitting an infection cannot be completely ruled out despite standard measures of preventing infections caused by drugs made from human blood or plasma. This also applies to all unknown or newly emerging viruses or other pathogens.

MIld reactions, such as flush, urticaria, fever and nausea, usually disappear quickly when the infusion rate is reduced or the infusion is stopped.
If such reactions occur, appropriate therapy should be initiated if clinically indicated. This includes therapy with antihistamines and symptomatic therapy.
In these cases, the trial drug should be subsequently administered over a longer period of time (up to twice the prescribed duration), with close monitoring of the relevant clinical

parameters. A new clinical evaluation and, if necessary, treatment with the trial drug should continue on the following day. If the reaction reoccurs, the trial preparation will be permanently stopped.

If anaphylactic shock occurs, infusion of the trial drug should be discontinued and adequate treatment should be initiated according to current recommendations for treating shock. Administration of the trial drug will be permanently stopped.

If hypervolaemia is suspected, the trial drug should be administered over a longer period of time (up to 2x the prescribed duration) under close observation of the appropriate haemodynamic parameters. If the association between the trial drug and hypervolaemia is confirmed, an increase in the severity of hypervolaemia is observed, or life-threatening consequences such as pulmonary oedema occur, administration of the trial drug for that day should be stopped. A new clinical evaluation and, if necessary, continuation of the trial drug should take place on the following day.
The SpB must be informed about the permanent termination of the trial drug (discontinuation of treatment). The patient, guardian or legal representative will be informed about discontinuation of treatment for medical reasons as well as the follow-up procedure.

6.5.1 Compliance

Compliance of administration of the trial drug by the patient need not be monitored, because the drug will always be administered by the attending physician (member of the trial group) or caregiver on the ICU or checked by these persons and documented in the medical record.

6.6 Instructions for the control group without albumin

Patients in the control group are treated according to standard practice with any crystalloid solution as volume replacement therapy of first choice. In patients of the control group, albumin administration should not be considered. If, however, according to the judgment of the treating physician (member of the trial group), it is necessary to consider albumin replacement therapy for clinical reasons, this can of course be done. This may be the case if, for example, the serum albumin level decreases below 15 g/l. The following factors should be considered:

• Active substance of the administered product: Albumin, but do not use the product provided for use in the trial!
• Application: intravenous, following the instructions in the respective drug information brochure
• Dosage: as low as possible
• Documentation of the drug

The administration of albumin to a participant in the control group leads to exclusion of this patient from the per-protocol analysis.

6.7 Permitted concomitant medication, concomitant therapies

There are no restrictions to concomitant medication and therapies during the trial. Concomitant medication and therapies must be documented.

6.8 Further treatment of participants after the end of participation

Specific aftercare or follow-up is not needed after a maximum of 28 study days of administration of the trial drug to patients in the albumin group, or after discharge from the ICU or withdrawal of consent in both groups. Treatment with the trial drug is an intensive care measure that is of no consequence to subsequent medical care. The administration of

human albumin 200 g/l solution for infusion as a trial drug while participating in a clinical trial will be communicated to the treating physician in the discharge report.

7. Start, course and end of the clinical trial/study visits

General remarks:

- Patients in the participating ICUs will be screened during the ICU stay to see whether they fullfil the inclusion criteria and do not meet exclusion criteria. Informed consent for participation in the trial will be sought from all patients ≥18 years of age with septic shock.
- General treatment of patients participating in the clinical trial will follow usual practice for this condition and will be consistent with current guidelines for the treatment of such patients (25).
- Crystalloids will be administered in both groups according to usual practice.
- Antimicrobial therapy should be performed according to PK/PD principles. Increasing the dosage of antimicrobial agents with high protein binding capacity should be weighed in patients with hypoalbuminemia.
- Collection of microbiological samples as part of the primary diagnosis of sepsis or of developing infections is at the discretion of the treating physician and is not trial-related. Nonetheless, microbiologic diagnostics should be sought, especially in the form of blood cultures, before starting antimicrobial therapy.
- Trial-related measures may only take place after consent!
- A patient may be included in the trial only if all the inclusion criteria (including negative pregnancy test) are met and all exclusion criteria are absent.
- In the case of a woman of childbearing potential, a pregnancy test should be carried out after obtaining informed consent. Before the result is available, the patient must not be randomised. If the pregnancy test is positive, participation in the clinical trial for the patient will end promptly.
- A study day is defined by the treatment day according to the routine practice of the respective trial centre. A study day changes when a new daily data record sheet is created in the trial centre. The 1st day after randomisation (1st study day) is the 1st full treatment day (24 h) after the day of randomisation.

7.1 Screening and 24-h period before randomisation

- Obtaining written informed consent or confirming the urgency of participating in the clinical trial with possible patient benefit by an independent consultant or other procedures established in the trial centre to include patients who are unable to provide informed consent
- Checking the inclusion and exclusion criteria (including pregnancy test (blood or urine) for women of childbearing age)
- Recording the time of onset of septic shock
- Collection of basic data (sex, age, weight, height, time of hospital admission, time of ICU admission, type of admission, referring facility prior to transfer to the ICU, etc.)
- Documentation of primary or secondary admission diagnoses
- Documentation of concomitant diseases present on admission to the ICU
- Collection of data on infection, microbiology and anti-infective therapy
- Collection of raw data for the calculation of APACHE II, SAPS II, and SOFA scores from the routine laboratory requests (not for study purposes) from the 24-h period before randomisation (data from ICU or normal ward) (see Appendix)
- Assessment of concomitant medication (catecholamines, inotropes, diuretics, fluid therapy including transfusion, adjunctive sepsis therapy) from the 24-h period prior to randomization
- Recording of intensive care interventions (intubation (ITN), central venous catheter (CVC), arterial catheter (AC), renal replacement therapy (RRT), ventilation, extracorporeal

membrane oxygenation (ECMO), haemodynamic monitoring during the 24-h period before randomisation
- Recording of clinical parameters (body temperature, respiratory frequency, haemodynamic parameters (SAP, MAP, DAP, CVP, CO) from the 24-h period before randomisation

7.2 Time of randomisation

Randomisation leads to the assignment of a participant to the albumin group or to the control group without albumin.

The **randomisation day** is defined as the treatment day on which the randomisation takes place. It ends when a new daily treatment sheet is created.

Randomisation must be performed within two hours of the written informed consent or confirmation of urgency of participation in the clinical trial with possible benefit to the patient by an independent consultant or other procedures established in the trial centre to include patients who are unable to provide informed consent
The randomisation is carried out by an automated internet-based service, which is provided by the Centre for Clinical Studies Jena (ZKS). The group assignment is retrieved via a validated electronic tool (PaRANDies) by the respective trial centre. The group assignment of the patient must be documented (patient record, e-CRF). Each participant will receive a randomisation number.

- Collection of data on infection, microbiology and anti-infective therapy
- Recording of clinical parameters (body temperature, respiratory frequency, haemodynamic parameters) at the time of randomisation (+/- 1 hour)
- Recording of concomitant medication (catecholamines, inotropes, diuretics, fluid therapy including transfusion, adjunctive sepsis therapy) at the time of randomisation (+/- 1 hour)
- Collection of routine data from the laboratory (not trial-related), performed in the 24-h period before randomisation: haemoglobin, creatinine, bilirubin, C-reactive protein, procalcitonin, leucocytes, platelets, lactate, arterial blood gas analysis (PaCO2, PaO2, FiO2, SO2, pH , ScO2)
- Recording of intensive care interventions (ITN, CVC, AC, RRT, ventilation, ECMO, haemodynamic monitoring) at the time of randomisation (+/- 1 hour)
- Start capturing AEs, SAEs, sepsis-related clinical events

7.3 Time period until 2 h after randomisation

- Collection of data on infection, microbiology and anti-infective therapy
- Capture of AEs, SAEs, sepsis-related clinical events
- Procedure according to group allocation:

Albumin group:
1. Blood sampling to determine serum albumin concentration within 2 hours after randomisation
2. Starting dose of the trial product within 6-24 h after the onset of septic shock: 60 g Human albumin 20% (200 g/l solution for infusions) over 2-3 h
3. Data collection on trial drug administration (continuous collection).

Control group without albumin:
1. Blood sampling to determine serum albumin concentration within 2 hours after randomisation
2. Treatment according to usual practice with crystalloids as the first-choice fluid treatment.

7.4 Trial visit at 6 h after randomisation

- Collection of data on infection, microbiology and anti-infective therapy
- Recording of co-medication with catecholamines and inotropes at this specific time-point (+/- 1 hour)
- Recording of concomitant medication with diuretics, fluid therapy including transfusion, adjunctive sepsis therapy) during the past 6 h
- Recording of clinical parameters (respiratory frequency, haemodynamic parameters) at this specific time point (+/- 1 hour)
- Collection of routine laboratory data (not trial-related) at this specific time point (+/- 1 hour): haemoglobin, lactate, arterial blood gas analysis (PaCO2, PaO2, FiO2, SO2, pH, ScO2)
- Recording of intensive care interventions (ITN, RRT, ECMO, mechanical ventilation, haemodynamic monitoring) from the previous 6 hours
- Capturing of AEs/SAEs and sepsis-related clinical events (continuous)
- Recording vital status (alive/dead)
- Data collection about administration of the trial drug (continuous), including information on the administered dose, interruption or discontinuation of the dose if necessary

7.5 Trial visits on trial days 1 to 28 after randomisation

These visits should only be performed if the patient is still being treated in the ICU.

• Blood sampling to determine the serum albumin concentration, including recording the time of blood sampling
• Blood samples for serum albumin concentration determination may be performed as part of the routine blood sampling procedure at the respective trial centre, provided that they are taken between 4:00 and 9:00 in the morning.
• Collection of data on infection, microbiology and anti-infective therapy
• Recording of the raw data for calculation of the SOFA score on the respective study day from the routine laboratory results (not for trial purposes); the "worst" daily value should be recorded
• Recording of concomitant medication with catecholamines and inotropes, diuretics, fluid therapy (including transfusion therapy, adjunctive sepsis therapy) on the respective trial day
• Recording of intensive care interventions (ITN, CIC, AC, RRT, mechanical ventilation, ECMO, haemodynamic monitoring) on the respective trial day
- Recording the amount of enteral and parentral fluid adminstration on the trial day

• Collection of routine laboratory data (not trial-related), taken between 4:00 - 9:00 in the morning: haemoglobin, creatinine, bilirubin, C-reactive protein, procalcitonin, leucocytes, platelets, lactate, arterial blood gas analysis (PaCO2, PaO2, FiO2, SO2, pH, ScO2)
• Recording of clinical parameters (body temperature, respiratory frequency and haemodynamic parameters), 24 h urinary output, other fluid loss on the trial day
• Capturing of AEs/SAEs and sepsis-related clinical events (continuous)
• Recording of vital status (live / dead)
• Albumin group: administration of the trial drug according to serum albumin concentration, dose adjustment according to the given scheme
• Data collection regarding administration of the trial drug (continuous), including information on the administered dose, interruption or discontinuation of the dose if necessary
Note: After administration of the trial drug on day 28, no further doses are given.

7.5.1 Data collection at hospital discharge or at the end of the study

Data will be collected from participants at one of the following time points, whichever comes first:

• Discharge from the hospital before or on trial day 28
• Discharge from the hospital up to and including trial day 90
• Trial day 90 reached in the hospital (regular end of study)
• Early termination of the clinical trial as described in 7.6

Data collection:

• Recorde data on ongoing SAEs and serious sepsis-related clinical events
• Vital status (alive/dead): if applicable date of death and cause of death; if applicable, recording of "end of life" decisions, stay after discharge
• ICU length of stay (of the first ICU stay after study participation)
• Hospital length of stay (first hospital stay after study participation)

If the patient is discharged from the ICU or the hospital during the 90-day observation period, this time will be defined as the endpoint for determining the ICU length of stay and/or hospital stay. If the patient returns to the ICU or hospital within the 90 days, this is no longer relevant for the collection of trial endpoints.
.

7.5.2 Data collection at trial days 28, 60, and 90 after randomisation

At these time points, data will be collected on the primary and secondary "mortality" outcomes. If the patient has already left the hospital before these time points, contact with the patient, his guardian or legal representative will take place to collect the required data.

• Vital status (alive/dead): if applicable date of death and cause of death; if applicable, recording of "end of life" decisions, stay after discharge
• Verification of the documentation of SAEs already detected and serious sepsis-related clinical events, possibly AEs, which are "ongoing" at the respective time point

7.6 Premature end of the participation of a patient in the clinical trial

Participation of a patient in the clinical trial may be prematurely terminated for the following circumstances:

• Death of the participant

• Failure to obtain consent to the continuation of the clinical trial by a guardian or a legal representative according to the respective time windows in patients who participated after confirmation of the urgency of participation in the clinical trial by an independent consultant or other procedures, established in the trial centre to include patients who are unable to provide informed consent

• Refusal to continue the clinical trial by formerly incapacitated patients who have regained their ability to consent

• Abort at own request, withdrawal of consent to participate in the clinical trial

• Occurrence of any of the circumstances described in 7.7.2 and 7.7.3

Termination of participation in the trial, drop-out at own request

Participation in the clinical trial is voluntary. Each participant has the right, at any time and without stating reasons, to withdraw his/her consent prematurely from the clinical trial without incurring disadvantages for his/her further medical treatment. Withdrawal of consent leads to termination of the clinical trial in the patient.

• The patient, his/her guardian or legal representative will be asked to give the reason for his decision, but without any obligation to do so.

• The patient, his/her guardian or legal representative will be informed that no further study-related measures will be carried out, but that the stored data may continue to be used, as far as is necessary to determine the effects of the intervention and to ensure that patient's interests will not be affected.

• The patient, his/her guardian or legal representative should be asked to provide the data requested at the end of the study.

• The patient's observations and documentation related to the clinical trial will be terminated.

• The SpB must be informed of the termination of participation at own request.

7.7 End of the clinical trial

7.7.1 Scheduled end

The scheduled end of the clinical trial is defined as the date on which all follow-up data have been recorded and monitored in the eCRF. According to GCP-V § 13 (30), the sponsor will inform the competent authority, the competent higher federal authority and the responsible ethics committee within 90 days of the termination of the clinical trial. Within one year of completion of the clinical trial, the sponsor will submit a summary report of the clinical trial covering all results of the clinical trial to the competent higher federal authority and the relevant ethics committee.

7.7.2 Premature termination of the trial in a trial centre

The SpB will decide on the interruption or termination of the clinical trial in a trial centre, possibly in coordination with the Safety Committee and the biometrician.

The clinical trial may be discontinued or terminated at a trial centre if

• the trial centre does not meet the technical requirements specified in the study protocol,

• the procedures related to the clinical trial do not comply with the protocol,

• there are serious, unexplained problems with the quality of the collected data (insufficient quality of data),

• the recruitment rate in the trial centre is inadequate,

• unpredictable circumstances have occurred in the respective trial centre that do not allow continuation of the clinical trial (e.g., lack of personnel and equipment capacity, the required logistics can no longer be applied)

If there are ethical concerns about the continuation of the clinical trial by an investigator, deputy, or a medical member of the trial group, this must be reported immediately to the SpB. If termination of the clinical trial in a trial centre is initiated by the trial centre itself, the latter must give written reasons for this decision.

Investigators, deputies, or members of the trial group at a trial centre who are no longer participating in the clinical trial must inform SpB immediately about their decision. The decision must be justified.

It is the responsibility of the investigators and the SpB to comply with the disclosure, documentation and notification requirements of GCP-V (30).

In the event that a trial centre has to be closed prematurely, a final visit will be made by the trial monitor. Details are described in the Monitoring Manual.

7.7.3 Premature termination of the entire clinical trial

The SpB is entitled to interrupt or prematurely terminate the clinical trial for relevant medical and administrative causes. Any interruption or premature termination of the entire clinical trial should be discussed in advance with the SMC. The reasons for interrupting or terminating the trial should be documented in detail.

The SpB is entitled to suspend or terminate the entire clinical trial if:

• the recruitment rate is inadequate,

• there are serious, unexplained problems with the quality of the collected data,

• unacceptable risks have arisen (decision after new benefit-risk assessment),

• new scientific evidence does not allow continuation of the clinical trial during the clinical trial period, or

• a risk to patient safety emerges.

Since the clinical trial is carried out in accordance with the provisions of the German Medicinal Products Act, the responsible BOB can also revoke the license or cancel it.

The trial procedures for all patients whose participation in the clinical trial has not yet ended must be determined.

The participant must be informed immediately of any interruption or early termination of the trial. Appropriate further treatment or follow-up must be guaranteed. The date and reason for the interruption or premature termination must be documented.

The data available until early termination must be evaluated and a final clinical trial report must be prepared. If clinical examination is discontinued or interrupted by the SpB, the trial centres should be informed. In accordance with GCP-V § 13 (8) (30), the SpB should inform the competent state authority, the competent BOB and the responsible ethics committee within 15 days, stating the reasons for the termination or interruption of the trial. It is the responsibility of the investigators and the SpB to comply with the duties of disclosure, documentation and notification under GCP-V.

Details of the final visit of the trial monitor in the trial centres are described in the Monitoring Manual.

8. Safety and adverse events

8.1 Assessment of the safety of the trial drug

The trial drug contains human albumin as an active substance. Human albumin is a normal component of human blood plasma and does not differ in its effects from physiological albumin. According to the valid information brochure, there are only a few rare or very rare adverse effects. Data on safety and compatibility are described in the valid information brochure. For the clinical trial, safe application can be assumed. Safety management will ensure patient safety for the clinical trial. In addition, adverse events observed during the clinical trial will be recorded, evaluated, and regularly reviewed by an independent safety committee.

8.2 Adverse events

According to GCP-V § 3 (30), an Adverse Event (AE) is any unfavourable event that occurs to a patient participating in a clinical trial who has received an investigational medicinal product and that is not necessarily causally related to the treatment. According to ICH-GCP (29), these may be symptoms (including, for example, abnormal laboratory values), diseases or signs that are associated with the use of an investigational medicinal product. This is independent of whether the event is causally related to the investigational product or not. For the ARISS study, assessment of adverse events will also be done in patients of the control group without albumin.

Septic shock, which is the basic inclusion criterion of this clinical trial in all study patients, is one of the most life-threatening clinical pictures. The ICU mortality from severe sepsis and septic shock is 47% according to the prevalence study of the sepsis competence network (7). Many of the patients need mechanical ventilation and about half the patients have acute kidney failure. Hepatic and metabolic parameters are almost always altered depending on the severity of sepsis. Previous studies investigating severe sepsis or septic shock, such as MAXSEP (EudraCT 2006-006984-21) and SISPCT (EudraCT 2007-004333-42), have therefore performed selective AE evaluation that has been shown to be reliable and which should also be used in the ARISS study.
Because most septic patients have altered laboratory values and symptoms because of the underlying disease and are constantly evolving as the disease progresses, the definition of AE is restricted as follows in this clinical trial:
An ***adverse event in the ARISS*** is any adverse event that occurs to a study subject that is not necessarily causally related to the trial drug. If an adverse event can be explained by the underlying sepsis, the following applies:

Events plausibly explainable by sepsis are recorded in both groups as sepsis-related clinical events in the daily eCRF visits, but not as AEs. This rule concerns the following clinical events:

- Death caused by severe sepsis or septic shock
- Cardiovascular event requiring the administration of vasoactive substances
- Respiratory event: e.g., decrease in PaO2/FiO2 ratio, hypoxia, ARDS, acute pulmonary dysfunction, and mechanical ventilation
- Hepatic event: e.g., Liver failure or liver dysfunction
- Renal event: e.g., Kidney failure, renal insufficiency
- Haematological event: e.g., coagulopathy, DIC, thrombocytopaenia, thrombocytosis
- Neurological event: e.g., delirium, confusion

Each sepsis-related clinical event must be documented in the eCRF during the respective visit with specific parameters. The investigator assesses the sepsis-related clinical event in

terms of an association with the trial drug and classifies it as "possible" or "not possible". Documentation of the sepsis-related clinical event as an AE occurs only if the examiner suspects a connection with the administration of the trial drug, which is therefore only possible in the albumin group.
However, the following effects of the trial drug may be substance-specific and must be documented as AE:

• Flush
• Urticaria
• Fever
• Nausea
• Anaphylactic shock
• Hypervolaemia
• Pulmonary oedema
• Transmission of infection (in addition to sepsis, see corresponding section of the valid information brochure).

Clinically relevant worsening of a pre-existing disease not related to sepsis should be considered and documented as an AE.
.

8.3 Duration of recording adverse events

Recording of AEs and sepsis-related adverse events begins at randomisation. Because the study drug is human albumin, which is a normal constituent of human blood plasma, and does not differ in its effects from physiologic albumin, recording and follow-up of AEs and SAEs for a maximum of 24 hours after the last dose of the trial drug is considered to be sufficient. Therefore, AEs and sepsis-related adverse events will be recorded
• in the albumin group until 24 h after the last dose of the trial drug and
• in the control group without albumin until day 28 after randomisation or until discharge from the ICU, if it occurs before day 28 after randomisation.

In the event that AEs or SAEs are still "ongoing" after the above dates, they will be tracked until maximum the end of data collection (Day 90). If they are "ongoing" on day 90, they are documented as "not recovered," "recovered with sequelae," or "unknown."
.

8.4 Documentation of adverse events

All AEs will be recorded promptly by the trial centres in the eCRF. Sepsis-related clinical events will be documented in the daily visits section, all others in the AE section of the eCRF.

<u>Events which should not be documented as an AE:</u>
• Medical or surgical interventions (diseases or symptoms that lead to a necessary intervention are documented as an AE)
• Interventions to treat a pre-existing condition that were already planned before consenting to the clinical trial,
• Daily fluctuations in the clinical picture,
• Normal progression of the disease severity of sepsis,
• Illnesses that existed before consenting to the clinical trial (this will be documented as concomitant disease and not AEs).

Each AE is to be checked to see whether it meets the criteria for an SAE and, if necessary, proceed according to the SAE declaration procedure (see corresponding chapter 8.6).

At the trial centre, care should be taken to ensure that all persons involved in the treatment of participants are adequately informed about the responsibilities in the event of adverse events.

8.4.1 Intensity of an adverse event

The intensity of adverse events will be stratified according to the following 3-point scale.

• Mild: a clinical symptom or sign that is well/easily tolerated and usually requires no intervention
• Moderate: clinical symptom or sign sufficient to interfere with normal/daily activity, intervention may be required
• Severe: a clinical symptom or sign that results in severe disability, inability to work or inability to perform everyday activities = daily activities/work not possible, treatment or intervention usually required.

If the intensity of an event changes, only the higher intensity should be documented on the corresponding AE section. If an AE decreases in intensity, the previous rating must not be changed.

Mild, moderate, and severe adverse event can be serious or not. The above terms describe the intensity (medical severity) of certain events (e.g., a mild, moderate or severe myocardial infarction). However, the event may also be of secondary medical relevance (e.g., severe headache) and it is not necessarily "serious." "Seriousness" serves in the sense of the law as a definition for further reporting obligations.

8.4.2 Causality – relationship between the adverse event and the trial drug

For each AE, whether an association with the trial drug can be excluded or not will be assessed.

The type and pattern of response, timing of administration, clinical status of the patient, concomitant medication and other relevant clinical parameters should be considered.
The following classification is used for the assessment of causality:

yes - possible
• There is a justified causal relationship between the trial drug and the event
• The event reacts to the discontinuation of the trial drug
• The event appears on resumption of the trial drug (if clinically feasible)
• Information on discontinuation of the trial drug is missing or unclear
• A connection between the trial drug and the event cannot be reasonably excluded

no - unlikely
• There is a temporal relationship to the administration of the trial drug, but no causal connection between the drug and the event
• There is no temporal correlation with the administration of the trial drug (too early, too late)
• There is a reasonable causal relationship between another medication, a concomitant disease or other circumstances and the event
• there is a clear alternative explanation or implausibility (e.g., diagnosis of a cancer a few days after the first administration of the trial drug)

The following factors should be considered in the assessment:
• Temporal relationship (event occurs after administration of the trial drug)
• Response to discontinuation and reproduction of the trial drug
• Underlying concomitant diseases

• Concomitant medication or non-drug treatment
• Pharmacodynamics/pharmacokinetics of the trial drug

8.4.3 Procedures in case of adverse events

Procedures for the patient:
If the patient requires treatment as a result of the adverse event, it must be performed according to best available evidence to restore the patient's health.
Treatment of an AE should be documented as follows:
• No action taken
• Medication
• Surgical procedure
• Additional intensive medical care
• Other measures

Procedures in relation to the trial drug
The handling of the trial drug with regard to an AE must be documented as follows:
• Administration of trial drug interrupted - temporarily
• Trial drug discontinued - permanently
• Dose reduction
• No dose change
• Not applicable, as administration of trial drug had already been terminated or was never given.

8.4.4 Outcome of an adverse event

The outcome of an adverse event will be classified as follows:

- Resolved
- Resolved with sequelae
- Not resolved
- Fatal
- Unknown

8.5 Serious adverse event

According to GCP-V § 3 (30), a Serious Adverse Event (SAE) or a Serious Adverse Reaction (SAR) is any adverse event or adverse reaction that is fatal or life-threatening, requires hospitalisation or prolongation of the length of stay, results in permanent or serious disability or disability or invalidity, or results in a congenital anomaly or birth defect. An SAE may also be an AE which, according to the medical assessment of the assessing investigator/deputy/medical member of the trial group, fulfils a comparable criterion.

A serious adverse event must be documented as an AE in the eCRF and reported with the SAE sheet.

8.6 Reporting serious adverse events

Obligations for documentation and appropriate reporting of SAEs should be followed according to GCP-V § 12 (4) – (6) und § 13 (1) – (6) (30).

8.6.1 Obligations of the investigator/deputy

The trial centre should inform ZKS Jena, which is commissioned by the SpB, without delay (at the latest within 24 hours after recognition), using an SAE form, about the occurrence of a serious adverse event, with the exception of events that do not need to be reported immediately according to the study protocol.

The "SAE registration form" should be sent by fax to:

**ZKS Jena**<br>**Fax: +49 (0) 3641 9399946**

If further information on the SAE becomes available at a later date, it must also be reported to ZKS Jena without delay. The report from the trial centre will be checked for completeness and plausibility. If necessary, queries will be processed and tracked.

For all notifications, personal data must be pseudoanonymised prior to transmission using the subject ID (patient identification number of the participant). The primary report and all follow-up reports must be aggregated using the Subject ID.

.

8.6.2 Obligations of the SpB

For each SAE submitted by the trial centre, the SpB should arrange a secondary assessment by an experienced medical specialist with regard to the criteria "serious", "causal relationship" and "expectation". If a SUSAR (Suspected Unexpected Serious Adverse Reaction) is determined by the secondary assessment, the event should be reported to the PEI, ethics committee and investigators of ZKS Jena, including the report of the second assessor of the SpB.

A SUSAR is a suspected adverse effect that is both severe and unexpected. An unexpected adverse effect according to GCP-V § 3 (30) is an adverse effect that does not match the type and severity of the known information about the trial drug, i.e., the product information brochure of Albutein® 200 g/l or Plasbumin® 20, solution for infusion.

The SpB is responsible for continuously reviewing the benefit-risk assessment of the clinical trial. Occasions for the reassessment of benefit and risk as well as measures to protect against immediate danger should be reported in accordance with the law within the specified deadlines.

All responsibilities, information channels and deadlines are defined in the SAE Manual.

8.7 Preexisting diseases

See 8.4 concomitant diseases

8.8 Pregnancy

According to the valid product information brochure, embryo-foetal toxicity, mutagenic or carcinogenic potential has not been reported in relation to the trial drug. Nevertheless, women of childbearing age can only participate if there is a negative pregnancy test. Breastfeeding women are also excluded from participating in the clinical trial. Onset of pregnancy during the clinical trial is not expected to occur because of the severity of the underlying disease and the time-limited administration of albumin. Therefore, explicit

information about contraceptive measures and pregnancy tests during the course of the clinical trial will be waived.

8.9 Data and safety monitoring board - SMC

The data and safety monitoring board is an independent committee composed of a group of individuals (four independent physicians/scientists, one of them a statistician) with relevant experience. The main task of the Committee will be to monitor the safety and efficacy of the use of the trial drug during the clinical trial. This committee makes recommendations for the continuation, modification or termination of the clinical trial.

The SMC will receive all safety-related data and information every 6 months or year to conduct an independent review of the safety of the clinical trial. The results will be brought to the attention of the BOB and the leading ethics committee as part of the annual safety report, the DSUR, or as necessary. Responsibilities and details of the SMC are described in the corresponding manual for the members of the committee.

9. Documentation and data management

9.1 List of responsibilities

Every person responsible for documentation in the eCRF must be identifiable. A list of signatures and abbreviations for persons who are allowed to make entries in the eCRF (Signature and Responsibility Log) must be filed in the ISF and in the TMF. This list also identifies other individuals involved in the clinical trial with their names, signatures, and abbreviations, as well as their responsibilities and authority.

9.2 Documentation of screening

For all patients potentially eligible to participate in the clinical trial, inclusion and exclusion criteria will be reviewed. Documentation of these patients in the screening and enrolment log is required. For eligible patients, whether randomisation takes place or not must be documented. Reasons for non-participation of an eligible patient must be indicated (screening failure).

9.3 Patient identification list

A Patient Identification List will be kept at the trial centre, listing any patient who has consented to participate in the clinical trial.
In the patient identification list, the complete name, randomisation number and, if applicable, further identification data (e.g., date of birth, sex) will be recorded for each patient and assigned to a subject ID (patient identification number, pseudonym). The Subject ID is created using a special algorithm with the Subject ID Generator.
This list must be treated with absolute confidentiality, must not leave the trial centre and must be filed in the ISF. It is necessary that patients can be identified for later requests by the data management team.
The patient identification list remains in the trial centre and must be archived for at least 30 years after the end of the clinical trial.

A patient's participation in the clinical trial must be noted in the patient records along with other clinical trial-related information.

9.4 Investigator site file (ISF)

The ISF will keep the documents required for the clinical trial, and will provide an overview on the clinical trial at the respective trial centre. The ISF contains the essential documents, such as the trial protocol, patient information and consent form, approval of the competent BOB, approval of the responsible ethics committee (s), notification to the competent state authorities, investigators' CVs, list of responsibilities, trial-related correspondence, and other relevant documents. As part of the monitoring process, the ISF will be checked to ensure it is up-to-date and complete in accordance with the regulations. Each ISF must be archived for at least 30 years after the end of the clinical trial.

9.5 Case report form (CRF)

Source data according to the ICH-GCP E6 (29) in this clinical trial are all routine data and laboratory reports and any other documents required for the clinical trial. The vast majority of the data to be collected will be routinely collected from patients and entered directly into the regular patient records. The data relevant for the clinical trial are collected via RDE (Remote Data Entry). For this purpose, the data will be entered by the investigator or an authorised member of the trial group on an online workstation into special masks, which represent an electronic CRF. The data will be transferred directly to the trial database in ZKS Jena via the electronic CRF.

The CRF consists of individual sections, including inclusion and randomisation, visits during the course of the trial, AE/SAE, end of intervention and discontinuation of the clinical trial. The entries to be recorded in the relevant sections are specified when the CRF is created.
It is the responsibility of the investigators to ensure that all data collected during the clinical trial are entered correctly and completely in the database created specifically for this clinical trial.
Corrections in the eCRF may only be made by authorised persons or by the responsible investigator/deputy/medical member of the test group and must be justified. Corrections are recorded so that the old entry can still be retrieved. All data and corrections will be automatically logged with date, time and the person entering.
A paper-based CRF as a "copy" will be provided to the trial centres as part of the ISF.

9.6 Data handling and data management

Data collection serves a scientific purpose. The data will be generated in the participating trial centres and recorded via a web application on the servers of the ZKS of the Jena University Hospital using the study management software "OpenClinica®". The software complies with regulatory requirements (ICH-GCP, 21CFRPart11) (29, 30). The data will be recorded using an encrypted data connection (HTTPS) in input masks via a web browser. To ensure pseudonymised data analysis, each patient will be assigned a unique Subject ID (Patient Identification Number) as mentioned above.
For data management, the study management software "OpenClinica®" will be used. Verification of the accuracy of the data will be done by range, validity and consistency checks. Implausible or missing data will be requested from the test centre. Any change to the data, e.g., due to the incorporation of answered questions, will be documented via an automatic change tracking (audit trail) in the database. By using a hierarchical, role-based access concept, unauthorised access to the data will be impossible.

9.7 Data retention, archiving of documents

As a documentation centre, ZKS Jena will also be responsible for data storage. The backup of electronic data will occur regularly. The data storage facilities are located in a locked, central room, accessible only to system administrators.

9.7.1 Data retention obligation of the SpB

According to GCP-V § 13 (32), the SpB must ensure that the main clinical trial documents, including the data entry forms, are kept for <u>at least 30 years</u> after termination of the clinical trial. Other regulations for the storage of medical records remain unaffected.
All documents must be kept in a safe place and kept confidential. If necessary (e.g., because of legal regulations or after consultation with the SpB), the documents may be revoked beyond the abovementioned period. It is the responsibility of the SpB to inform the appropriate persons if the documents no longer need to be kept and how to dispose of them.

9.7.2 Data retention obligation of the trial centre

Records and documents related to the clinical trial or use of the trial drug, e.g., patient identification list, informed consent forms, lists of trial drug distribution, correspondence with the Ethics Committee, competent authorities, SpB, and other relevant documents must be kept in accordance with GCP-V § 13 (32) <u>for at least 30 years</u> (or longer, if required by law).
The medical records and other original data must be kept for the longest possible period of time allowed by the hospital, institution or private practice (according to the archiving period valid for the trial centres and according to local methods and premises), but not less than 30 years. The investigator must take precautions to prevent the accidental or premature destruction of these documents.

9.8 Privacy and privacy statement

This clinical trial will serve a scientific goal of considerable clinical relevance. To achieve this goal, it is necessary to collect and process medical data from individual patients. Data collection will take place in the involved trial centres. All collected medical data will be entered in the trial centres with the aid of a computer-aided online data collection system and transmitted directly to the documentation centre based at the ZKS of the Jena University Hospital. Name-related identification of individual patients by the documentation centre is not required at any time during the clinical trial. During data collection and data processing, therefore, all necessary measures will be taken at the earliest possible date in order to establish de facto anonymisation.
Transfer of patient-related medical data from the trial centres to the documentation centre will be carried out using a pseudonym. No features will be transferred that enable immediate identification of specific patients by the documentation centre. However, to conduct queries within the framework of the ongoing monitoring of the quality of documentation, it is necessary for the documentation centre to assign pseudonyms to specific trial centres.
In the case of revocation of consent, no further data will be collected. Data already collected will not be deleted.
The data entry, processing and evaluation carried out at ZKS Jena comply with the provisions of the Data Protection Act. Only employees of ZKS Jena will have access to all clinical trial data. These persons are sworn to secrecy. The data will be protected against unauthorised access.

9.8.1 Privacy statement

According to § 7 para. 2 no. 15 GCP-V (31), the concerned persons, in whom transmission of their pseudoanonymised data within the scope of the documentation and notification obligations under § 12 and § 13 GCP-V (31) is necessary, should be informed about the nature of the transmitted data. Persons who disagree with the disclosure of their pseudoanonymised data will not be included in the clinical trial.
The relevant data protection provisions (Thuringian Data Protection Act, Federal Data Protection Act, EU General Data Protection Regulation) must be complied with.
.

10. Quality assurance

10.1 Standardisation and validation

The responsible local laboratories of the trial centres must have a QM system and appropriate internal and external quality assurance measures. Successful participation in external quality assurance measures must be demonstrated by the submission of appropriate proficiency test certificates, accreditation certificates or similar documents. The laboratory tests required for the clinical trial must be GCLP-compliant and follow procedures established in the study manual.
For the required determination of the serum albumin concentrations at the respective trial centres, comparable CE-IVD certified methods should be used. The method equivalency of the device must be substantiated prior to the start of the clinical trial by providing appropriate information on the performance of the methods used locally, reference ranges, intra- and inter-assay variation, accuracy, etc.

10.2 Monitoring

According to ICH-GCP E6 (R2) (29), continuous monitoring of a clinical trial or its implementation is an indispensable quality assurance tool. The purpose of such monitoring is to ensure that the clinical trial has been conducted in accordance with the approved protocol and that the legal regulations and standards have been followed. The SpB entrusts ZKS Jena with the monitoring of the trial. The monitoring includes selection visits, initiation visits for training and briefing of the trial group prior to the start of recruitment or a central initiation, regular on-site visits as well as concluding visits for the correct closure of the trial centres. The monitoring will be carried out according to the standard operating instructions of ZKS Jena. Detailed information on the scope, procedure and content of the monitoring, as well as procedures for securing data quality and necessary measures in the event of deviations from the trial protocol will be described in a monitoring plan to be released by the SpB.
The investigators will provide the Monitor, for the purposes of monitoring the clinical trial, direct access to the original data and documents. The Monitor is required to treat all information confidentially and to protect the participants' fundamental right to integrity and privacy. The Monitor will report to the SpB in writing on the progress and results of his/her monitoring activities.

10.3 Audits and inspections

Within the scope of quality assurance, the SpB can have an independent audit conducted at any time in the participating institutions. In this case, the investigator or the participating institution will grant the auditor access to all documents necessary for the audit.

Inspections as part of the monitoring of ongoing or already completed clinical trials are carried out by the competent authority in accordance with § 64 (1) of the AMG (28). The inspection conducted by the competent authority shall be carried out in accordance with a written procedure and a pre-determined plan.
The SpB and all participating trial centres are committed to supporting inspections by competent authorities and, in this regard, to grant authorised persons access to the original data and documents.

11. Statistical methods and determination of sample size

11.1 Sample size

The 90-day mortality in the control arm is estimated to be about 50% (taking into account the possible use of albumin in the control group) (4, 7, own pilot study).
For the sample size estimation, a 15% reduction in 90-day mortality is assumed, i.e., an absolute reduction of 7.5 percentage points to 42.5% (relative risk 1.18). This limit was chosen because the relatively costly albumin therapy can only be justified if there is a sufficiently clear treatment effect. This assumed effect size has the potential to affect current standard of care and international treatment guidelines and lies between the observed reduction in 90-day mortality in patients with septic shock (13%) and the assumed effect size in the ALBIOS study (23). Following the recommendation of the reviewers of the DFG application of the ARISS study, the sample size was estimated taking into account a centre variability (probably about 50 centres) in mortality of patients in the control arm of the study between 40% and 60% and a risk reduction between 12% and 18% (coincidentally uniform).
A Mantel-Haenszel Chi² test at a two-tailed significance level of 0.05 with a power of 80% requires ***1412 patients to be analysed***, 706 per arm, to demonstrate such an effect.
This case number estimate can be considered conservative for the planned analysis (nonlinear mixed model taking into account random centre effects).
The case number estimation was performed using SAS 9.4 (SAS Institute Inc., Cary, NC, USA).

Dropout Rate:
Approximately 15%, through
• "loss to follow up" until day 90
• refusal to continue in the clinical trial by incapacitated patients who regain their capacity to consent, and
• failure to consent to the continuation of the clinical trial by the guardian or the legal representative within the respective time windows in patients who became participants after confirmation of the urgency of participation in the clinical trial by an independent consultant or other procedures, established in the trial centres to include patients who are aunable to provide informed consent (VISEP 10.5%). MaxSep 8.2%) (25, 26).
Assuming a dropout rate of 15%, ***1662 patients need to be randomised***.
.

11.2 Randomisation

Randomisation will be stratified according to:
- Serum lactate levels within 24 h before inclusion in the trial: ≤ 8 mmol/l vs. > 8 mmol/l
  and
- centre

in the treatment groups
- albumin group
  and
- control group without albumin

Albumin administration will be adjusted after the starting dose according to serum albumin concentrations. Therefore, blinding will not be possible.

Group assignment will be determined using a validated electronic tool (PaRANDies) after inclusion of the patient. Randomisation will be based on a randomisation list created in advance with nQuery Advisor 7.0.

11.3 Statistical methods

11.3.1 Definition of the analysis populations

Intention-to-Treat (ITT)
The intention-to-treat (ITT) population will include all patients enrolled in the study and randomised with at least one observation made after randomisation.
The primary efficacy analysis will be performed in the ITT population.
All variables collected will be analysed in the ITT population.
Per-Protocol (PP)
The Per-Protocol population will include all ITT patients who do not have major study plan deviations. A protocol deviation will be classified as "major" if it significantly affects the main target parameter (90-day mortality).
In particular, administration of albumin in the interest of patient safety outside the limits specified in the interventional group, albeit approved by the protocol, will always result in exclusion from the PP analysis.
As a sensitivity analysis, the primary efficacy analysis will be repeated in the PP population. If there are differences between the randomised and the actual treatment, an additional sensitivity "as-treated" analysis will be performed.
Details on trial protocol deviations will be described in the statistical analysis plan.
Listing-Only-Set (LOS)
Data from all patients not included in the ITT analysis can be listed as needed. The reasons for the exclusion will be recorded and analysed as far as possible.
Safety Analysis
The safety analysis will be carried out in the ITT group.

11.3.2 Data analysis

General methodology:
All collected data will be analysed using descriptive methods in the two treatment groups. This includes at least:
• the number of values collected and missing, mean, standard deviation, minimum, quartiles including median and maximum for continuous variables and
• frequency analyses for ordinal and categorical data.
• At baseline, the last available value will be used no later than the period up to 2 hours after randomisation, but before the start of albumin administration.

Baseline and population characteristics, demographics:
Demographic variables, SAPS II, APACHE II: descriptive analysis

Primary analysis:
The primary endpoint, "90-day mortality" (M90), will be calculated using a generalised mixed model with the random effects "centre" and "patient in centre" and the fixed effects SAPS II, SOFA and "baseline serum albumin value" as well as " treatment group "to the significance level Alpha = 0.05 analysed bilaterally.
The null hypothesis, M90 (control group) = M90 (albumin infusion), will be tested against the alternative M90 (control group) ≠ M90 (albumin infusion).
The primary analysis will be conducted in the ITT population.

Additional sensitivity analyses:
• Analysis in the PP population
• If more than 10% of M90 data are missing: multiple imputation analysis based on observed mortality rates, SOFA scores, ICU stay, and hospital stay.

Secondary endpoints:

• 28-day, 60-day, intensive care and hospital mortality: descriptive statistics, Kaplan-Meier estimates on mortality.
• Organ failure defined as increase in the daily recorded SOFA (day 1 to 28) from a value <2 to a value ≥ 2, individual number of organ failures in the period from day 1 to day 28: descriptive statistics, Kaplan-Meier estimates for the date of the individual first organ failure
• SOFA score: descriptive statistics
• Length of stay (after randomisation, until first discharge) in ICU, hospitalisation after randomisation (until first discharge to rehabilitation, transitional care, long-term care or home): descriptive statistics, Kaplan-Meier estimates, censored after drop-out and death.
• Ventilation-free days, vasopressor-free days until (first) discharge of ICU or maximum 28 days: descriptive statistics
• Total amount of fluid administration in the ICU (individual average amount of fluids given enterally or parentrally, including volume therapy) and total fluid balance in the ICU (individual average difference between daily fluid administration – urinary output and other fluid loss): descriptive statistics, especially quartiles.

All secondary endpoints, unless included in the primary analysis model, will be exploratively compared using appropriate parametric and nonparametric tests between treatment groups.

Health economics:
In the context of the ARISS study, a health economic cost-benefit analysis of volume replacement therapy of the ICU stay will be carried out up to a maximum of day 28. The direct costs of colloid, crystalloid and albumin administration will be recorded and the length of stay (first hospitalisation and stay since study participation) evaluated economically to assess whether albumin administration has an effect on hospital costs or not. At the patient level, DEALE (declining exponential approximation of life expectancy) (27) will be applied to calculate patient-specific life expectancy based on mortality rates in the general population and in the disease-specific population. The subsequent cost-effectiveness analysis will yield the cost per year of life for albumin administration. The robustness of the analysis will be checked with one-way and multi-way analyses as well as probabilistic sensitivity analysis.

Analysis of safety parameters:

Frequencies of AEs and SAEs will be analysed using frequency tables. In addition, frequencies of selected AEs (sepsis-related clinical events) will be analysed.

11.3.3 Subgroup analyses

The primary endpoint and selected secondary endpoints (SOFA, organ failure defined as SOFA organ failure score ≥ 2) will be described for the following subgroups:
Baseline lactate ≤ 8 mmol/L versus> 8 mmol/L
SOFA total and subscores, SAPS II, APACHE II: by baseline quartiles
Details of the statistical analyses will be described in the Statistical Analysis Plan.

11.3.4 Interim analysis

Not planned.

11.4 Presentation of results

The final statistical report will be based on the specifications and checklist of the CONSORT statement on the publication of randomised controlled trials in parallel group design (33) and on regulatory requirements.

12. Regulatory and administrative aspects

12.1 Regulations

The clinical trial will be conducted according to the current protocol, in agreement with the current versions of the German Law on Marketing Authorisation for Medicinal Products (AMG) (28), the ordinance on the Application of Good Clinical Practice in Conducting Clinical Trials with Medicinal Products (GCP-V) (31), the principles published in the ICH GCP Guideline (29), and the ethical principles set out in the Helsinki Declaration in 2008 for clinical trials as a recognised ethical basis (34).

12.2 Ethic committees

According to § 40 AMG (28), a clinical trial on a medicinal product in humans may only be started if the competent Ethics Commission (EC) has approved it in accordance with § 42 (1). For multicentre clinical trials that are carried out within the scope of the AMG in more than one trial centre, each further EC (involved EC) will receive a copy of the application and the documents from the SpB at the same time, according to state law for the respective investigator. Each investigator will receive from the applicant a copy of the approval of the IC for the ISF.

12.3 Competent authorities

12.3.1 Federal authority

For this clinical trial under the AMG, the Federal Institute for Vaccines and Biomedical Drugs (Paul-Ehrlich-Institut (PEI) in Langen) is the competent higher federal authority (BOB).
According to § 40 AMG (28), the clinical testing of a medicinal product in humans may only be started if the competent higher federal authority has approved it in accordance with § 42 (3).
Each investigator will receive from the applicant a copy of the authorization for the ISF.

12.3.2 State authorities

Pursuant to § 67 AMG (28), each registered investigator/deputy has a general obligation to notify the competent authority. Pursuant to Section 12 GCP-V (31), the investigator may entrust the sponsor with the execution of the notification to the competent authority and must document this.
In each case, the government authorities or the competent authorities in the countries in which a trial centre is located are responsible for the clinical trial. When the sponsorship is taken over by the Friedrich Schiller University Jena or the Jena trial Centre, notification will be made to the Thüringer Landesamt für Verbraucherschutz (TLV) in Bad Langensalza. The respective state report must be filed in the respective trial centre folder.
.

12.4 Subsequent changes in the trial protocol - amendments

According to § 10 (1) GCP-V (31), changes in the trial protocol requiring approval are to be applied for by the SpB and only after approval by the responsible EC, insofar as they relate to the information and documents pursuant to § 7 (2), (3) or (3a) and if they have been approved by the responsible BOB, insofar as they relate to the information and documents pursuant to § 7 (2) or (4). This excludes amendments that are necessary in order to avert immediate danger to the concerned persons (§ 11 GCP-V, (31)), which must be implemented immediately. The SpB will immediately inform the responsible BOB and the responsible EC about these new circumstances. A favourable evaluation should be obtained from the responsible EC and approval should be requested from the responsible BOB. The

application must be reasoned. Even changes that are only subject to approval by the responsible EC are to be reported to the responsible BOB. To ensure largely comparable conditions in all trial centres and in the interest of a flawless data evaluation, there is no intention to perform any amendment or changes to the trial conditions agreed upon in the trial protocol (i.e., study design, course of study or evaluation procedure).

12.5 Registration

Registration of the clinical trial will be done before randomisation of the first patient in the WHO-recognised German Register of Clinical Trials (DRKS, www.drks.de). In addition, the clinical trial will be registered at clinicaltrials.gov. The registry entries will be maintained continuously.

12.6 Insurance

According to § 40 AMG (29), insurance must be taken out by the SpB for all patients participating in the trial.
Subject insurance will be concluded for all patients included according to § 40 AMG in the context of the proband year contract of the hospital of Friedrich-Schiller-University Jena with the HDI Gerling Industrie Versicherung AG, 30659 Hannover. The seat, policy number, telephone number and fax number of the insurance company will be included in the patient information form. Travel accident insurance will not be taken out. Patients will be informed about their rights and obligations in connection with the insurance. Each participant in the clinical trial will receive the conditions of insurance in writing.

12.7 Funding

The German Research Foundation (DFG) will fund the clinical trial within the framework of the program "clinical studies", grant number DFG SA 2167 / 3-1, project number 328809707.
The Department of Anaesthesiology and Intensive Therapy of the University Hospital, Jena will take over the financing of the remaining duration of the clinical trial after the DFG funding period has expired.
The trial drug for treatment of the albumin group will be provided free of charge by GRIFOLS SA (Jesus y Maria 6, 08822 Barcelona).

12.8 Reports of the study results

All information concerning this clinical trial must be treated confidentially. According to AMG § 42 b (29), the SPB is required to publish the results of clinical trials within certain deadlines and in compliance with certain requirements.
The SpB is responsible for the preparation of the final report.

12.9 Publications

The aim will be to publish the results of this clinical trial in a prestigious international medical journal.
Authorship will be governed by the guidelines of the New England Journal of Medicine (http://www.icmje.org/). It will be based on the following criteria:

- Substantial contribution to the conception or design of the manuscript or to the collection, analysis and interpretation of data for the manuscript
- Substantial intellectual contribution to manuscript preparation
- Final assessment or approval of the manuscript version to be published
- Declaration of responsibility for all aspects of the work to ensure that issues related to the accuracy or integrity of part of the work are adequately investigated and answered

Authors should meet all four criteria. The Acknowledgment section of the publication should list the names of all the key personnel involved in the clinical trial in the trial sites. The trial

centres are entitled to scientifically use the data generated in this clinical trial. According to the contractual agreements or after consultation with the Executive Committee, the trial centres can use these data to process their own scientific questions and publish the results under their own names.
Publication or presentation of the results requires prior comment and approval from the SpB. All publications will ensure privacy rules are met for all patients.

13. Literature

1. Singer M, Deutschman CS, Seymour CW, et al. The Third International Consensus Definitions for Sepsis and Septic Shock (Sepsis-3). JAMA. 2016 Feb 23;315 (8):801-10.
2. Calandra T and Cohen J. The international sepsis forum consensus conference on definitions of infection in the intensive care unit. Crit Care Med 2005; Jul;33(7):1538-48
3. Martin GS, Mannino DM, Eaton S et al. The epidemiology of sepsis in the United States from 1979 through 2000. N Engl J Med 2003, 348: 1546-1554.
4. Brunkhorst FM, Hagel S, Ludewig K et al. Direct costs related to severe sepsis in Germany: an update. Infection 2011; A S144-145.
5. Fleischmann C, Thomas-Rüddel DO, Hartmann M et al. Fallzahlen und Sterblichkeitsraten von Sepsis-Patienten im Krankenhaus. Deutsches Ärzteblatt 2016, 113 10:159-166.
6. Vincent JL, Sakr Y, Reinhart K, et al. Is albumin administration in the acutely ill associated with increased mortality? Results of the SOAP study. Crit Care. 2005; 9(6): R745-54.
7. Vincent JL, Marshall JC, Namendys-Silva SA, et al. Assessment of the worldwide burden of critical illness: the Intensive Care Over Nations (ICON) audit. Lancet Respir Med. 2014; 2(5):380-6.
8. SepNet Critical Care Trials Group. Incidence of severe sepsis and septic shock in German intensive care units: the prospective, multicentre INSEP study. Intensive Care Med. 2016; 42(12):1980-1989.
9. Holst LB, Haase N, Wetterslev J, et al.; TRISS Trial Group.; Scandinavian Critical Care Trials Group. Lower versus higher hemoglobin threshold for transfusion in septic shock. N Engl J Med. 2014 Oct 9; 371(15):1381-91.
10. The ARISE Investigators and the ANZICS Clinical Trials Group. Goal-Directed Resuscitation for Patients with Early Septic Shock. N Engl J Med 2014; 371:1496-1506.
11. Mouncey PR, Osborn TM, Power GS, et al. for the ProMISe Trial Investigators. Trial of Early, Goal-Directed Resuscitation for Septic Shock. N Engl J Med 2015; 372:1301-1311.
12. The ProCESS Investigators. A Randomized Trial of Protocol-Based Care for Early Septic Shock. N Engl J Med 2014; 370:1683-1693.
13. . Fanali G, di Masi A, Trezza V, et al. Human serum albumin: from bench to bedside. Mol Aspects Med. 2012; 33(3):209-90.
14. O'Brien AJ, Fullerton JN, Massey KA, et al. Immunosuppression in acutely decompensated cirrhosis is mediated by prostaglandin E2. Nat Med 2014; 20(5):518-23.
15. Taverna M, Marie AL, Mira JP, et al. Specific antioxidant properties of human serum albumin. Ann Intensive Care 2013; 15; 3(1):4.
16. Walley KR, McDonald TE, Wang Y,et al. Albumin resuscitation increases cardiomyocyte contractility and decreases nitric oxide synthase II expression in rat endotoxemia. Crit Care Med. 2003 Jan; 31(1):187-94.
17. Tokunaga C, Bateman RM, Boyd J, et al. Albumin resuscitation improves ventricular contractility and myocardial tissue oxygenation in rat endotoxemia. Crit Care Med 2007 May; 35(5):1341-7.
18. Cochrane Injuries Group Albumin Reviewers. BMJ. Human albumin administration in critically ill patients: systematic review of randomised controlled trials. 1998 Jul 25; 317(7153):235-40.
19. Wilkes and Navickis. Patient survival after human albumin administration. A meta-analysis of randomized, controlled trials. Ann Intern Med 2001; 135:149-64.

20. Vincent JL1, Navickis RJ, Wilkes MM. Morbidity in hospitalized patients receiving human albumin: a meta-analysis of randomized, controlled trials. Crit Care Med. 2004 Oct; 32(10):2029-38.
21. Dubois MJ, Orellana-Jimenez C, Melot C et al. Albumin administration improves organ function in critically ill hypoalbuminemic patients: A prospective, randomized, controlled study. Crit Care Med. 2006; 34(10): 2536-40.
22. Finfer S1, Bellomo R, Boyce N,et al. A comparison of albumin and saline for fluid resuscitation in the intensive care unit. N Engl J Med. 2004 27; 350(22):2247-56.
23. SAFE Study Investigators. Impact of albumin compared to saline on organ function and mortality of patients with severe sepsis. Intensive Care Med. 2011 Jan; 37(1):86-96.
24. Caironi P, Tognoni G, Masson S, et al. ALBIOS Study Investigators. Albumin replacement in patients with severe sepsis or septic shock. N Engl J Med. 2014. 10; 370(15):1412-21.
25. Rhodes A, Evans LE, Alhazzani W, et al. Surviving Sepsis Campaign: International Guidelines for Management of Sepsis and Septic Shock: Intensive Care Med. 2017 Mar; 43(3):304-377.
26. Brunkhorst FM, Engel C, Reinhart K et al. for the German Competence Network Sepsis (SepNet): A Trial of Intensive Insulin Therapy and Pentastarch Resuscitation in Severe Sepsis. N Engl J Med 2008; 358(2):125-39.
27. Brunkhorst FM, Oppert M, Reinhart K, et al. Effect of empirical treatment with moxifloxacin and meropenem vs. meropenem on sepsis-related organ dysfunction in patients with severe sepsis: a randomized trial. JAMA. 2012 13; 307(22):2390-9.
28. Barnett AG, Beyersmann J, Allignol A, et al. The time-dependent bias and its effect on extra length of stay due to nosocomial infection. Value in health: the journal of the International Society for Pharmacoeconomics and Outcomes Research. 2011; 14(2):381-6.
29. Gesetz über den Verkehr mit Arzneimitteln (Arzneimittelgesetz - AMG): Arzneimittelgesetz in der Fassung der Bekanntmachung vom 12. Dezember 2005 (BGBl. I S. 3394), das zuletzt durch Artikel 1 des Gesetzes vom 18. Juli 2017 (BGBl. I S. 2757) geändert worden ist.
30. ICH Topic E 6 (R2) Guideline for Good Clinical Practice (CHMP/ICH/135/1995). 2016, European Medicines Agency, London.
31. https://www.accessdata.fda.gov/scripts/cdrh/cfdocs/cfcfr/CFRSearch.cfm?CFRPart=11
32. Verordnung über die Anwendung der Guten klinischen Praxis bei der Durchführung von klinischen Prüfungen mit Arzneimitteln zur Anwendung am Menschen (GCP-Verordnung, GCP-V) vom 9.8.2004 2004, BGBl. I 2004 S. 2081 zuletzt durch Artikel 8 des Gesetzes vom 19. Oktober 2012 (BGBl. I S. 2192) geändert.
33. Schulz, K.F., D.G. Altman, and D. Moher, CONSORT 2010 statement: updated guidelines for reporting parallel group randomised trials. BMJ 340: p. c332.
34. World Medical Association, Declaration of Helsinki. Ethical Principles for Medical Research Involving Human Subjects 2008, 59. WORLD MEDICAL ASSOCIATION General Assembly: Seoul, Südkorea.

14. Appendix

14.1 SOFA Score

Organ dysfunction is defined according to the SOFA score variables. The worst value of the parameter each day will be used to calculate the score. The parameters of the SOFA score are determined until day 28 exclusively in the intensive care unit and are calculated from the sum of the evaluation points of the individual organ systems listed below. The parameters for calculating the SOFA subscore are also part of the CRF. The score points of each organ system range from 0 to 4. The SOFA score is calculated in the data management center (ZKS Jena). The subscores of the SOFA score are determined as follows:

Cardiovascular System

Mean arterial pressure (MAP) and the need for catecholamine determine the assigned points. The lowest MAP value of the past 24 hours is part of the CRF. The highest catecholamine dosage> 1 h of the past 24 hours is part of the individual variables in the CRF.

Score	*Cardiovascular status*
0	MAP ≥ 70 and no vasopressors
1	MAP < 70 and no vasopressors
2	Dopamine ≤5 µg/kg/min oder Dobutamine (and dose)
3	Dopamine >5 – ≤15 µg/kg/min **or** (nor)adrenalin ≤0.1 µg/kg/min
4	Dopamine>15 µg/kg/min **or** (nor)adrenalin >0.1 µg/kg/min

Pulmonary system

The PaO_2/FiO_2 ratio determines this sub-score. If the blood gas analysis is not available for the day or the patient is no longer intubated, but relies on oxygen therapy, conversion tables will be used.

Score	*PaO_2/FiO_2*
0	> 400 mmHg (> 53.2 kPa)
1	301–400 mmHg (39.9–53.1 kPa)
2	201–300 mmHg (26.6–39.8 kPa)
3	101–200 mmHg (13.3–26.5 kPa)
4	≤ 100 mmHg (< 13.3 kPa)

Coagulation system:

The platelet count determines this sub-score. The lowest platelet count of the past 24 hours is part of the CRF variables.

Score	*Platelet count*
0	$>150 \times 10^3/mm^3$
1	$100\text{-}149 \times 10^3/mm^3$
2	$50–99 \times 10^3/mm^3$
3	$20–49 \times 10^3/mm^3$
4	$<20 \times 10^3/mm^3$

Renal system:

Serum creatinine and urinary output determine this sub-score. The worst value is part of the CRF variables.

**Score**	Serum creatinine and urinary output
0	<1.2 mg/dl (<110 µmol/l)
1	1.2–1.9 mg/dl (110 –170 µmol/l)
2	2.0–3.4 mg/dl (171– 299 µmol/l)
3	3.5–4.9 mg/dl (300–440 µmol/l) **or** urinary output <500 ml/24h
4	≥5,0 mg/dl (≥ 441 µmol/l) **or** urinary output <200 ml/24h

Hepatic system:
Total bilirubin determines this value. The worst value is part of the CRF variables.

Score	***Total bilirubin***
0	<1.2 mg/dl (< 20 µmol/l)
1	1.2–1.9 mg/dl (20–32 µmol/l)
2	2.0–5.9 mg/dl (33–101 µmol/l)
3	6.0–11.9 mg/dl (102–204 µmol/l)
4	≥12 mg/dl (≥205 µmol/l)

Central nervous system (CNS):
The CNS is assessed by the Glasgow Coma Scale (GCS). If the patient is sedated, the assessment of conscious level is based on the observer's assumption that the patient is not sedated. In addition to this esteemed GCS, the actual GCS should also be documented. The worst estimated and worst actual GCS is part of the CRF variables.

Score	***Glasgow Coma Scale***
0	15
1	13–14
2	10–12
3	6–9
4	≤ 5

Glasgow Coma Scale
The Glasgow Coma Scale is a neurological assessment scoring system composed of three individual assessments, which are summed up below.

	Score		***Score***		***Score***
**Eye openning**		**Best motoric response**		**Beste verbal Response**	
Spontaneous	4	Obey commands	6		
On demand	3	Painful stimulation		Response to verbal	
Painful stimulation	2	localisation	5	contact	
None	1	flexion	4	oriented	5
		Abnormal flexion	3	confused	4
		Extention (decerebrate)	2	Words	3
		None	1	Sounds	2
				None	1

Refernce: Vincent JL, Moreno R, Takala J, et al. The SOFA (Sepsis-related Organ Failure Assessment) score to describe organ dysfunction/failure. Intensive Care Med 1996;22(7):707–710.

14.2 Apache II Score

Parameter/points	4	3	2	1	0	1	2	3	4
Core temperature	≥41	39–40.9		38.5–38.9	36–38.4	34–35.9	32–33.9	30–31.9	≤29.9
Mean art. pressure (mmHg)	≥160	130–159	110–129		70–109		50–69		≤49
Heart rate (/min)	≥180	140–179	110–139		70–109		55–69	40–54	≤39
Respiratory rate (/min)<br>*Spontaneous or ventilated*	≥50	35–49		25–34	12–24	10–11	6–9		≤5
Oxygenation (mmHg)<br>a) FiO_2≥0,5: $AaDO_2$<br>b) FiO_2<0,5: PaO_2	≥500	350–499	200–349		<200<br>>70	61–70		55–60	<55
Arterial pH	≥7.7	7.6–7.69		7.5–7.59	7.33–7.49		7.25–7.32	7.15–7.24	<7.15
Natrium (mmol/l)	≥180	160–179	155–159	150–154	130–149		120–129	110–119	<110
Kalium (mmol/l)	≥7	6–6.9		5.5–5.9	3.5–5.4	3–3.4	2.5–2.9		<2.5
Creatinine (mg/dl)[1]	≥3,5	2–3.4	1.5–1.9		0.6–1.4		<0.6		
Hematocrite (%)	≥60		50–59.9	46–49.9	30–45.9		20–29.9		<20
Leucocytes ($10^3/mm^3$)	≥40		20–39.9	15–19.9	3–14.9		1–2.9		<1
Glasgow Coma Scale [2]	Score=15 minus GCS								
Venous HCO_3 (mmol/l)[3]	≥52	41–51.9		32–40.9	22–31.9		18–21.9	15–17.9	<15

[1] In case of acute renal failure ×2
[2] see page 58
[3] only when arterial blood gases are missing

- The following points are assigned according to age: <=42 years: 0; 45 – 54 years: 2; 55 – 64 years: 3; 65 – 74 Jahre: 5; >=75 years: 6

- The following data about chronic diseases are collected (yes/no):

Liver: Cirrhosis (biopsy) and proven portal hypertension or gastrointestinal haemorrhage caused by portal hypertension or previous episodes of liver failure, encephalopathy, coma

Cardiovascular: NYHA IV

Respiratory: Chronic, restrictive, obstructive or vascular diseases that cause serious disability (for example, inability to climb stairs or do household chores); proven chronic hypoxia, hypercapnia, secondary polycythemia, severe pulmonary hypertension (> 40 mmHg), need for ventilation

Renal: Need for chronic dialysis

Immunsuppresive: Therapies that reduce resistance to infections (e.g., immune suppression, chemotherapy, radiation, long-term treatment (up to 30 days prior to hospitalization), or treatment with high-dose steroids (> 16mg / kg for 5 days)); the presence of a disease that has progressed so far that the immune system is severely impaired (e.g. leukemia, lymphoma, AIDS)

- 0 points, if the answer to all five questions is "no"
- 2 points, if the answer to at least one question is "yes" and the patient is in an elective post-operative state
- 5 points, if the answer to at least one question is "yes" and the patient is in a post-operative emergency or has not undergone surgery

Reference: Knaus WA, Draper EA, Wagner DP, Zimmerman JE. APACHE II: a severity of disease classification system. Crit Care Med 1985;13(10):818–829.

14.3 New Simplified Acute Physiology Score (SAPS II)

Parameter	***Befund***	***Punktwert***
Age (years)	<40	0
	40–59	7
	60–69	12
	70–74	15
	75–79	16
	≥80	18
Heart rate (/min)	<40	11
	40–69	2
	70-119	0
	120–159	4
	≥160	7
Systolic blood pressure (mmHg)	<70	13
	70–99	5
	100–199	0
	≥200	2
Körpertemperatur (°C)	<39	0
	≥39	3
PaO_2/FiO_2 (bei mechanical ventilation or CPAP) (mmHg)	<100	11
	100–199	9
	≥200	6
Urine output (l/24h)	<0.5	11
	0.5–0.999	4
	≥1.0	0
Urea (mg/dl) [mmol/l]	<28 [4,6]	0
	≥84 [13,9]	10
Leucocytes ($10^3/mm^3$)	<1.0	12
	1.0–19.9	0
	≥20	3
Kalium (mmol/l)	<3.0	3
	3.0–4.9	0
	≥5.0	3
Natrium (mmol/l)	<125	5
	125–144	0
	≥145	1
Bicarbonate (mmol/l)	<15	6
	15–19	3
	≥20	0
Bilirubin (mg/dl) [µmol/l]	<4.0 (68.4)	0
	4.0–5.9 (68.4–102.5)	4
	≥6.0 (102.5)	9
Glasgow Coma Score (s page 58)	<6	26
	6–8	13
	9–10	7
Chronic diseases	Metastatic cancer	9
	Hematologic malignancy	10
	AIDS	17
Type of admission	Planned OP	0
	Medical admission	6
	Emergency surgery	8

Reference: Le Gall JR, Lemeshow S, Saulnier F. A new Simplified Acute Physiology Score (SAPS II) based on a European/North American multicentre study. JAMA 1993;270(24):2957–2963.

14.4 Conversion tables

PaO_2/FiO_2 Ratio

The following conversion table is used when blood gas analyzes are not available to determine PaO2 / FiO2:

O_2 Saturation conversion table	
SO_2 (%)	estimated PaO_2 (mmHg)
80	44
81	45
82	46
83	47
84	49
85	50
86	52
87	53
88	55
89	57
90	60
91	62
92	65
93	69
94	73
95	79
96	86
97	96
98	112
99	145

FiO_2 on oxygen therapy

Method	***O_2 Flow (l/min)***	***estimated FiO_2 (%)***
Nasal cannula	1<br>2<br>3<br>4<br>5<br>6	24<br>28<br>32<br>36<br>40<br>44
Nasopharyngeal catheter	4<br>5<br>6	40<br>50<br>60
Face mask	5<br>6–7<br>7–8	40<br>50<br>60
Face mask with reservoir	6<br>7<br>8<br>9<br>10	60<br>70<br>80<br>90<br>95
