# Supplementary material for: Albumin Replacement Therapy in Septic Shock: A Randomized Clinical Trial
Source: JAMA Netw Open. 2026 Feb 19;9(2):e2559297. doi: 10.1001/jamanetworkopen.2025.59297 (PMC12921518; doi:10.1001/jamanetworkopen.2025.59297)
Supplement: Supplement 2. — eTable 1. Sepsis-related clinical events, possible adverse effects of the trial drug, and classification of severity of adverse events (AEs) eTable 2. Patient inclusion according to site eTable 3. Types of infection in the two study groups at randomization eTable 4. Mode of acquisition of infection in the two study groups eTable 5. Albumin levels of patients by study time frame eTable 6. Protocol deviations in the two study groups eTable 7. Physiologic and oxygenation parameters, fluid administration, vasopressor therapy, and procedures in the two groups 6 hours after randomization eTable 8. Mortality rates in the two study groups according to baseline lactate, baseline SOFA, SAPS II, and APACHE II eTable 9. Frequency of AEs according in the two study groups eTable 10. Frequency of sepsis-related events in the two study groups eFigure 1. Study flow chart of interventions eFigure 2. Box plot showing mean arterial pressure in the two groups measured at screening, 6 hours after randomization, and then daily for 28 days in the ICU eFigure 3. Box plot showing the mean daily fluid intake in the two groups at screening, 6 hours after randomization, and then daily for 28 days in the ICU eFigure 4. Box plot showing the mean daily fluid output (excluding perspiration) in the two groups at screening, 6 hours after randomization, and then daily for 28 days in the ICU [file jamanetwopen-e2559297-s002.pdf]

Supplemental Online Content

Sakr Y, Nierhaus A, Schumacher U, et al. Albumin replacement therapy in septic shock: a randomized clinical trial. *JAMA Netw Open*. 2026;9(2):e2559297. doi:10.1001/jamanetworkopen.2025.59297

eTable 1. Sepsis-related clinical events, possible adverse effects of the trial drug, and classification of severity of adverse events (AEs)

eTable 2. Patient inclusion according to site

eTable 3. Types of infection in the two study groups at randomization

eTable 4. Mode of acquisition of infection in the two study groups

eTable 5. Albumin levels of patients by study time frame

eTable 6. Protocol deviations in the two study groups

eTable 7. Physiologic and oxygenation parameters, fluid administration, vasopressor therapy, and procedures in the two groups 6 hours after randomization

eTable 8. Mortality rates in the two study groups according to baseline lactate, baseline SOFA, SAPS II, and APACHE II

eTable 9. Frequency of AEs according in the two study groups

eTable 10. Frequency of sepsis-related events in the two study groups

eFigure 1. Study flow chart of interventions

eFigure 2. Box plot showing mean arterial pressure in the two groups measured at screening, 6 hours after randomization, and then daily for 28 days in the ICU

eFigure 3. Box plot showing the mean daily fluid intake in the two groups at screening, 6 hours after randomization, and then daily for 28 days in the ICU

eFigure 4. Box plot showing the mean daily fluid output (excluding perspiration) in the two groups at screening, 6 hours after randomization, and then daily for 28 days in the ICU

This supplemental material has been provided by the authors to give readers additional information about their work.

© 2026 Sakr Y et al. *JAMA Network Open*. This is an open access article distributed under the terms of the CC-BY-NC-ND License, which does not permit alteration or commercial use, including those for text and data mining, AI training, and similar technologies.

eTable 1. Sepsis-related clinical events, possible adverse effects of the trial drug, and classification of severity of adverse events

- ***Sepsis-related clinical events***
  - Death caused by severe sepsis or septic shock
  - Cardiovascular event requiring the administration of vasoactive substances
  - Respiratory event: e.g., decrease in PaO2/FiO2 ratio, hypoxia, ARDS, acute pulmonary dysfunction, and mechanical ventilation
  - Hepatic event: e.g., liver failure or liver dysfunction
  - Renal event: e.g., kidney failure, renal insufficiency
  - Hematological event: e.g., coagulopathy, thrombocytopenia, thrombocytosis
  - Neurological event: e.g., delirium, confusion

- ***Possible adverse effects of the trial drug***
  - Flush
  - Urticaria
  - Fever
  - Nausea
  - Anaphylactic shock
  - Hypervolemia
  - Pulmonary edema
  - Transmission of infection

- ***Classification of adverse effects according to severity***
  - Mild: a clinical symptom or sign that is well/easily tolerated and usually requires no intervention.
  - Moderate: clinical symptom or sign sufficient to interfere with normal/daily activity, intervention may be required.
  - Severe: a clinical symptom or sign that results in severe disability, inability to work or inability to perform everyday activities or daily activities/work not possible, treatment or intervention usually required.

ARDS: acute respiratory distress syndrome

eTable 2. Patient inclusion according to site

		Serum lactate					
		<= 8 mmol/l (<= 72 mg/dl)		> 8 mmol/l (> 72 mg/dl)		Total	
Site and lead investigator	Study group	N	%	N	%	N	%
01 – Jena Sakr	Albumin	16	4.7	3	2.9	19	4.3
	Control without albumin	15	4.5	4	3.9	19	4.3
02 – Jena Schulze	Albumin	1	0.3	0	0.0	1	0.2
	Control without albumin	2	0.6	0	0.0	2	0.5
03 – Leipzig Fichtner	Albumin	10	3.0	6	5.8	16	3.6
	Control without albumin	8	2.4	7	6.8	15	3.4
04 – Leipzig Petros	Albumin	14	4.2	5	4.9	19	4.3
	Control without albumin	15	4.5	4	3.9	19	4.3
06 – Hamburg Nierhaus	Albumin	40	11.9	8	7.8	48	10.9
	Control without albumin	43	12.8	4	3.9	47	10.7
08 – Kiel Eimer	Albumin	10	3.0	3	2.9	13	3.0

© 2026 Sakr Y et al. *JAMA Network Open*. This is an open access article distributed under the terms of the CC-BY-NC-ND License, which does not permit alteration or commercial use, including those for text and data mining, AI training, and similar technologies.

		Serum lactate					
		<= 8 mmol/l (<= 72 mg/dl)		> 8 mmol/l (> 72 mg/dl)		Total	
Site and lead investigator	Study group	N	%	N	%	N	%
	Control without albumin	9	2.7	3	2.9	12	2.7
09 – Herne Roghmann	Albumin	2	0.6	0	0.0	2	0.5
	Control without albumin	0	0.0	0	0.0	0	0.0
10 – Augsburg Jaschinski	Albumin	13	3.9	2	1.9	15	3.4
	Control without albumin	14	4.2	2	1.9	16	3.6
11 – Göttingen Mörer	Albumin	3	0.9	1	1.0	4	0.9
	Control without albumin	3	0.9	1	1.0	4	0.9
12 – München Frank	Albumin	1	0.3	0	0.0	1	0.2
	Control without albumin	0	0.0	0	0.0	0	0.0
13 – München Kapfer	Albumin	1	0.3	0	0.0	1	0.2
	Control without albumin	0	0.0	0	0.0	0	0.0
14 – Freiburg Utzolino	Albumin	26	7.7	5	4.9	31	7.0
	Control without albumin	24	7.1	4	3.9	28	6.4
15 – Greifswald Scheer	Albumin	4	1.2	6	5.8	10	2.3
	Control without albumin	4	1.2	7	6.8	11	2.5
16 – Berlin Lehmke	Albumin	0	0.0	0	0.0	0	0.0
	Control without albumin	1	0.3	0	0.0	1	0.2
17 – Magdeburg Tanev	Albumin	7	2.1	3	2.9	10	2.3
	Control without albumin	6	1.8	4	3.9	10	2.3
18 – Magdeburg Jacob	Albumin	2	0.6	1	1.0	3	0.7
	Control without albumin	2	0.6	0	0.0	2	0.5
20 – Bonn Putensen	Albumin	9	2.7	5	4.9	14	3.2
	Control without albumin	8	2.4	6	5.8	14	3.2
21 – Köln Fiedler	Albumin	4	1.2	2	1.9	6	1.4
	Control without albumin	5	1.5	2	1.9	7	1.6
23 – Erlangen Wehrfritz	Albumin	0	0.0	0	0.0	0	0.0
	Control without albumin	0	0.0	1	1.0	1	0.2
25 – Bad Saarow Kreienbühl	Albumin	6	1.8	0	0.0	6	1.4
	Control without albumin	4	1.2	3	2.9	7	1.6
26 - Regensburg Schlitt	Albumin	1	0.3	0	0.0	1	0.2
	Control without albumin	0	0.0	1	1.0	1	0.2
27 - Homburg Meiser	Albumin	1	0.3	0	0.0	1	0.2
	Control without albumin	1	0.3	0	0.0	1	0.2
28 - Herford Kähler	Albumin	1	0.3	0	0.0	1	0.2
	Control without albumin	1	0.3	0	0.0	1	0.2

eTable 3. Types of infection in the two study groups at randomization

		Albumin group, n (%) (N = 222)	Control group, n (%) (N = 218)
Intraabdominal			
	Microbiologically proven	40 (18.0)	39 (17.9)
	Definite	29 (13.1)	24 (11.0)
	Possible	3 (1.4)	6 (2.8)
	Probable	10 (4.5)	9 (4.1)
	Total	82 (36.9)	78 (35.8)
Pneumonia			
	Microbiologically proven	17 (7.7)	25 (11.5)
	Definite	22 (9.9)	17 (7.8)
	Possible	9 (4.1)	5 (2.3)

© 2026 Sakr Y et al. *JAMA Network Open*. This is an open access article distributed under the terms of the CC-BY-NC-ND License, which does not permit alteration or commercial use, including those for text and data mining, AI training, and similar technologies.

		Albumin group, n (%) (N = 222)	Control group, n (%) (N = 218)
	Probable	4 (1.8)	8 (3.7)
	Unknown	-	2 (0.9)
	Total	52 (23.4)	57 (26.1)
Bacteremia			
	Microbiologically proven	30 (13.5)	30 (13.8)
	Definite	1 (0.5)	1 (0.5)
	Probable	2 (0.9)	-
	Total	33 (14.9)	31 (14.2)
Urogenital			
	Microbiologically proven	24 (10.8)	16 (7.3)
	Definite	5 (2.3)	1 (0.5)
	Possible	2 (0.9)	1 (0.5)
	Probable	3 (1.4)	3 (1.4)
	Unknown	1 (0.5)	-
	Total	35 (15.8)	21 (9.6)
Gastrointestinal			
	Microbiologically proven	9 (4.1)	3 (1.4)
	Definite	2 (0.9)	9 (4.1)
	Possible	3 (1.4)	2 (0.9)
	Probable	2 (0.9)	4 (1.8)
	Unknown	1 (0.5)	-
	Total	17 (7.7)	18 (8.3)
Bones, soft tissue			
	Microbiologically proven	10 (4.5)	7 (3.2)
	Definite	5 (2.3)	2 (0.9)
	Probable	3 (1.4)	-
	Total	18 (8.1)	9 (4.1)
Thoracic infections	Microbiologically proven	3 (1.4)	4 (1.8)
	Definite	4 (1.8)	2 (0.9)
	Total	7 (3.2)	6 (2.8)
Surgical wound			
	Microbiologically proven	2 (0.9)	7 (3.2)
	Definite	1 (0.5)	1 (0.5)
	Total	3 (1.4)	8 (3.7)
Cardiovascular			
	Microbiologically proven	-	1 (0.5)
	Definite	-	1 (0.5)
	Possible	1 (0.5)	1 (0.5)
	Probable	1 (0.5)	-
	Total	2 (0.9)	3 (1.4)
Renal			
	Microbiologically proven	3 (1.4)	4 (1.8)
	Definite	1 (0.5)	-
	Possible	-	1 (0.5)
	Total	4 (1.8)	5 (2.3)
Skin			
	Microbiologically proven	1 (0.5)	5 (2.3)
Central nervous system			
	Microbiologically proven	-	1 (0.5)
Catheter-related			
	Microbiologically proven		4 (1.8)
	Definite	1 (0.5)	-
	Total	1 (0.5)	4 (1.8)

© 2026 Sakr Y et al. *JAMA Network Open*. This is an open access article distributed under the terms of the CC-BY-NC-ND License, which does not permit alteration or commercial use, including those for text and data mining, AI training, and similar technologies.

© 2026 Sakr Y et al. *JAMA Network Open*. This is an open access article distributed under the terms of the CC-BY-NC-ND License, which does not permit alteration or commercial use, including those for text and data mining, AI training, and similar technologies.

eTable 4. Mode of acquisition of infection in the two study groups

		Albumin group, n (%) (N = 222)	Control group, n (%) (N = 218)
Intraabdominal			
Acquisition	Community acquired	31 (14.0)	23 (10.6)
	Hospital acquired	17 (7.7)	25 (11.5)
	ICU acquired	10 (4.5)	6 (2.8)
	Unknown	24 (10.8)	24 (11.0)
Primary vs. secondary	Primary	79 (35.6)	76 (34.9)
	Secondary	4 (1.8)	2 (0.9)
Pneumonia			
Acquisition	Community acquired	28 (12.6)	26 (11.9)
	Hospital acquired	15 (6.8)	24 (11.0)
	ICU acquired	8 (3.6)	6 (2.8)
	Unknown	1 (0.5)	1 (0.5)
Primary vs. secondary	Primary	47 (21.2)	52 (23.9)
	Secondary	5 (2.3)	5 (2.3)
Bacteremia			
Acquisition	Community acquired	15 (6.8)	16 (7.3)
	Hospital acquired	8 (3.6)	10 (4.6)
	ICU acquired	2 (0.9)	2 (0.9)
	Unknown	9 (4.1)	3 (1.4)
Primary vs. secondary	Primary	22 (9.9)	17 (7.8)
	Secondary	11 (5.0)	14 (6.4)
Urogenital			
Acquisition	Community acquired	24 (10.8)	11 (5.0)
	Hospital acquired	6 (2.7)	7 (3.2)
	ICU acquired	3 (1.4)	-
	Unknown	2 (0.9)	3 (1.4)
Primary vs. secondary	Primary	26 (11.7)	18 (8.3)
	Secondary	9 (4.1)	3 (1.4)
Gastrointestinal			
Acquisition	Community acquired	8 (3.6)	11 (5.0)
	Hospital acquired	1 (0.5)	3 (1.4)
	ICU acquired	-	2 (0.9)
	Unknown	8 (3.6)	2 (0.9)
Primary vs. secondary	Primary	16 (7.2)	18 (8.3)
	Secondary	1 (0.5)	-
Bones, soft tissue			
Acquisition	Ambulant acquired	16 (7.2)	8 (3.7)
	Hospital acquired	1 (0.5)	1 (0.5)
	Unknown	1 (0.5)	-
Primary vs. secondary	Primary	17 (7.7)	8 (3.7)
	Secondary	1 (0.5)	1 (0.5)
Thoracic infections			
Acquisition	Community acquired	2 (0.9)	3 (1.4)
	Hospital acquired	3 (1.4)	2 (0.9)
	ICU acquired	1 (0.5)	
	Unknown	1 (0.5)	1 (0.5)
Primary vs. secondary	Primary	7 (3.2)	4 (1.8)
	Secondary	-	2 (0.9)
Cardiovascular			
Acquisition	Community acquired	2 (0.9)	1 (0.5)
	Hospital acquired	-	1 (0.5)

© 2026 Sakr Y et al. *JAMA Network Open*. This is an open access article distributed under the terms of the CC-BY-NC-ND License, which does not permit alteration or commercial use, including those for text and data mining, AI training, and similar technologies.

		Albumin group, n (%) (N = 222)	Control group, n (%) (N = 218)
	Unknown	-	1 (0.5)
Primary vs. secondary	Primary	1 (0.5)	3 (1.4)
	Secondary	1 (0.5)	-
Renal			
Acquisition	Community acquired	1 (0.5)	5 (2.3)
	Hospital acquired	1 (0.5)	-
	Unknown	2 (0.9)	-
Primary vs. secondary	Primary	4 (1.8)	5 (2.3)
Skin			
Acquisition	Community acquired	1 (0.5)	3 (1.4)
	Hospital acquired	-	1 (0.5)
	Unknown	-	1 (0.5)
Primary vs. secondary	Primary	1 (0.5)	4 (1.8)
	Secondary	-	1 (0.5)
Central nervous system		-	
Acquisition	Community acquired	-	1 (0.5)
Primary vs. secondary	Primary	-	1 (0.5)
Catheter-related			
Acquisition	Community acquired	1 (0.5)	-
	Hospital/care acquired	-	4 (1.8)
Primary vs. secondary	Primary	1 (0.5)	3 (1.4)
	Secondary	-	1 (0.5)
Surgical wound			
Acquisition	Community acquired	1 (0.5)	2 (0.9)
	Hospital acquired	2 (0.9)	2 (0.9)
	ICU acquired	-	1 (0.5)
	Unknown	-	3 (1.4)
Primary vs. secondary	Primary	2 (0.9)	7 (3.2)
	Secondary	1 (0.5)	1 (0.5)

eTable 5. Serum albumin levels of patients by study time frame

Time	N	Albumin levels [g/L]*	
		Albumin group	Control group
Randomization	432	22.0 (18.2-26.7) [21.0-23.3]	22.2 (18.0-26.2) [21.6-23.4]
Day 1	421	27.0 (24.0-30.3) [26.0-28.0]	21.0 (17.0-24.1) [20.2-22.0]
Day 2-7	390	28.0 (26.0-30.0) [28.0-28.2]	19.5 (16.0-23.2) [19.0-20.0]
Day 8-28	206	29.0 (17.0-31.0) [29.0-29.2]	17.9 (14.5-22.3) [17.2-18.0]

* Data within the indicated time frame are given as median (IQR) [CI]

© 2026 Sakr Y et al. *JAMA Network Open*. This is an open access article distributed under the terms of the CC-BY-NC-ND License, which does not permit alteration or commercial use, including those for text and data mining, AI training, and similar technologies.

eTable 6. Protocol deviations in the two study groups

Type	Subtype	Albumin, N = 222		Control without albumin, N = 218	
		PD	N (%) Patients*	PD	N (%) Patients*
In-/Exclusion criteria					
	Existing end of life decision	1	1 (0.5)	-	-
	Pregnancy/lactation	2	2 (0.9)	2	2 (0.9)
	Total	3	3 (1.4)	2	2 (0.9)
Informed consent/Inclusion					
	No written informed consent (Patient died)	24	24 (10.8)	28	27 (12.4)
	Retrospective consent of representative not obtained within 72 h	26	24 (10.8)	18	17 (7.8)
	Inclusion acc. § 41 AMG, retrospective consent of representative not obtained within 72 h	10	10 (4.5)	11	11 (5.0)
	No written informed consent (Patient alive)	5	5 (2.3)	6	6 (2.8)
	Randomization before inclusion	4	4 (1.8)	1	1 (0.5)
	Other	2	2 (0.9)	3	3 (1.4)
	Inclusion after oral consent	1	1 (0.5)	2	2 (0.9)
	Retrospective consent of representative not obtained (patient alive)	2	2 (0.9)	.	
	Consent/inclusion invalid	1	1 (0.5)	-	-
	Legal representative signed consent before appointment	-	-	1	1 (0.5)
	Not defined	-	-	1	1 (0.5)
	Total	75	66 (29.7)	71	62 (28.4)
Violation study procedures					
	Secondary endpoints missing/implausible values	123	72 (32.4)	137	85 (39.0)
	Wrong dose adjustment	65	46 (20.7)	-	-
	Routine human albumin administered in addition to correctly administered study treatment	30	19 (8.6)	-	-
	No dose adjustment	11	11 (5.0)	-	-
	Daily albumin assessment at ICU not done	11	10 (4.5)	78	46 (21.1)
	Use of routine human albumin instead of study treatment	9	8 (3.6)	-	-
	Albumin assessment after first study treatment	1	1 (0.5)	-	-
	No drug account/empty bottles/remaining medication not rescued	7	7 (3.2)	-	-
	Study treatment administered in control group	-	-	2	2 (0.9)
	Inclusion acc. § 41 AMG, retrospective consent of representative not obtained within 72 h	-	-	1	1 (0.5)
	Randomization before inclusion	2	2 (0.9)	-	-
	Not defined	1	1 (0.5)	.	
	Total	260	130 (58.6)	218	110 (50.5)

© 2026 Sakr Y et al. *JAMA Network Open*. This is an open access article distributed under the terms of the CC-BY-NC-ND License, which does not permit alteration or commercial use, including those for text and data mining, AI training, and similar technologies.

		Albumin, N = 222		Control without albumin, N = 218	
**Type**	**Subtype**	**PD**	**N (%) Patients***	**PD**	**N (%) Patients***
Violation time schedule					
	Treatment start <6 h after start of septic shock	27	27 (12.2)	-	-
	Treatment start not within 2 h after randomization	16	16 (7.2)	-	-
	Randomization not within 2 h after inclusion	5	5 (2.3)	3	3 (1.4)
	Treatment start >24 h after start of septic shock	3	3 (1.4)	-	-
	Blood sample > 2 h after randomization	-	-	1	1 (0.5)
	Retrospective consent of representative not obtained within 72 h	3	3 (1.4)	-	-
	Secondary endpoints missing/implausible values	3	2 (0.9)	3	3 (1.4)
	Total	57	51 (23.0)	7	6 (2.8)
Randomization error					
	Wrong stratification	16	16 (7.2)	16	16 (7.3)
	Other	-	-	1	1 (0.5)
	Total	16	16 (7.2)	17	17 (7.8)
Other					
	No or late SAE notification	7	7 (3.2)	12	12 (5.5)
	Not defined	3	3 (1.4)	-	-
	Other	10	7 (3.2)	-	-
	Total	20	16 (7.2)	12	12 (5.5)
No deviations		-	44 (19.8)	-	67 (30.7)

*PD=protocol deviation. Multiple PDs possible per patient, hence patient numbers do not equal 100%

eTable 7. Physiologic and oxygenation parameters, fluid administration, vasopressor therapy, and procedures in the two groups at baseline and 6 hours after randomization

	Albumin		Control	
	**Baseline; mean±sd [CI]***	**6 hrs; mean±sd [CI]**	**Baseline; mean±sd [CI]**	**6 hrs; mean±sd [CI]**
Physiologic parameters				
Heart rate bpm**	122±24 [119-126]	95±21; [92-98]	124±25; [120-127]	99±21; [96-102]
Mean arterial pressure mmHg	57±9; [55-59]	75±12; [73-77]	57±10; [55-59]	73±11; [71-75]
Temperature °C	36.9±1.0; [36.7-37.1]	36.9±1.0; [36.8-37.1]	37.1±1.0; [36.9-37.3]	37.1±1.0; [36.9-37.3]
Respiratory rate /min	20±7; [19-22]	20±6; [18-21]	21±7; [20-23]	20±6; [19-22]
Oxygenation parameters				
FiO2	0.42±0.17; [0.40-0.45]	0.42±0.16; [0.39-0.44]	0.44±0.19; [0.41-0.47]	0.43±0.19; [0.41-0.47]
PaO2, mmHg	100±39; [94-106]	101±51; [94-109]	99±40; [93-105]	93±31; [89-98]
PaCO2, mmHg	41±19; [38-44]	42±27; [38-47]	40±9; [38-41]	39±9; [38-41]
SO2, %	96.0±2.7;	96.1±3.1;	96.2±2.8;	95.8±3.2;

© 2026 Sakr Y et al. *JAMA Network Open*. This is an open access article distributed under the terms of the CC-BY-NC-ND License, which does not permit alteration or commercial use, including those for text and data mining, AI training, and similar technologies.

	[95.6-96.5]	[95.6-96.5]	[95.8-96.6]	[95.3-96.3]
PaO2/FiO2 ratio	262±124; [244-279]	265±116; [249-282]	262±129; [244-281]	251±123; [233-269]
Vasopressors, norepinephrine dose, µg/kg/min, median (IQ)	0.32 (0.17-0.60)	0.28 (0.13-0.58)	0.33 (0.15-0.58)	0.32 (0.14-0.57)
Urine output, median [95% CI of median] (IQ)†				
Total	873 [700-1040] (225-1720)	230 [190-270] (60-524)	900 [760-1080] (381-1990)	200 [160-300] (60-480)
ml/hr	36 [29-44]; (9-72)	38 [31-45]; (10-87)	38 [31-45]; (16-83)	33 [26-50]; (10-80)
Volume therapy, n (%)†				
Crystalloid solutions	204 (91.9)	171 (77.0)	205 (94.0)	183 (83.9)
Colloid solutions	51 (23.0)	15 (6.8)	70 (32.1)	20 (9.2)
Blood transfusion	52 (23.4)	44 (19.8)	38 (17.4)	25 (11.5)
Procedures, n (%)				
Invasive mechanical ventilation	143 (64.4)	142 (64.8)	151 (69.3)	142 (65.1)
Renal replacement therapy	44 (19.8)	56 (25.6)	33 (15.1)	46 (21.1)

* Unless otherwise specified values are mean ± standard deviation [95% confidence interval of mean]; Abbreviations: CI, confidence interval; sd, standard deviation.

Baseline values obtained as the worst value +/- 1 hour at screening, 6 hr values obtained as the worst value +/- 1 hour after 6 hours after randomization.

Missing values (albumin group baseline/albumin group 6 hours after randomization/control group baseline/control group 6 hours after randomization): heart rate: 0/4/0/6, mean arterial pressure: 1/4/1/6, Temperature: 34/34/36/26, respiratory rate:8/17/13/25, FiO2: 14/18/18/22, PaO2 22/19/23/25, PaCO2: 28/28/35/36, SO2: 28/23/25/31, PaO2/FiO2 ratio: 24/20/25/27, norepinephrine dose: 1/3/3/6, urine output: 1/2/1/0, invasive mechanical ventilation: 0/3/0/0, renal replacement therapy: 0/3/0/0

** Maximum value at base line and worst value +/-1 hour after 6 hours of randomization

† Baseline refers to the 24 hours preceding randomization, 6 hrs refers to the 6 hours following randomization

© 2026 Sakr Y et al. *JAMA Network Open*. This is an open access article distributed under the terms of the CC-BY-NC-ND License, which does not permit alteration or commercial use, including those for text and data mining, AI training, and similar technologies.

eTable 8. Mortality rates in the two study groups according to baseline lactate, baseline SOFA, SAPS II, and APACHE II values

**Outcome**	**Albumin group; n/total n (%)**			**Control group; n/total n (%)**		
**Baseline lactate – mmol/L**	**≤ 8**	**> 8**		**≤ 8**	**> 8**	
Death at 90 days — n/total n (%)	65/162 (40)	26/48 (54)		68/157 (43)	28/52 (54)	
At 28 days	48/164 (29)	18/49 (37)		52/158 (33)	28/52 (54)	
At 60 days	58/162 (36)	24/49 (49)		67/158 (42)	28/52 (54)	
In the intensive care unit	50/162 (31)	18/48 (38)		47/153 (31)	24/51 (47)	
In hospital	59/155 (38)	23/48 (48)		62/150 (41)	28/51 (55)	
**Baseline SOFA [0-24]**	**2-9**	**10-14**	**15-24**	**2-9**	**10-14**	**15-24**
Death at 90 days — n/total n (%)	20/72 (28)	56/115 (49)	13/16 (81)	22/78 (28)	53/101 (52)	15/20 (75)
At 28 days	9/73 (12)	43/116 (37)	12/16 (75)	16/79 (20)	44/101 (44)	14/20 (70)
At 60 days	16/72 (22)	51/116 (44)	13/16 (81)	22/79 (28)	52/101 (51)	15/20 (75)
In the intensive care unit	13/73 (18)	44/113 (39)	11/16 (69)	11/77 (14)	42/98 (43)	13/19 (68)
In hospital	17/71 (24)	52/109 (48)	12/16 (75)	19/75 (25)	50/96 (52)	15/20 (75)
**Baseline SAPS II [0-163]**	**≤39**	**40-79**	**80-163**	**≤39**	**40-79**	**80-163**
Death at 90 days — n/total n (%)	6/29 (21)	68/150 (45)	8/9 (88.9)	7/32 (22)	64/141 (45)	12/12 (100)
At 28 days	3/29 (10)	47/152 (31)	7/9 (77.8)	5/32 (16)	54/142 (38)	10/12 (83)
At 60 days	5/29 (17)	60/151 (40)	8/9 (88.9)	7/32 (22)	63/142 (44)	12/12 (100)
In the intensive care unit	5/28 (18)	50/150 (33)	8/9 (88.9)	2/31 (6)	48/137 (35)	11/12 (92)
In hospital	5/27 (19)	61/146 (42)	8/8 (100.0)	6/30 (20)	61/136 (45)	11/12 (92)
**Baseline APACHE II [0-71]**	≤19	20-29	≥30	≤19	20-29	≥30
Death at 90 days — n/total n (%)	21/78 (27)	39/81 (48)	29/43 (67)	21/75 (28)	48/94 (51)	24/30 (80)
At 28 days	10/78 (13)	31/83 (37)	24/43 (56)	16/76 (21)	40/94 (43)	22/30 (73)
At 60 days	15/78 (19)	37/82 (45)	28/43 (65)	21/76 (28)	47/94 (50)	24/30 (80)
In the intensive care unit	16/76 (21)	28/82 (34)	23/43 (54)	13/73 (18)	36/92 (39)	20/29 (69)
In hospital	19/75 (25)	35/78 (45)	26/43 (61)	18/72 (25)	46/90 (51)	24/30 (80)

© 2026 Sakr Y et al. *JAMA Network Open*. This is an open access article distributed under the terms of the CC-BY-NC-ND License, which does not permit alteration or commercial use, including those for text and data mining, AI training, and similar technologies.

eTable 9. Frequency of adverse events (AEs) in the two study groups

Event	Albumin (n=222)		Control without albumin (n=218)	
	No. Events	Patients* N (%)	No. Events	Patients* N (%)
MedDRA category				
General disorders	158	96 (43.2)	117	81 (37.2)
Gastrointestinal disorders	22	16 (7.2)	13	13 (6.0)
Respiratory, thoracic and mediastinal disorders	17	16 (7.2)	9	7 (3.2)
Cardiac disorders	17	11 (5.0)	16	13 (6.0)
Metabolism and nutrition disorders	12	11 (5.0)	7	6 (2.8)
Laboratory derangement	6	4 (1.8)	.	
Infections	6	5 (2.3)	5	5 (2.3)
Blood and lymphatic system disorders	5	5 (2.3)	1	1 (0.5)
Nervous system disorders	5	5 (2.3)	8	7 (3.2)
Injury, poisoning and procedural complications	4	3 (1.4)	8	6 (2.8)
Vascular disorders	4	4 (1.8)	8	7 (3.2)
Renal and urinary disorders	3	3 (1.4)	1	1 (0.5)
Skin and subcutaneous tissue disorders	3	3 (1.4)	1	1 (0.5)
Surgical and medical procedures	2	2 (0.9)	.	
Psychiatric disorders	1	1 (0.5)	4	4 (1.8)
Endocrine disorders	1	1 (0.5)	.	
Hepatobiliary disorders	1	1 (0.5)	.	
Musculoskeletal and connective tissue disorders	.		2	1 (0.5)
Neoplasms	.		2	1 (0.5)
Total AEs	267	121 (54.5)	202	105 (48.1)
Severity of AEs				
Severe	58	38 (17.1)	52	41 (18.8)
Serious	44	30 (13.5)	42	34 (15.6)
Fatal	6	6 (2.7)	12	12 (5.5)

Note: Individual patients may have experienced several events, hence patient numbers do not necessarily total 100%

eTable 10. Frequency of sepsis-related events in the two study groups

Event	Albumin (n=222)		Control without albumin (n=218)	
	No. Events	Patients N (%)	No. Events	Patients N (%)
Sepsis-related events				
Cardiovascular	277	183 (82.4)	252	176 (80.7)
Respiratory	239	160 (72.1)	227	159 (72.9)
Renal	159	123 (55.4)	186	138 (63.3)
Hematologic	159	117 (52.7)	149	118 (54.1)
Hepatic	124	95 (42.8)	105	82 (37.6)
Neurologic	94	69 (31.1)	87	62 (28.4)
Low leukocyte count/high fever	36	31 (14.0)	37	34 (15.6)
Total	1088	206 (92.8)	1043	209 (95.9)

Note: Individual patients may have experienced several events, hence patient numbers do not necessarily total 100%

© 2026 Sakr Y et al. *JAMA Network Open*. This is an open access article distributed under the terms of the CC-BY-NC-ND License, which does not permit alteration or commercial use, including those for text and data mining, AI training, and similar technologies.

eFigure 1. Study flow chart of interventions

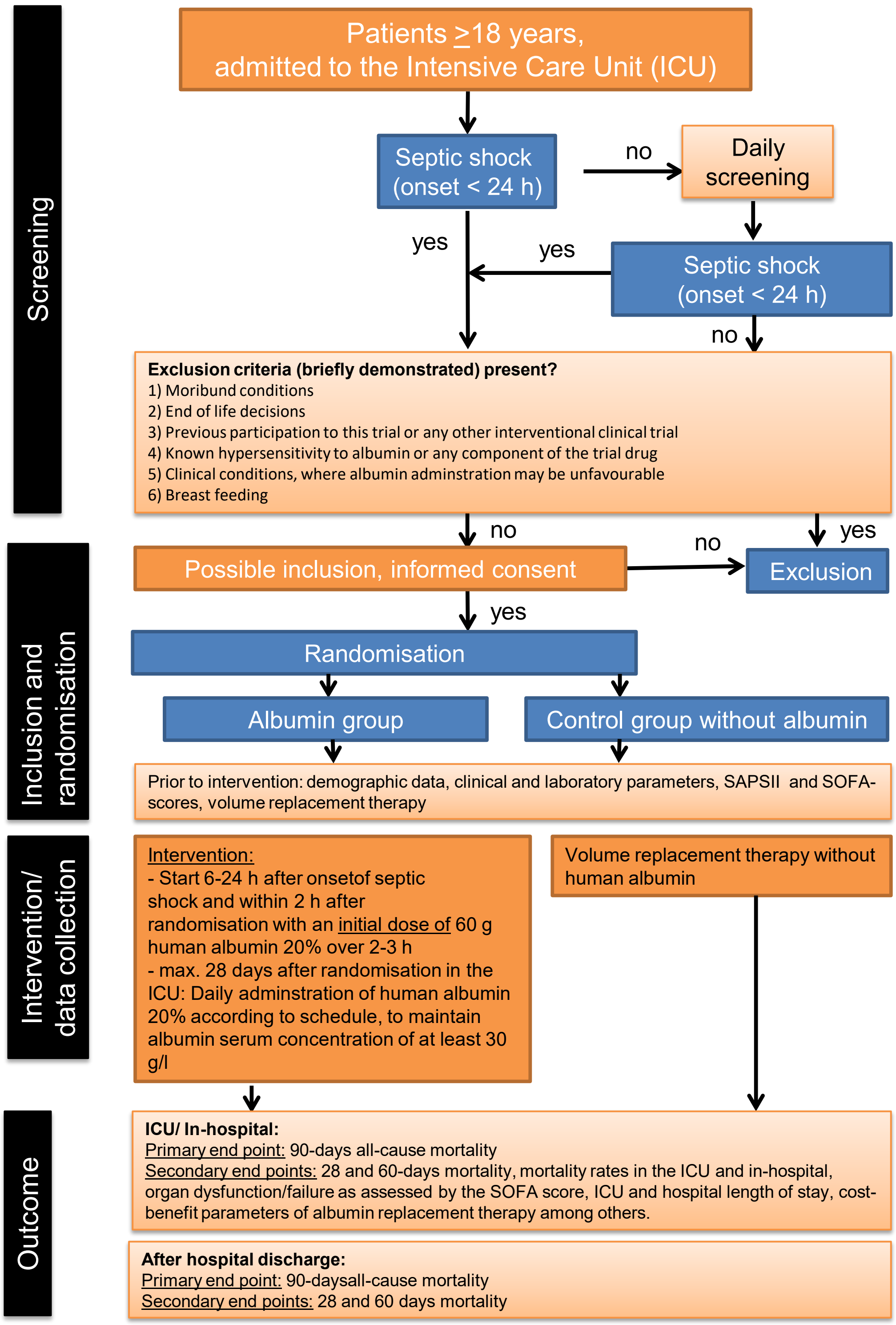

SAPS: Simplified Acute Physiology Score, SOFA: Sequential Organ Failure Assessment

© 2026 Sakr Y et al. *JAMA Network Open*. This is an open access article distributed under the terms of the CC-BY-NC-ND License, which does not permit alteration or commercial use, including those for text and data mining, AI training, and similar technologies.

eFigure 2. Box plot showing mean arterial pressure in the two groups measured at screening, 6 hours after randomization, and then daily for 28 days in the ICU

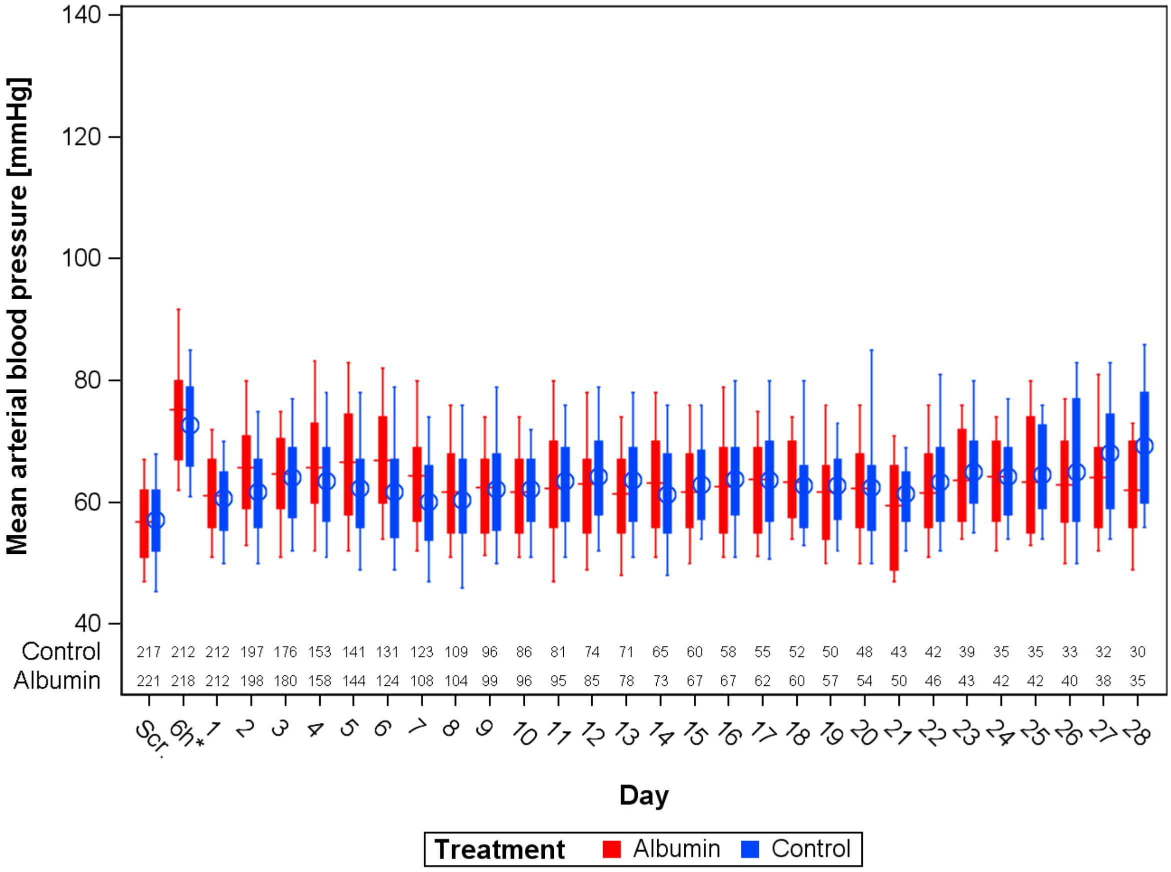

© 2026 Sakr Y et al. *JAMA Network Open*. This is an open access article distributed under the terms of the CC-BY-NC-ND License, which does not permit alteration or commercial use, including those for text and data mining, AI training, and similar technologies.

eFig 3. Box plot showing the fluid intake in the two groups at screening, 6 hours after randomization, and then daily for 28 days in the ICU

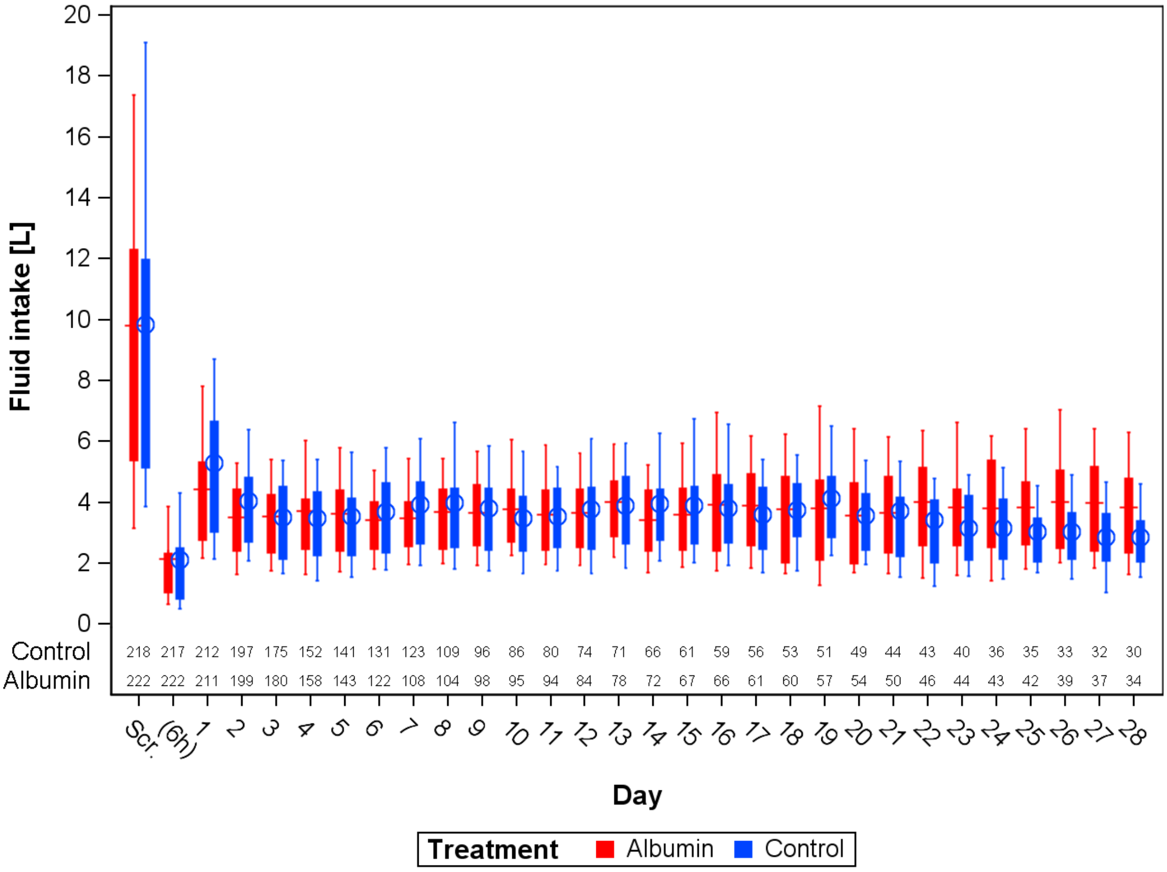

© 2026 Sakr Y et al. *JAMA Network Open*. This is an open access article distributed under the terms of the CC-BY-NC-ND License, which does not permit alteration or commercial use, including those for text and data mining, AI training, and similar technologies.

eFig 4. Box plot showing the fluid output (excluding perspiration) in the two groups at screening, 6 hours after randomization, and then daily for 28 days in the ICU

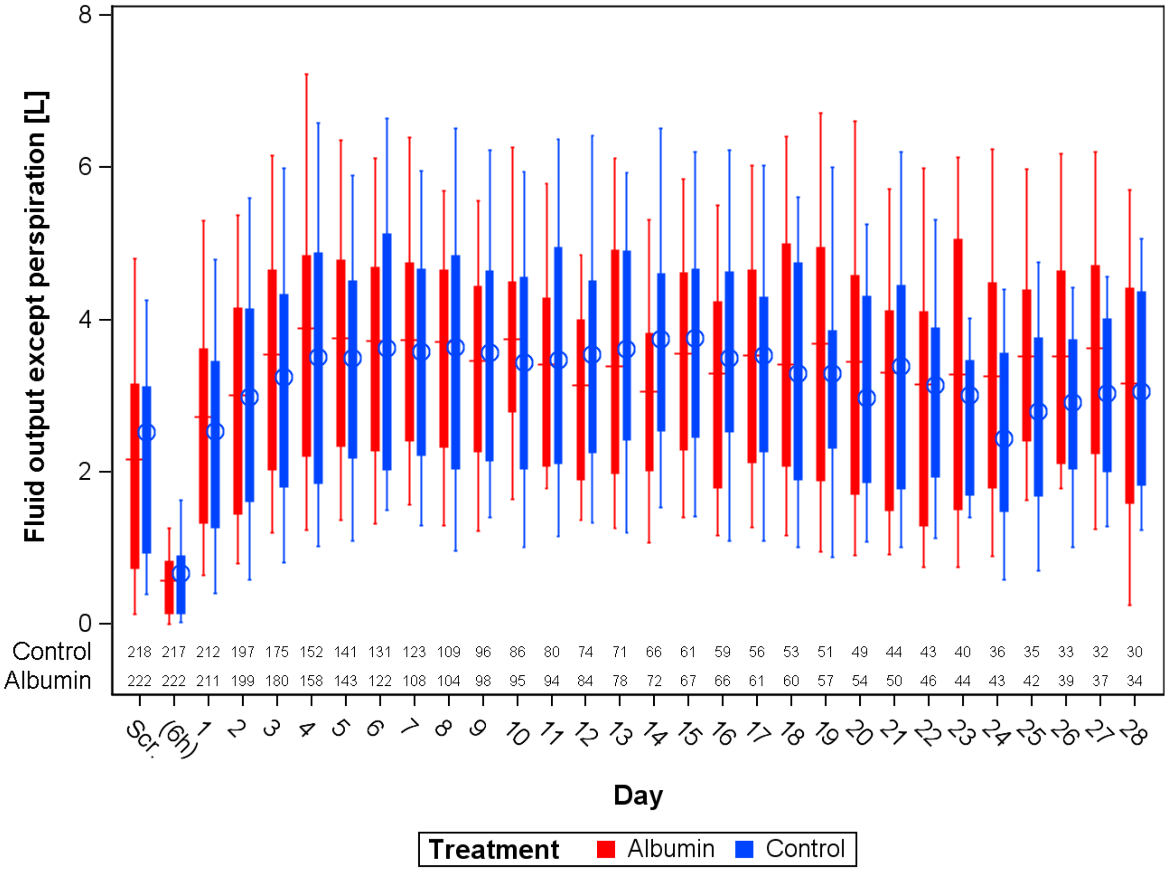

© 2026 Sakr Y et al. *JAMA Network Open*. This is an open access article distributed under the terms of the CC-BY-NC-ND License, which does not permit alteration or commercial use, including those for text and data mining, AI training, and similar technologies.
