# Supplementary material for: Albumin Replacement Therapy in Septic Shock: A Randomized Clinical Trial
Source: JAMA Netw Open. 2026 Feb 19;9(2):e2559297. doi: 10.1001/jamanetworkopen.2025.59297 (PMC12921518; doi:10.1001/jamanetworkopen.2025.59297)
Supplement: Supplement 3. — The SepNet Critical Care Trials Group and ARISS investigators [file jamanetwopen-e2559297-s003.pdf]

Supplemental Online Content: Nonauthor Collaborators
*First name, last name, and suffix (if applicable) are required and will appear in PubMed.

*Group Name(s): ARISS investigators and SepNet Critical Care Trials Group							
***First Name and Middle Initial(s)**	***Last Name**	***Suffix (eg, Jr, III)**	Academic Degrees	Institution	Location (city, state/province, country)	Role or Contribution, eg, chair, principal investigator	Group (if more than 1 Group listed in the byline) and/or Subgroup (eg, Steering Committee)
Peter	Appelt		Dr. med.	University of Leipzig Medical Center	Leipzig, Germany	recruiting center	ARISS investigators
Tobias	Becher		Dr. med.	University Hospital Schleswig-Holstein	Kiel, Germany	recruiting center	ARISS investigators
Petra	Bischoff		Dr. med.	University Hospitals of the Ruhr-University of Bochum	Bochum, Germany	recruiting center	ARISS investigators
Josef M.	Briegel		Dr. med.	Ludwig-Maximilians-University (LMU)	Munich, Germany	recruiting center	SepNet Critical Care Trials Group
Phillipp	Deetjen		Dr. med.	Universitätsklinikum Augsburg	Augsburg, Germany	recruiting center	ARISS investigators
Jahn	Ebbinghaus		Dr. med.	Vivantes Humboldt-Klinikum	Berlin, Germany	recruiting center	ARISS investigators
Stefan	Ehrentraut		Dr. med.	University Hospital Bonn	Bonn, Germany	recruiting center	ARISS investigators
Gunnar	Elke		Dr. med.	University Hospital Schleswig-Holstein	Kiel, Germany	recruiting center	SepNet Critical Care Trials Group
Fritz	Fiedler		Dr. med.	St. Elisabeth-Krankenhaus Hohenlind	Cologne, Germany	recruiting center	SepNet Critical Care Trials Group
Sandra	Frank		Dr. med.	Ludwig-Maximilians-University (LMU)	Munich, Germany	recruiting center	SepNet Critical Care Trials Group
Michael	Fritzenwanger		Dr. med.	Jena University Hospital	Jena, Germany	recruiting center	ARISS investigators
Luciano	Gattinoni		Dr. med.	University Medical Center Göttingen	Göttingen, Germany	recruiting center	ARISS investigators
Ulf	Günther		Dr. med.	Klinikum Oldenburg	Oldenburg, Germany	recruiting center	ARISS investigators
Immanuel J.	Handerer		Dr. med.	University Hospital Magdeburg	Magdeburg, Germany	recruiting center	ARISS investigators
Ulrich H.	Frey		Dr. med.	University Hospitals of the Ruhr-University of Bochum	Bochum, Germany	recruiting center	ARISS investigators
Marie	Holubek			University Hospital Magdeburg	Magdeburg, Germany	recruiting center	ARISS investigators
David	Jacob		Dr. med.	University Hospital Magdeburg	Magdeburg, Germany	recruiting center	ARISS investigators
Barbara	Kapfer		Dr. med.	Klinikum rechts der Isar, Technical University of Munich	Munich, Germany	recruiting center	ARISS investigators

*First name, last name, and suffix (if applicable) are required and will appear in PubMed.

***First Name and Middle Initial(s)**	***Last Name**	***Suffix (eg, Jr, III)**	Academic Degrees	Institution	Location (city, state/province, country)	Role or Contribution, eg, chair, principal investigator	Group (if more than 1 Group listed in the byline) and/or Subgroup (eg, Steering Committee)
Ingmar	Lautenschläger		Dr. med.	University Hospital Schleswig-Holstein	Kiel, Germany	recruiting center	ARISS investigators
Josefa	Lehmke		Dr. Dr.	Vivantes Humboldt-Klinikum	Berlin, Germany	recruiting center	ARISS investigators
Matthias	Lindner		Dr. med.	University Hospital Schleswig-Holstein	Kiel, Germany	recruiting center	ARISS investigators
Uwe	Lodes		Dr. med.	University Hospital Magdeburg	Magdeburg, Germany	recruiting center	ARISS investigators
Jan	Martin		Dr. med.	Klinikum rechts der Isar, Technical University of Munich	Munich, Germany	recruiting center	ARISS investigators
Melanie	Meersch-Dini		Dr. med.	University Medical Centre of Münster	Münster, Germany	recruiting center	SepNet Critical Care Trials Group
Dominik	Michalski		Dr. med.	University of Leipzig Medical Center	Leipzig, Germany	recruiting center	ARISS investigators
Onnen	Mörer		Dr. med.	University Medical Center Göttingen	Göttingen, Germany	recruiting center	ARISS investigators
Bastian	Pasieka			University of Leipzig Medical Center	Leipzig, Germany	recruiting center	ARISS investigators
Rüdiger	Pfeifer		Dr. med.	Jena University Hospital	Jena, Germany	recruiting center	ARISS investigators
Stefan	Rasche		Dr. med.	University of Leipzig Medical Center	Leipzig, Germany	recruiting center	ARISS investigators
Florian	Roghmann		Dr. med.	University Hospitals of the Ruhr-University of Bochum	Bochum, Germany	recruiting center	ARISS investigators
Christian	Scheer		Dr. med.	Greifswald University Medicine	Greifswald, Germany	recruiting center	SepNet Critical Care Trials Group
Stefan	Schering		Dr. med.	University of Leipzig Medical Center	Leipzig, Germany	recruiting center	ARISS investigators
Jens-Christian	Schewe		Dr. med.	University Hospital Bonn	Bonn, Germany	recruiting center	ARISS investigators
Alexander	Schmeisser		Dr. med.	University Hospital Magdeburg	Magdeburg, Germany	recruiting center	ARISS investigators
Christian	Schulze		Dr. med.	Jena University Hospital	Jena, Germany	recruiting center	ARISS investigators
Metin	Senkal		Dr. med.	University Hospitals of the Ruhr-University of Bochum	Bochum, Germany	recruiting center	ARISS investigators

Supplemental Online Content: Nonauthor Collaborators
*First name, last name, and suffix (if applicable) are required and will appear in PubMed.

***First Name and Middle Initial(s)**	***Last Name**	***Suffix (eg, Jr, III)**	Academic Degrees	Institution	Location (city, state/province, country)	Role or Contribution, eg, chair, principal investigator	Group (if more than 1 Group listed in the byline) and/or Subgroup (eg, Steering Committee)
Philipp	Simon		Dr. med.	University of Leipzig Medical Center	Leipzig, Germany	recruiting center	SepNet Critical Care Trials Group
Petra	Tepaß		Dr. med.	St. Elisabeth-Krankenhaus Hohenlind	Cologne, Germany	recruiting center	ARISS investigators
Katja	Wartenberg		Dr. med.	University of Leipzig Medical Center	Leipzig, Germany	recruiting center	ARISS investigators
Lorenz	Weidhase		Dr. med.	University of Leipzig Medical Center	Leipzig, Germany	recruiting center	ARISS investigators
Carsten	Weissbrich		Dr. med.	University Hospital Bonn	Bonn, Germany	recruiting center	ARISS investigators
Julian	Westphal		Dr. med.	Jena University Hospital	Jena, Germany	recruiting center	ARISS investigators
Andreas	Weyland		Dr. med.	Klinikum Oldenburg	Oldenburg, Germany	recruiting center	ARISS investigators
Alexander	Zarbock		Dr. med.	University Medical Centre of Münster	Münster, Germany	recruiting center	SepNet Critical Care Trials Group
