# Supplementary material for: Albumin Replacement Therapy in Septic Shock: A Randomized Clinical Trial
Source: JAMA Netw Open. 2026 Feb 19;9(2):e2559297. doi: 10.1001/jamanetworkopen.2025.59297 (PMC12921518; doi:10.1001/jamanetworkopen.2025.59297)
Supplement: Supplement 4. — Data Sharing Statement [file jamanetwopen-e2559297-s004.pdf]

Data Sharing Statement

Sakr. Albumin Replacement Therapy in Septic Shock. *JAMA Netw Open*. Published February 19, 2026. doi:10.1001/jamanetworkopen.2025.59297

Data
Additional Information: NCT03869385. Registration date: 18/07/2019
Data available: No
